# ^17^O NMR Spectroscopy in Lithium-Ion Battery Cathode Materials: Challenges and Interpretation

**DOI:** 10.1021/jacs.2c02927

**Published:** 2022-10-06

**Authors:** Euan N. Bassey, Philip J. Reeves, Ieuan D. Seymour, Clare P. Grey

**Affiliations:** † Department of Chemistry, University of Cambridge, Lensfield Road, CambridgeCB2 1EW, United Kingdom; ‡ Department of Materials, Imperial College London, South Kensington Campus, LondonSW7 2AZ, United Kingdom

## Abstract

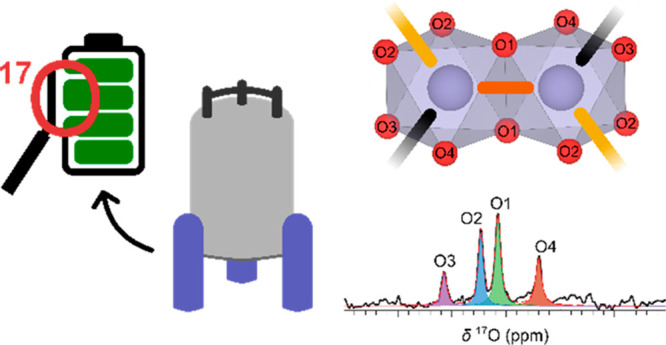

Modern studies of lithium-ion battery (LIB) cathode materials employ a large range of experimental and theoretical techniques to understand the changes in bulk and local chemical and electronic structures during electrochemical cycling (charge and discharge). Despite its being rich in useful chemical information, few studies to date have used ^17^O NMR spectroscopy. Many LIB cathode materials contain paramagnetic ions, and their NMR spectra are dominated by hyperfine and quadrupolar interactions, giving rise to broad resonances with extensive spinning sideband manifolds. In principle, careful analysis of these spectra can reveal information about local structural distortions, magnetic exchange interactions, structural inhomogeneities (Li^+^ concentration gradients), and even the presence of redox-active O anions. In this Perspective, we examine the primary interactions governing ^17^O NMR spectroscopy of LIB cathodes and outline how ^17^O NMR may be used to elucidate the structure of pristine cathodes and their structural evolution on cycling, providing insight into the challenges in obtaining and interpreting the spectra. We also discuss the use of ^17^O NMR in the context of anionic redox and the role this technique may play in understanding the charge compensation mechanisms in high-capacity cathodes, and we provide suggestions for employing ^17^O NMR in future avenues of research.

## 1 Motivation

Lithium-ion batteries (LIBs) play a critical role in enabling future sustainable energy sources by storing energy for grid usage and powering devices and transportation.^1−3^ To ensure that electric vehicles (EVs) powered with LIB technologies are competitive with those that use fossil-fuel energy sources, LIB components must be low-cost and environmentally sustainable (ideally, fully recyclable)^4^ while also achieving high capacities over long lifetimes. At present, a major bottleneck to high capacities, long lifetimes, and cost is the cathode.^5^ As such, multiple research initiatives have sought to identify, develop, and optimize cathode materials to improve the electrochemical performance of LIBs.

## 2 Introduction

**Figure 1:**
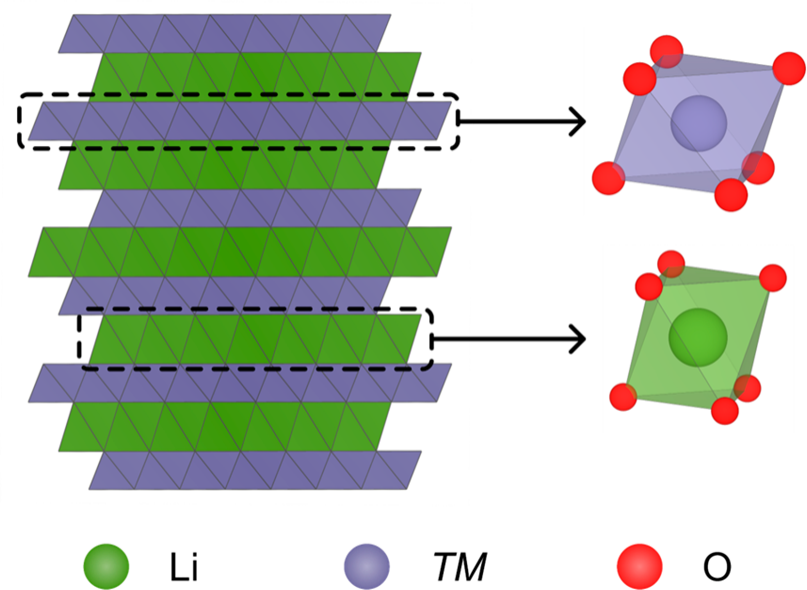
Crystal structure of a layered LIB cathode, LiTMO_2_, with the layers and local *TM* and Li^+^ coordination polyhedra highlighted.

Layered LIB cathodes, with a general formula Li*TM*O_2_ (*TM* = transition metal), are perhaps the most promising class of cathode materials currently available and, with related materials, are the focus of this Perspective.^5−13^ Here, edge-sharing *TM*O_6_ octahedra are arranged into “*TM*O_2_” layers, with Li^+^ cations in the interlayer spaces [Figure 1]. The structures are commonly described according to the notation used by Delmas et al.:^14^ a letter denoting the coordination environment of Li^+^ (O for octahedral, T for tetrahedral, and P for prismatic) and a number describing how many distinct *TM*O_2_ layers there are per unit cell. For example, O3 describes a layered cathode with octahedrally coordinated Li^+^ ions and three distinct layers per unit cell. A second notation uses the crystallographic symmetry of the cell, the number denoting the order in which the phase is found on cycling the battery. For example, on delithiating pristine LiCoO_2_, an O3 phase in Delmas notation or H1 (hexagonal) phase in the latter notation, a new O3 phase forms, denoted O3′ or H2. Intergrowth phases can similarly form, in which two distinct stacking sequences are combined in an ordered manner, and again both notations are used. For example, H1-3 describes an intergrowth of the O1 and O3 phases.

To understand the electrochemical properties of the cathode, the bulk and local structural changes that take place during cycling are evaluated, so that a clear picture of the redox mechanisms and phase changes induced during cycling may be constructed. These studies commonly use techniques such as *ex situ* or *operando* X-ray diffraction (XRD),^15−17^ X-ray absorption spectroscopy (XAS),^18−20^ and *ab initio* calculations.^21−25^ Recent studies of local structure have also used solid-state nuclear magnetic resonance (NMR) spectroscopy, owing to the high natural abundance and receptivity of (in particular) NMR-active ^6^Li and ^7^Li nuclei.^16,17,26−28^ While ^17^O NMR has proven an invaluable characterization technique in materials chemistry,^29−36^ and despite oxygen being the primary anion in LIB cathodes, there are, however, comparatively many fewer studies that have used ^17^O NMR.^37−41^

In this Perspective, we assess how ^17^O NMR has been used to understand the local (chemical and electronic) structures of LIB cathodes in both pristine and electrochemically cycled materials. In some cathode materials, capacities exceeding those of conventional *TM* redox couples and voltages above those of the *TM* redox couples have been observed. Such capacities have been assigned to redox reactions involving O^2–^ anions (henceforth O or anion redox). We therefore also examine how ^17^O NMR can help to report the changes in ionicity and covalency of the *TM*–O bonds during charge and discharge and ultimately provide important clues about the nature of the oxygen species. In order to appreciate the challenges associated with acquiring ^17^O NMR spectra, we start by discussing some of the practical and theoretical aspects associated with ^17^O NMR spectroscopy.

## 3 The Need for Isotopic Enrichment

^17^O is the only NMR-active nucleus of oxygen. With a natural abundance of 0.037%, enrichment is generally required to achieve a good signal-to-noise ratio.^31^ Enrichment is expensive, thus ^17^O NMR should be used judiciously. Syntheses of enriched LIB cathodes can also be challenging, owing to the small scale (typically <100 mg) and the subtle differences in conditions used to prepare them, as compared to “natural-abundance” syntheses. For example, enrichment is often performed either as an annealing step in a static ^17^O_2_ gas atmosphere or by heating ^17^O-enriched starting materials in a static inert atmosphere, while a “normal” synthesis might use air or flowing O_2_ gas. Hydrothermal methods using H_2_^17^O have also been employed but require careful optimization.^38,42^

## 4 Dominant Interactions in ^17^O NMR Spectroscopy

### 4.1 Chemical Shift

The chemical shift, which arises from the shielding of the applied magnetic field by electrons surrounding the nucleus, spans a vast range for ^17^O (approximately −100 ppm to +2500 ppm^43,44^); by contrast the ^6,7^Li chemical shift spans less than 10 ppm. While the chemical shift rarely dominates the observed ^17^O shifts in Li*TM*O_2_ cathodes due to the presence of paramagnetic centers (either as-synthesized or formed on cycling), it may still need to be accounted for in these paramagnetic systems.

### 4.2 Hyperfine Shift

As alluded to above, perhaps the most important consideration when acquiring ^17^O NMR of LIB cathode materials is the effect of paramagnetism.^26,28^ The hyperfine interaction between unpaired electron density (in Li*TM*O_2_, unpaired electrons on the *TM* centers) and nuclear spins in a material generally results in fast nuclear relaxation times, broad signals, and large in magnitude shifts.^26,28,45^

**Figure 2:**
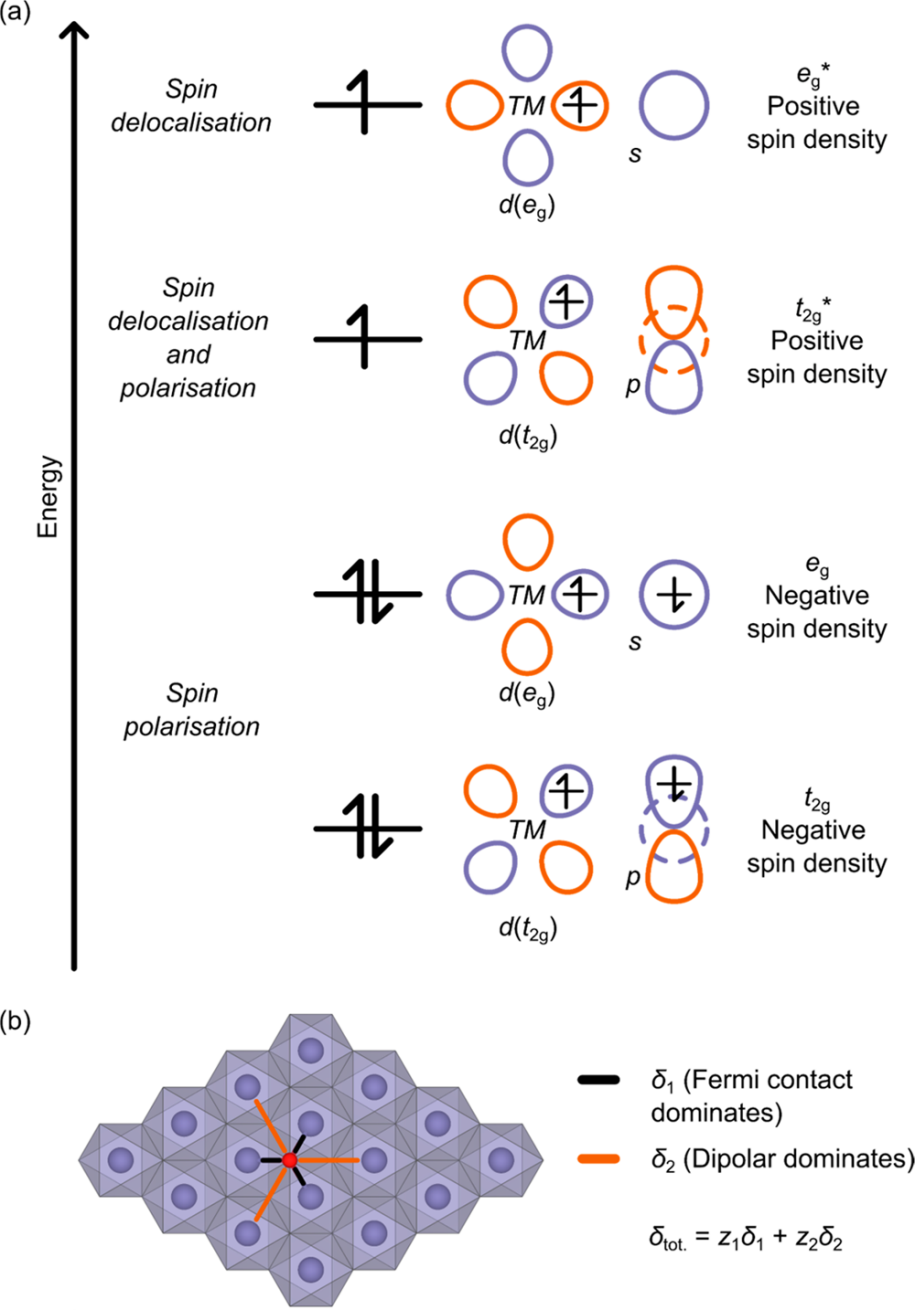
Illustrations of the hyperfine interaction in NMR. (a) The Fermi contact (through-bond) interaction of unpaired electron density on *TM* cations with a neighboring O nucleus. The bonding (*t*_2g_ and *e*_g_) and anti-bonding (*t*_2g_* and *e*_g_*) combinations of *TMd* orbitals and *s* or *p* orbitals on O result in different spin density transfer processes. Bonding combinations cause spin polarization of electrons in filled *s* orbitals on O, while anti-bonding combinations cause spin delocalization (and polarization, in the case of the *t*_2g_* interaction). The sign of the spin density induced at the *s* orbitals (which defines the Fermi contact shift) is indicated to the right. (b) The interaction of the O nucleus with the nearest-neighbor and next-nearest-neighbor unpaired electron spins in the TMO_2_ layer (of which there are *z*_1_ and *z*_2_) of a typical layered LIB cathode. These paths induce hyperfine shifts of δ_1_ and δ_2_, respectively, giving a total shift of δ_tot_.

The large shifts are invariably dominated by the Fermi contact shift, which arises from the interaction between unpaired electronic spin density at the nucleus and the nuclear spin under observation. In practice, this arises from transfer of unpaired electron density from paramagnetic ions to *s* orbitals on O [Figure 2a,b]. The *TMd* and O orbitals interact with each other to give fully occupied bonding orbitals (dominated by O orbitals, but still containing some *TM* character; bottom of Figure 2a) and either empty or partially filled anti-bonding or non-bonding orbitals (dominated by *TM* orbitals but with some O character, top of Figure 2a).

In the case of the bonding combination of *t*_2g_ and O orbitals, the unpaired electrons in the *t*_2g_* orbitals or indeed any *TM* orbital (whose spin is arbitrarily chosen as “up” in the proceeding argument) polarize(s) the fully occupied orbital such that the spins of electrons circulating nearby the *TM* center are in the same direction as those on the *TM*. This interaction is mediated via the exchange interaction, where electrons with the same spin occupy different regions of space, thus reducing the repulsion between them and lowering the energy of the system. Since the electron near the *TM* is polarized “up”, the nearby O is polarized “down”, because the two electrons occupying this orbital are spin-paired. This in turn polarizes the electrons occupying the *s* orbitals on O (again via the exchange interaction) such that these *s* orbitals align anti-parallel to the *TM* spin. This is denoted “negative” spin density (as it is opposite in sign to the *TM*, arbitrarily labeled as “positive”). By contrast, if the anti-bonding version of the same (*t*_2g_) orbital is partially occupied, then electron density is delocalized directly over the *TM* and O centers, such that the sign of the electron spin is preserved regardless of which center the electron sits nearer. This again polarizes the *s* orbitals, but now with “positive” spin density, where the electrons in O *s* orbitals are preferentially aligned with the *TM* spins. Note that if this anti-bonding combination is fully occupied, then the same argument as in the bonding combination applies, while if it is vacant, then negligible spin delocalization or polarization occurs.

The *TMe*_g_ orbitals can overlap directly with the O *s* orbitals: In the case of the bonding combination, the O *s* acquires negative spin density (again, from spin pairing), while the anti-bonding combination results in positive spin density, again due to delocalization of an unpaired spin across both centers. Note that in all cases discussed above, O is formally diamagnetic and has a complete outer shell of electrons, while the *TM* has a partially filled *d* shell and is (generally) paramagnetic. The polarization of spin density does not alter oxidation states or orbital populations, but merely the distribution (relative populations) of spins. The net unpaired spin at O results in the strong hyperfine interaction.

A robust method for analyzing and assigning the complex spectra of paramagnetic materials is bond pathway analysis, developed originally to analyze ^6,7^Li and later ^31^P spectra.^46,47^ Here, the overall shift of a nucleus is assumed to be given by the sum of the shifts induced by each paramagnetic nearest and next-nearest *TM* neighbor [Figure 2b]. This generally holds true for Li*TM*O_2_ systems.

The individual shift contributions depend on the chemical identity of the *TM* cation, its oxidation state (which defines the number of electrons and often the degree of covalency or orbital overlap), and the bond angles and distances between the *TM* cation and nucleus of interest. Deviations of the total shift from this sum occur for significant deviations away from ideal 180° and 90° bond pathways. Interactions either between different *TM*s, or within the same ion but between the orbital and spin angular momenta, *L* and *S*, respectively, can complicate the analysis, and the reader is directed to ref (45) for a more in-depth discussion of these effects. In the case of the latter, when electrons occupy orbitals with (often only partially quenched) orbital angular momentum, *L* and *S* couple to give a set of electron spin microstates whose energies are modified from the spin-only picture. This, depending on the site symmetry, can lead to an anisotropic magnetic susceptibility, and in turn a pseudo-contact shift, which also needs to be taken into account.

The temperature dependence of the time-averaged electron spin moment is reflected in the hyperfine shift: as the moment size increases, the magnitude of the shifts increases, too. These shifts may be calculated via hybrid density functional theory (DFT) calculations and rationalized using the Goodenough–Kanamori rules (see later).^48−50^ Through careful modeling, these complex spectra can be appropriately assigned to reveal valuable information about the local environments in a material.

While bond pathway analysis of ^6^Li and ^7^Li NMR spectra is well-established, the situation is more complex for ^17^O NMR, Since O is directly bonded to *TM* cations, the shifts induced are generally significantly larger, and the overall shift is often composed of several competing Fermi contact interactions with unpaired spins on several nearby *TM* cations.^40^ Typical (calculated) bond pathway shifts for O bound to different paramagnetic centers are shown in Table 1. These shifts can provide considerable insight into local magnetic exchange couplings between *TM* cations and therefore act as a local handle on the electronic spin density distribution in the material.

**Table 1:** Nearest-Neighbor ^17^O Fermi Contact Shift Bond Pathways for Different *TM* Cations Bound to O and Quadrupolar Coupling Constants, *C*_Q_, from Ref (47)^a^

*TM*–O path	bond pathway (ppm)	ref
Mn^4+^–O	900–1100^b^	(32)
Ni^3+^–O (long)	12500	(108)
Ni^3+^–O (short)	2300	(108)


fitted *C*_Q_ (MHz)	calcd *C*_Q_ (MHz)	ref
4.40–4.55	4.50–4.60	(47)

a The Mn^4+^–O pathways and *C*_Q_ values were determined for Li_2_MnO_3_; the Ni^3+^–O (long/short) pathways for Li[Ni_0.8_Co_0.15_Al_0.05_]O_2_.

b The bond pathway strongly depends on the bond length and the Mn–O–Mn angle.

An additional, through-space, anisotropic component of the hyperfine interaction, the dipolar hyperfine interaction, contributes to the hyperfine shift observed but is usually smaller than the Fermi contact interaction.^45^ The dipolar interaction does, however, generate a broad sideband manifold under magic angle spinning (MAS) which spans several thousand ppm, meaning that a single radiofrequency pulse cannot excite the entire spectrum.^29,40,41^ As a result, variable-offset cumulative spectra (VOCS) experiments are often required to excite the entire spectrum (see the Supporting Information (SI)).^51^

In addition to a strong hyperfine interaction, the short *TM*–O distances result in stronger paramagnetic relaxation enhancement than on ^6,7^Li, leading to short relaxation times (on the order of the receiver deadtime or the echo evolution period). This results in severely broadened resonances which, in the worst-case scenario, may be unobservable or so broad that they are lost in the baseline.

To mitigate the effect of the hyperfine interaction, low magnetic field strengths are preferred, in contrast to the quadrupolar interaction (see later).

### 4.3 Quadrupolar Interaction

On top of the practical difficulties and large chemical shift ranges, ^17^O NMR spectroscopy is complicated by the strongly quadrupolar nature of ^17^O (nuclear spin *I* = 5/2),^31^ quantified by the quadrupolar coupling constant, *C*_Q_. Even under MAS, a broad spinning sideband manifold is commonly observed—once again making VOCS critical to spectral acquisition—as well as broadening of the central transition (isotropic resonance) and a field-dependent contribution to the chemical shift (known as the quadrupole-induced shift, QIS). For ^17^O, *C*_Q_ varies between hundreds of kHz (high-symmetry O sites) and 10s of MHz for more anisotropic environments (e.g., phosphates, peroxides, and organic systems), the magnitude depending on the covalency or ionicity of the local bonding environment: O sites with more covalent *TM*–O bonds have a larger *C*_Q_ than ionic *TM*–O bonds.^52^

The effect of the quadrupolar interaction may be mitigated by increasing the field strength at which the experiments are performed. However, the Fermi contact interaction and dipolar interactions with the unpaired electrons scale with the field, so performing ^17^O NMR of paramagnetic solids at high fields is not always possible. In practice, “intermediate” field strengths (e.g., 7–11 T) and fast MAS speeds (“fast” compared to the size of the hyperfine and quadrupolar interactions, typically >50 kHz) have been employed to performed ^17^O NMR spectroscopy of LIB cathodes. For additional information regarding the effects of the quadrupolar interaction, please see the SI (section S1).

Finally, we note that both the quadrupolar and hyperfine interactions discussed above contribute to the breadth of the observed resonance. Beyond this, additional broadening arises from a distribution of local environments (due to distributions in local bond angles and lengths, often from disorder); small deviations in the pathways can lead to a change in up to 200 ppm for the bond pathway [Table 1]. In general, peak breadth due to a distribution of local environments dominates over the broadening due to relaxation effects due to the interaction, which in turn exceeds the broadening from quadrupolar interactions. This is not always the case, for example, for materials containing Co^4+^ and Ni^2+/3+^ paramagnetic ions (see the LiNi_0.80_Co_0.15_Al_0.05_]O_2_ and LiCoO_2_ case studies below).

## 5 Calculating ^17^O NMR Parameters

To assist the interpretation of ^17^O NMR, parameters such as the chemical shift, hyperfine shift, and quadrupolar tensors may be calculated.^30,40,43^ Although the observed resonances are generally severely broadened—making fitting from scratch challenging, with many models giving similar quality fits—fitting the spectrum to an initial model guided by *ab initio* calculations is often invaluable in spectrum assignment.

Calculation of these parameters in paramagnetic systems such as LIB and sodium ion battery (NIB) cathodes, however, is not straightforward, owing to the strong electron correlation in 3*dTM* systems. While challenging, however, calculation of quadrupolar and hyperfine NMR parameters of LIB and NIB systems has been successfully completed for a range of materials.^29,30,40^

The layered *TM* oxide materials used as LIB and NIB cathodes generally have many local chemical environments—for example, for an O3 layered cathode material with two *TM* cations occupying the *TM* sublattice in a disordered or partially ordered array, there exist 42 possible local environments for O^2–^ ions (considering nearest- and next-nearest-neighboring *TM* cations only). Calculating the shifts of each of these sites is possible but computationally expensive.

Another approach is to use the bond pathway contribution of a *TM* cation to the nucleus of interest (see above). Here, the Fermi contact shift, δ_FC_, may be decomposed into individual shifts from each *TM* cation, δ_path_:
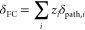
where *z*_*i*_ is the number of pathways of type *i*, with a shift contribution of δ_path*,i*_ (see Table 1 for examples). Appropriate combinations of these pathways may then be compared to the observed spectra to enable assignment.

The quadrupolar parameters may be extracted from the local electric field gradient (EFG) tensors, *V*, in a material (for definitions see SI). Hence, the quadrupolar and hyperfine parameters may be readily computed and compared by fitting to the experimental spectrum [Table 1].

## 6 Applying ^17^O NMR to Studying LIB Cathodes

While it is challenging to acquire and interpret the spectra, ^17^O NMR is rich in information about both the local chemical structure of a material (through its quadrupolar interactions) and the local electronic and magnetic structure (through the hyperfine interaction).^29−33^ In this Perspective, we present a series of published studies on LIB cathodes utilizing ^17^O NMR, alongside a new study on the ^17^O NMR of Li[Ni_0.8_Co_0.15_Al_0.05_]O_2_. Each case study examines a different cathode and aims to use ^17^O NMR to understand the local structure of the pristine material and/or structural evolution during charge/discharge.

## 7 ^17^O NMR as a Tool for Pristine Material Characterization

We first examine how ^17^O NMR may be used to establish the chemical structure and magnetic properties of pristine cathode materials and illustrate the use of bond pathway analysis.

### 7.1 Li_2_MnO_3_

The first use of ^17^O NMR to examine a LIB cathode was in 2016 by Seymour et al., where the local O environments of Li_2_MnO_3_ were explored and the experimental spectra assigned using *ab initio* hybrid DFT calculations of the ^17^O shifts.^40^

**Figure 3:**
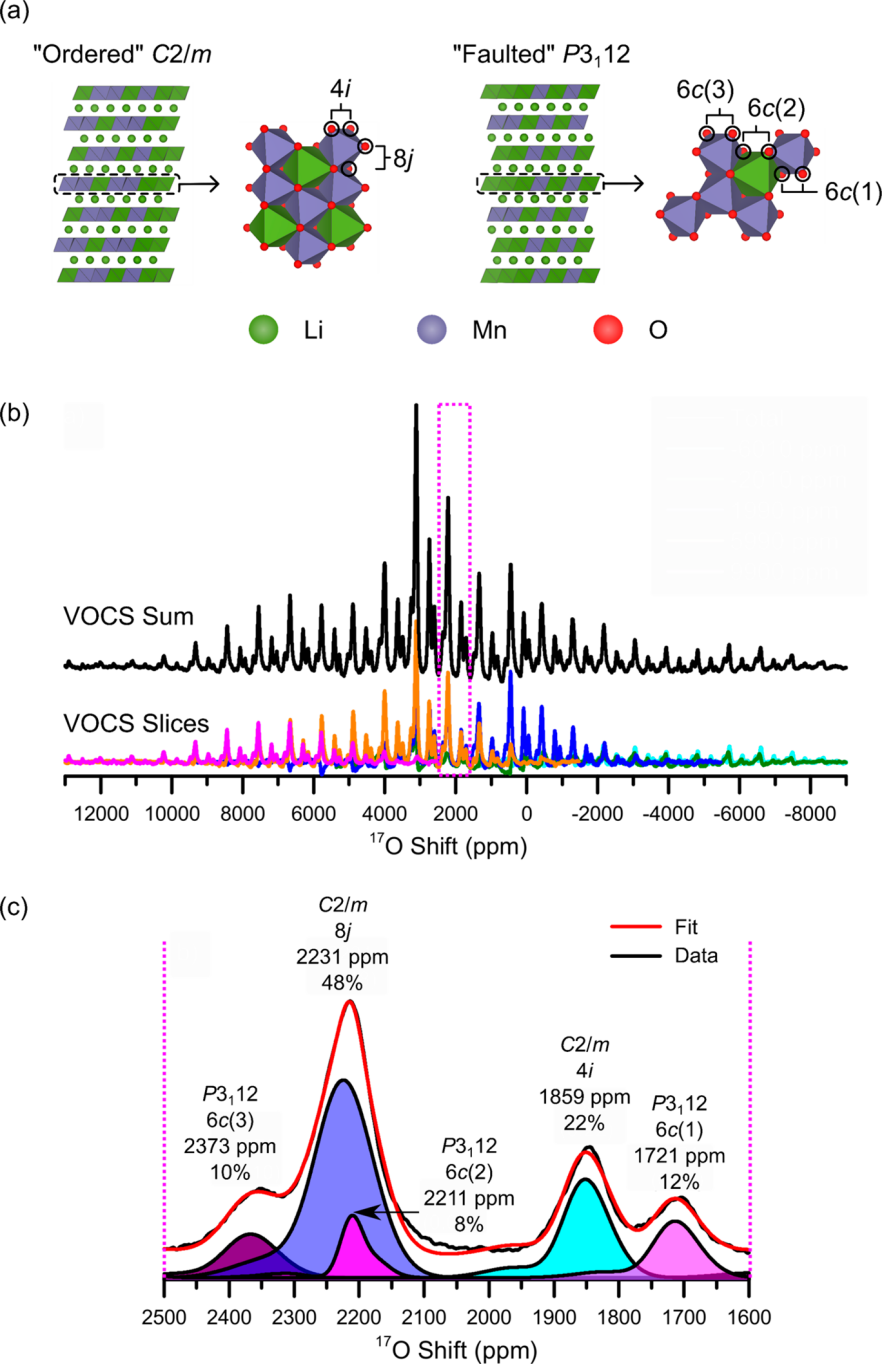
^17^O NMR spectroscopy of Li_2_MnO_3_. (a) Structure of ordered and stacking-faulted Li_2_MnO_3_ with honeycomb ordering in the Li_1/3_Mn_2/3_ layers, but different stacking sequences and symmetries of these layers. Distinct crystallographic O sites are shown and labeled with their Wyckoff positions. (b) ^17^O NMR Hahn-echo VOCS slices and sum for a sample of ^17^O-enriched Li_2_MnO_3_ at 11.7 T and a 60 kHz MAS rate. (c) Expansion of the isotropic region of the spectrum, with peaks from the ordered and faulted phases labeled. Adapted with permission from ref (40). Copyright 2016 American Chemical Society.

Li_2_MnO_3_ adopts a layered structure whose formula can be rewritten as Li[Li_1/3_Mn_2/3_]O_2_, with the Li^+^ and Mn^4+^ cations adopting an ordered honeycomb arrangement [Figure 3a]. This compound is highly susceptible to stacking faults, where the [Li_1/3_Mn_2/3_]O_2_ layers may be offset relative to each other; the “ordered” regions correspond to an O3 structure with space group *C*2/*m*, while the “faulted” regions retain the O3 structure and are locally analogous to the *P*3_1_12 Li_2_MnO_3_ polymorph [Figure 3a]. The extent of stacking faults depends on the synthesis method.^53^

Seymour et al. enriched a sample of Li_2_MnO_3_ using a post-synthetic gas enrichment process and acquired ^17^O Hahn-echo NMR spectra using VOCS to excite the entire spectrum.^40^ By examining spectra at different magnetic field strengths, Seymour et al. were able to identify five isotropic resonances (i.e., five unique local ^17^O environments; Figure 3b,c). These resonances were assigned to specific local environments using bond pathway analysis.

Calculations revealed that the bond pathway shift in both the *C*2/*m* and *P*3_1_12 polymorphs varied with Mn···O distance. In general, however, a single Mn bound directly to O (i.e., the nearest-neighbor Mn to O) gives a shift of +1000 ppm, while Mn indirectly bound to O (i.e., the next-nearest-neighbor Mn to O) contributes approximately +200 ppm. Each unique combination of nearest- and next-nearest-neighbor Mn–O distances (i.e., the unique local environment) results in different shifts for each site.

Based on these bond pathways, two of the observed peaks were assigned to the “ordered” (*C*2/*m*) Li_2_MnO_3_; crystallographically, these are known as the 4*i* and 8*j* sites (Wyckoff labeling), both coordinated to two Mn^4+^ and one Li^+^ cation within the Li^+^ layer, but in different relative positions in the layer. The remaining three were assigned to O environments in the “faulted” (*P*3_1_12) structure, known as 6*c*(1), 6*c*(2), and 6*c*(3) [Figure 3a,c]. By carefully examining the intensities of the resonances, a 1:2 ratio of the 4*i* and 8*j* sites was identified, while the intensity ratios for the stacking fault resonances were approximately 1:1:1, as anticipated from the crystal structure.

This work demonstrated that ^17^O NMR, in conjunction with *ab initio* bond pathway calculations, can identify defect structures and subtle differences (i.e., *TM*···O distances and bond angles) in local environments.

### 7.2 Li_2_RuO_3_

**Figure 4:**
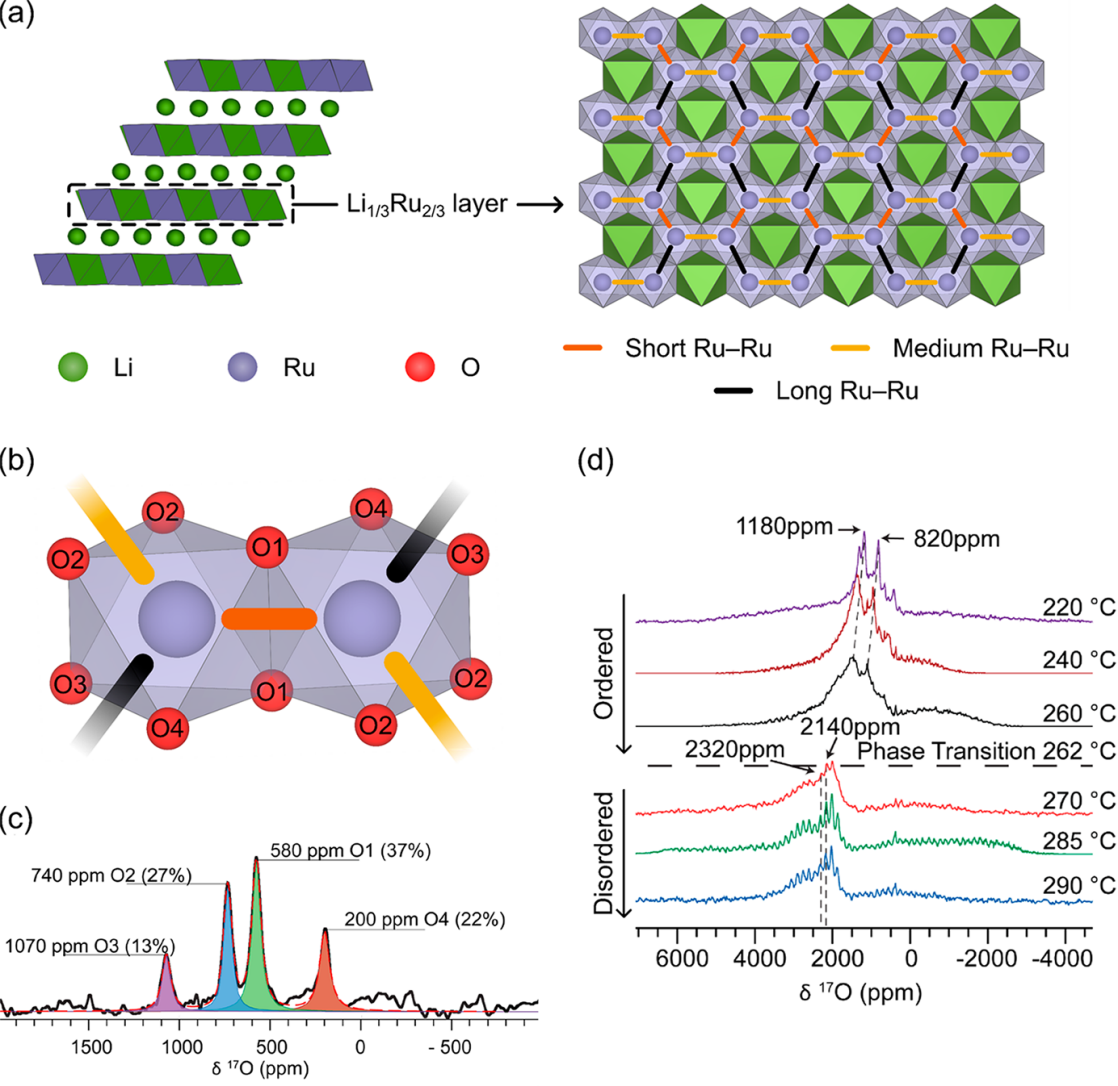
Variable-temperature ^17^O NMR spectroscopy of Li_2_RuO_3_. (a) Structure of Li_2_RuO_3_ (left) with honeycomb ordering in the Li_1/3_Ru_2/3_ layers and ordered arrangement of Ru–Ru dimers (right). (b) Local O environments relative to the Ru–Ru dimers. (c) Room-temperature isotropic ^17^O NMR resonances for ^17^O-enriched Li_2_RuO_3_ at 11.7 T under 60 kHz MAS. (d) Variable-temperature ^17^O NMR Hahn-echo VOCS spectra acquired under 16.4 T and 14 kHz MAS. Adapted with permission from ref (41). Copyright 2019 American Chemical Society.

The next study explored the structure and phase transformation of Li_2_RuO_3_, whose honeycomb-ordered structure with Li^+^ and Ru^4+^ ions in the *TM*O_2_ layer (i.e., [Li_1/3_Ru_2/3_]O_2_, Figure 4), well-characterized Ru^4+/5+^ redox couple, and reversible electrochemistry make this material a good model compound for studying oxygen redox.^41^ The Ru^4+^ cations adopt an ordered array of Ru–Ru dimers with short Ru–Ru distances (generated from direct overlap of the Ru 4*d* orbitals), in addition to long and medium distances [Figure 4a].^54^ The dimerization affects the magnetic susceptibility of the material: at temperatures below 540 K, the magnetic susceptibility is low (due to electron spin pairing), while at high temperatures, a phase transition takes place, where the Ru–Ru bond lengths fluctuate and the dimers are no longer ordered over the structure, resulting in a greater unpaired electron spin density and a higher magnetic susceptibility.^54^

The room-temperature ^17^O NMR Hahn-echo VOCS of Li_2_RuO_3_ comprised four distinct isotropic resonances whose shifts were rationalized based on shifts calculated using DFT and bond pathway analysis and by considering the local orbital interactions between the Ru–Ru dimers and the surrounding ligand field of O^2–^ anions. The four resonances correspond to O^2–^ which is on the inside edge of an Ru–Ru dimer [O1 in Figure 4b]; O^2–^ on the outside edge of an Ru–Ru dimer (O2); O^2–^ between a non-dimerized pair of Ru^4+^ cations (O3); and O^2–^ which is axial relative to an Ru–Ru dimer [O4; Figure 4b,c].

The phase transition was then examined by using variable-temperature ^7^Li and ^17^O NMR (see ref (42) for the ^7^Li NMR spectra). On increasing temperature, the authors observed an increase in the shift of the ^17^O isotropic resonances [Figure 4d]. This is unusual, as one typically expects a decrease in isotropic shift, as, in most materials, the time-averaged electron spin decreases and hence the spin density transferred to nearby nuclei decreases with increasing temperature. In Li_2_RuO_3_, the low-lying paramagnetic states (corresponding to the non-dimerized structure) become accessible at higher temperatures, meaning that the time-averaged spin moment—and therefore the shift—increases.

At temperatures between room temperature and the high-temperature phase transition, two distinct ^17^O resonances were observed and assigned to the two crystallographic O sites in the high-temperature structure, 8*j* (2320 ppm) and 4*i* (2140 ppm), based on a qualitative comparison of the expected spin densities. The 8*j* site sits closer to the Ru cations, while the 4*i* sites sit farther away; this is analogous to the relative shifts of the 8*j* and 4*i* sites in (ordered) Li_2_MnO_3_. The other two resonances seen at room temperature were no longer present, which was ascribed to broadening and greater overlap of the resonances and sidebands, due to a slower MAS speed, as well as faster relaxation times induced by a greater magnetic susceptibility at higher temperatures and hence a stronger hyperfine interaction.

Above the phase transition, the two resonances broadened further and were again assigned to the two crystallographic environments (8*j* and 4*i*) in non-dimerized Li_2_RuO_3_. The authors noted, however, that the non-dimerized model was likely an approximation to a dynamic structure, in which short Ru–Ru distances persist but fluctuate rapidly on the NMR time scale. This rapid fluctuation contributed to the broadening of the resonances and led to a dynamically averaged hyperfine interaction.

This study highlighted how ^17^O NMR may be used as a tool for probing structural phase transformations in layered cathode materials—in terms of both the changes to local environments and the dynamics of these changes.

## 8 ^17^O NMR as a Tool for *Ex Situ* Characterization

We now turn to examining the changes in ^17^O NMR spectra on electrochemical cycling. Since NMR is a non-destructive technique, using only low-energy radiofrequency radiation, it is ideally suited to studying metastable compounds such as cycled cathode materials.

### 8.1 Li[Ni_0.85_Co_0.10_Al_0.05_]O_2_

Li_*x*_Ni_0.8_Co_0.15_Al_0.05_O_2_ (NCA) is a commercial battery cathode used in many EVs, and doping of Co and Al into the parent material, LiNiO_2_, significantly improves its electrochemical performance. To understand the local structural evolution of NCA during cycling, we obtained *ex situ*^17^O, ^27^Al, and ^59^Co NMR spectra. Here, we focus on the ^17^O NMR results and only briefly discuss the ^27^Al and ^59^Co NMR data (see SI for a longer discussion, section S5 and Figures S10–S15).

Pristine NCA contains paramagnetic Ni^3+^ and diamagnetic Co^3+^ and Al^3+^ ions, but with no long-range order of these cations in the *TM*O_2_ layers. Ni^3+^ is Jahn–Teller (JT) distorted—with very different calculated Fermi contact shifts for the shortened and lengthened Ni–O bonds [Table 1]. There is no overall ordering of the JT axes of Ni^3+^, and it has been argued that this is because the JT distortion is dynamic.

**Figure 5:**
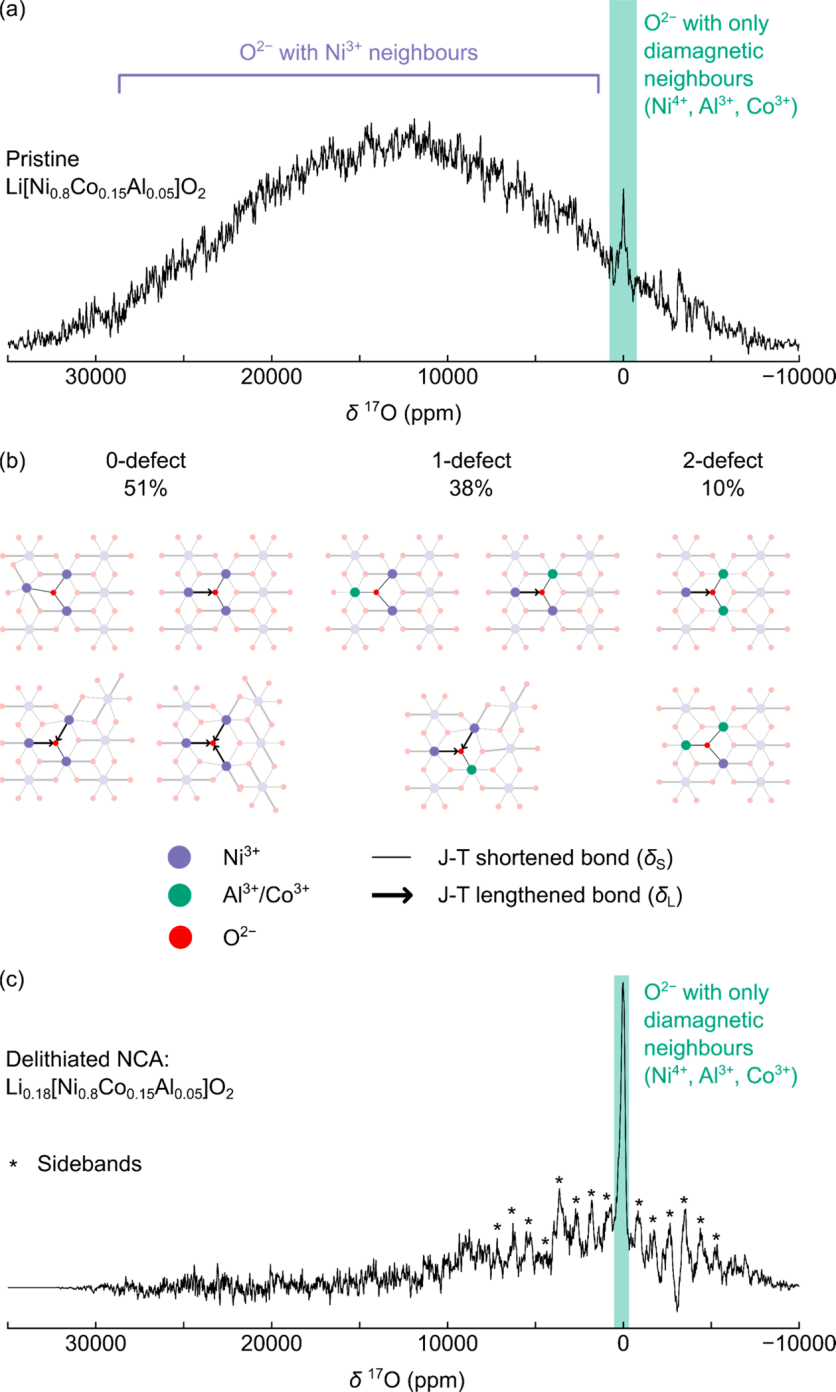
^17^O, ^7^Li, ^27^Al, and ^59^Co NMR spectroscopy of ^17^O-enriched Li[Ni_0.80_Co_0.15_Al_0.05_]O_2_ (NCA). (a) ^17^O NMR VOCS data obtained for pristine NCA at 11.7 T under 60 kHz MAS and at “room temperature” (i.e., the ambient temperature of the rotor due to frictional heating, typically 320 K at 60 kHz). (b) Different paramagnetic local O environments and bond pathway contributions in pristine NCA. (c) Room-temperature spectra obtained for electrochemically charged (i.e., delithiated) NCA, also at 11.7 T under 60 kHz, with composition Li_0.18_[Ni_0.8_Co_0.15_Al_0.05_]O_2_ calculated based on the current passed.

The ^17^O NMR spectrum of pristine NCA is severely broadened (width >30 000 ppm) with a high center-of-mass shift (ca. 13 000 ppm), likely due to a strong hyperfine interaction between the O nuclei and JT-distorted Ni^3+^ centers [Figure 5a].^29^ A sharp feature around 0 ppm is also seen, due to diamagnetic O^2–^ either from surface impurities (e.g., Li_2_CO_3_, LiOH) or from O^2–^ surrounded by diamagnetic (Al^3+^ and Co^3+^) centers in the transition metal layer. This diamagnetic feature is also consistent with a γ-LiAlO_2_ impurity phase seen in the ^27^Al NMR (Figure S10).

Bond pathway analysis was used to assign the broad resonance, assuming a random *TM* distribution model, where any *TM* site can be occupied by Ni^3+^, Co^3+^, or Al^3+^, with occupation probabilities given by the NCA stoichiometry. We define three paramagnetic environments, categorized according to the number of nearest-neighbor Al^3+^ or Co^3+^ (dopant) centers—a 0-dopant site, where O is bound to three Ni^3+^ nearest neighbors; 1-dopant, where O has two Ni^3+^ and one Al^3+^ or Co^3+^ nearest neighbor; and 2-dopant, with one Ni^3+^ and two diamagnetic neighbors, Al^3+^ and/or Co^3+^—with relative concentrations of 51%, 38%, and 10%, respectively. The remaining sites (ca. 1%) are diamagnetic, with no Ni^3+^ neighbors. Additional complexity arises as a single Ni^3+^ neighbor can be coordinated to O via a JT-lengthened bond (whose bond pathway contribution is δ_L_, approximately 12 500 ppm) or a JT-shortened bond (bond pathway shift δ_S_, approximately 2300 ppm). These bond pathway contributions were taken from previous hybrid DFT calculations of Al-doped LiNiO_2_.^27^

For the 0-defect site, there are four “sub-environments”, due to different configurations of long and short JT-distorted Ni–O bonds. O can be bound to Ni^3+^ via three δ_L_ paths; two δ_L_ paths and one δ_S_ path; one δ_L_ path and two δ_S_ paths; or three δ_S_ paths [Figure 5b]. Under a dynamic JT distortion model—known to model the ^7^Li NMR spectra—the shift of the 0-defect site will be the thermodynamic average of these sub-environments. We anticipate that the environment with one δ_L_ path and two δ_S_ paths (total shift of 17 100 ppm) will be the lowest in energy (all others will be more strained and therefore higher energy). As a result, the shift of O with three Ni^3+^ nearest neighbors in a dynamic JT network will be close to the 17 100 ppm. The ^17^O NMR results are, therefore, consistent with a dynamic JT distortion.

The 1-dopant site comprises three local environments for a static JT, with total shifts of 25 000 ppm (2δ_L_), 14 800 ppm (δ_L_ + δ_S_), and 4600 ppm (2δ_S_). Previous publications suggested that the dopants may pin the JT distortion so that the NiO_6_ long axis points toward the smaller Al^3+^ dopant. If all Ni^3+^ JT ions are dynamic, then a broad resonance is seen whose center-of-mass is an average of the above shifts results. This shift is likely weighted toward the low-strain δ_L_ + δ_S_ and 2δ_S_ environments (analogous to the 0-defect sites; Figure 5b).

Finally, for the 2-dopant sites, the shifts due to Ni^3+^ are either 12 500 ppm (δ_L_) or 2300 ppm [δ_S_; Figure 5b]. In the case of dynamic JT averaging, we expect a shift of 5700 ppm (i.e., (δ_L_ + 2δ_S_)/3).

Independent of the degree of dynamics for the JT, a wide range of resonances are generated, and therefore the breadth of the observed resonances for pristine NCA likely arises from the overlap of several resonances with very different shifts. We must also consider the rapid fluctuation of the electron spin moment due to the dynamic JT axes. If the rate of these fluctuations (i.e., 1/*t*_JT_, where *t*_JT_ is the time scale of a single JT fluctuation) is close to the frequency difference between the signals from different JT orientations, then significant line broadening will occur.^55^ The overlap of several of these individual broad resonances and their sideband manifolds results in a severely broadened spectrum.

The loss of paramagnetic Ni^3+^ centers on charging NCA is evident from ^17^O NMR: a simultaneous decrease in intensity near the paramagnetic region (13 000 ppm, as seen in the pristine material) and increase in intensity near the diamagnetic region (near 0 ppm) is seen, resulting in a much lower center-of-mass shift, around 600 ppm [Figure 5c]. Despite possible oxidation of diamagnetic Co^3+^ to paramagnetic Co^4+^ (note that some Li remains in the structure at the end of charge), little effect is seen in the ^17^O (or ^7^Li and ^27^Al) NMR, likely due to the small bond pathway shifts for Co^4+^ (Co^4+^ has only one unpaired electron). Furthermore, Co^4+^ induces rapid relaxation, and any resonances arising from environments with pathways to Co^4+^ may be severely broadened, analogous to the disappearance of the ^7^Li NMR signal in LiCoO_2_ on charging.^56^ As low-spin Co^4+^ is a *t*_2g_^5^ ion, its degenerate ground state has residual orbital angular momentum, potentially introducing further broadening mechanisms (see SI).

The ^17^O NMR results for electrochemically charged NCA are therefore consistent with oxidation of Ni^3+^ to Ni^4+^; further evidence for this charge compensation scheme can be seen in the ^59^Co NMR spectra (SI, Figures S14 and S15). Intriguingly, NCA at the end of charge has been shown to exhibit a feature in the O *K*-edge resonant inelastic X-ray scattering (RIXS) spectrum at a certain energy.^18^ Some claim this as characteristic of highly covalent *TM*–O bonds and the removal of electron density from both the *TM* and O ions, while formally oxidizing the *TM* ion;^18^ others view this feature—albeit in different materials—as a signature for anion redox processes.^57−60^ No clear evidence for O oxidation is seen via the ^27^Al or ^59^Co NMR spectroscopy, and the ^17^O NMR spectra of NCA do not appear to contain any signals that can be assigned to either paramagnetic O species or any (O_2_)^*n*−^ dimers, though the question remains: If paramagnetic O species exist, either holes or dimers, would they be observed? We discuss this further in the next section and provide a more detailed discussion of the results and charge compensation mechanisms in NCA in the SI (section S5). What is clear, however, is that acquisition of ^17^O from Ni^3+^-containing disordered samples is extremely challenging, as is the quantification of the ^17^O spectra with the accuracy required to rule out minor species. Thus, care must be taken in using ^17^O NMR to make definitive statements in the absence of other complementary characterization tools when disorder and paramagnetic Ni ions are present.

### 8.2 LiCoO_2_

**Figure 6:**
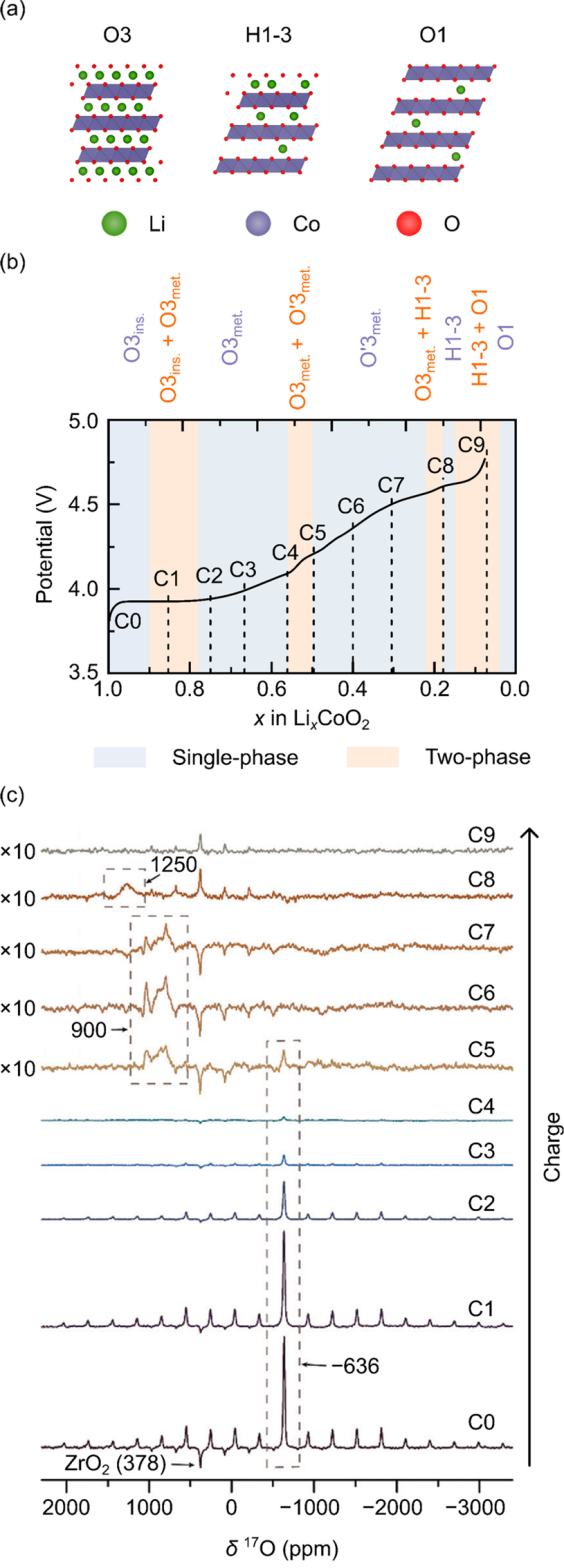
*Ex situ*^17^O NMR results for Li_*x*_CoO_2_ (1.00 < *x* < 0.07) during the first charge. (a) Structures of the O3, H1-3, and O1 phases of Li_*x*_CoO_2_. (b) Corresponding voltage profile and locations of *ex situ* sample points. (c) ^17^O NMR spectra collected at 14.1 T under 24 kHz MAS. Adapted with permission from ref (38). Copyright 2019 Royal Society of Chemistry.

Lithium cobalt oxide, LiCoO_2_ (LCO), has dominated the market as a cathode for LIBs in portable electronics for years.^61^ Pristine LCO adopts a layered O3 structure [Figure 6a], and its structural evolution during cycling is well-characterized [Figure 6b].^62−64^ The pristine phase can be partially delithiated before transforming via a two-phase reaction with a large immiscibility gap to a metallic phase O3_met_. O3_met_ can be further delithated eventually forming another metallic phase, O′3_met_ (with composition close to Li_0.5_CoO_2_), with an ordered array of Li^+^ ions and vacancies. This ordered phase has monoclinic symmetry and only persists over only a very small composition range. The symmetry of the O′3_met_ phase, when further delithiated, returns to hexagonal (as the Li-ordering is lost) and eventually transforms into the H1-3 phase; further delithiation results in a two-phase reaction between H1-3 and the O1 phase, which persists to the end of charge [Figure 6a,b].

Commercially, LCO is generally only charged up to the O′3_met_ structure, corresponding to 0.5 equiv of Li^+^ removed, as the (high-voltage) phase transformations are destructive, resulting in degradation of LCO and the electrolyte.^65,66^ We note that materials that can withstand cycling to 4.3 V vs Li have more recently been developed.^67,68^

To understand these destructive phase transformations, Geng et al. studied LCO using *ex situ*^17^O NMR for the first time, alongside ^7^Li and ^59^Co NMR.^38^ They prepared samples using ^17^O-enriched H_2_O—highlighting that this synthetic route may be successfully employed for layered cathode materials—and carried out post-mortem analysis on a series of cells cycled to different states of charge [Figure 6]. Single-pulse experiments were used, as the authors observed that the Hahn echo resulted in a loss of signal intensity during acquisition, due to rapid transverse relaxation times, *T*_2_. Additional experiments were carried out at a different receiver offset frequency. Note that a resonance with negative intensity appears at 378 ppm, corresponding to ZrO_2_, for reasons discussed in the SI (section S4).

The pristine LCO spectrum comprised a single, sharp resonance at −636 ppm from O^2–^ centers bound to diamagnetic Co^3+^; this resonance is sharper than in paramagnetic LIB cathodes,^37,39−41^ as there is no hyperfine interaction between O and the nearby diamagnetic Co^3+^. On delithiating LCO up to point C5 (i.e., the O′3_met_ phase up to Li_0.5_CoO_2_), the resonance at −636 ppm broadened and decreased in intensity; this was ascribed to a faster relaxation rate of ^17^O due to the formation of paramagnetic Co^4+^ centers. The loss of intensity of this resonance is, however, unsurprising, as the delithiation reaction occurs via a two-phase reaction to form a second distinct phase, O3_met_.

By correlating the ^17^O results with changes in the ^7^Li and ^59^Co NMR spectra, as well as the electrochemical profile and expected phases, the authors concluded that the ^17^O signal from the metallic phase O3_met_ could not be observed. They assigned the resonance at 900 ppm to O^2–^ in the Li^+^/vacancy ordered O′3_met_ phase and the 1250 ppm resonance to O^2–^ in the H1-3 phase.

One difficulty in interpreting these results is the lag between the appearance and disappearance of the different ^17^O resonances and the state of charge (as measured electrochemically), which the authors ascribed to their ^17^O labeling procedure: the characteristic ^7^Li signals were observed for each phase/stage, albeit at a different (nominal) Li^+^ content than expected. The authors ascribed the difficulty in observing the ^17^O resonance from the O3_met_ phase to *t*_2g_ electron delocalization (no signal was observed via ^59^Co NMR, either). They argued that this was evidence for high spin density near these nuclei, the large hyperfine interactions causing rapid relaxation of the NMR nuclei. Despite this, the ^7^Li signals are still observable, the observed shifts being ascribed to a transferred hyperfine interaction to Li nuclei via O^2–^. This phase is, however, generally considered to be metallic—as per conductivity measurements^69^—with the Li shift controlled by the Knight shift. However, the ^17^O results suggest that a simple metallic picture may not be appropriate and that some “intermediate” spin character may be needed to describe the Co ions; a more detailed variable-temperature NMR study is required to understand the nature of defects in pristine and cycled LCO, to reconcile the ^17^O and ^7^Li NMR shift mechanism, and to establish the role that hyperfine (with localized electron spin density) vs Knight shifts play in the observed spectra.

In addition to monitoring the different environments generated during charge, the authors extracted information about the quadrupolar interaction of each O environment. Good fits to the observed spectrum were obtained with a quadrupolar model, where the *C*_Q_ was observed to increase from 1.47 MHz in pristine LCO to 7.22 and 7.98 MHz at higher states of charge. This large increase was ascribed to increasing covalency of the Co–O bond,^52^ due to the lowering in energy and contraction of the Co 3*d* orbitals (resulting in improved spatial and energetic overlap with the O 2*p* orbitals). Further studies to investigate the field dependences of the quadrupolar broadening would be useful to explore this change in *C*_Q_, especially since the changes in the degree of local ionicity and covalency of O have important ramifications for the charge compensation mechanism in layered LIB cathodes.^70^

## 9 High-Capacity Cathodes and ^17^O NMR: Oxidized Oxygen Species

We now examine the use of ^17^O NMR spectroscopy to study materials that nominally operate via anion redox processes. Oxidation of O is not necessarily surprising, given that oxidation of sulfur-based cathodes is well-known^71−74^ and that O oxidation readily occurs in biological settings.^75^ However, the nature of oxidized O (i.e., how these species are stabilized) remains under fierce debate and appears to be material-dependent. Broadly, the nature of oxidized oxygen may be classified in one of two groups: (1) superoxo/peroxo-like species and/or trapped O_2_ units;^39,70,76−80^ (2) rehybridization, delocalization, and changes to ionicity and covalency of the *TM*–O bonds and Li–O–Li units.^81−84^ Both schemes indicate that O participates in redox couples of layered cathode materials, with the extent of its involvement depending on the degree of local ionicity or covalency of O. An explanation of the most common mechanisms (formation of peroxo-like (O_2_)^*n*−^ species;^78−80^ molecular oxygen trapping;^39,77,85^ localization of holes onto O;^84,86,87^*TM* migration;^88−90^ and π redox^81^) is beyond the scope of this Perspective, but a brief summary may be found in the SI, section S3 and Figures S4–S8.^91^

The most common method used to understand charge compensation mechanisms in O redox cathodes is O *K*-edge XAS.^39,85,92−95^ In particular, advanced XAS techniques such as RIXS and hard X-ray photoelectron spectroscopy (HAXPES) are becoming increasingly common for examining the highest occupied and lowest unoccupied electronic states around O.^92,94^ While XAS can provide an element-specific local handle on the oxidation state and electronic and chemical structures of a material, the high-energy X-rays used in these techniques can damage the sample.^93^ Some studies have also chosen to incorporate XRD,^88,89,96^*ab initio* electronic structure calculations,^81,82,97^ electron paramagnetic resonance (EPR) spectroscopy,^37,98^ and ^6^Li and ^7^Li NMR.^37,39,85^ Thus far, however, only two have used ^17^O NMR spectroscopy.^37,39^ We now examine why the application of ^17^O in these cases has been so challenging and discuss what information can be obtained.

### 9.1 Li_1.2_Ti_0.4_Mn_0.4_O_2_ (LTMO)

**Figure 7:**
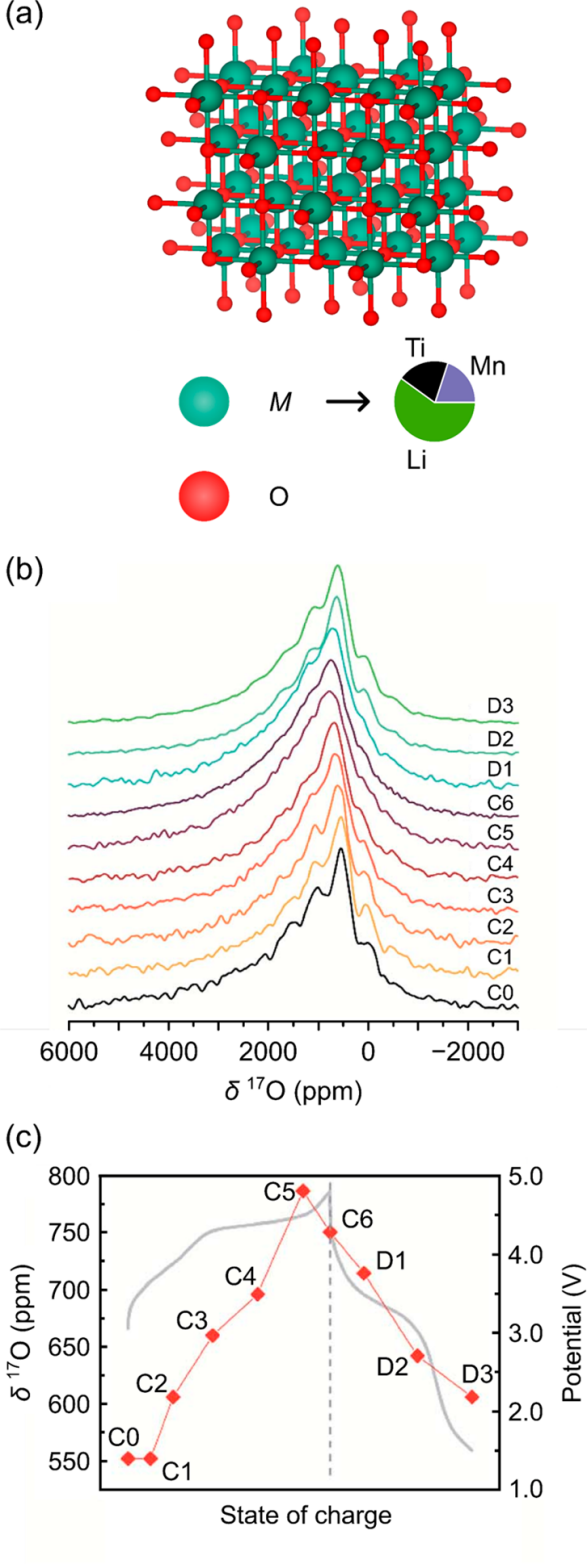
^17^O NMR spectroscopy of the ^17^O-enriched disordered rocksalt Li_1.2_Ti_0.4_Mn_0.4_O_2_. (a) Structure of Li_1.2_Ti_0.4_Mn_0.4_O_2_, with a “random” distribution of the metal cations (Li^+^, Mn^3+^, and Ti^4+^). (b) *Ex situ* spectra recorded at 18.8 T under 60 kHz MAS, with the states of charge on the voltage curve in (c). Adapted with permission from ref (37). Copyright 2020 Royal Society of Chemistry.

The first study to examine an O-redox-active cathode using ^17^O NMR was carried out by Geng et al. on Li_1.2_Ti_0.4_Mn_0.4_O_2_ (henceforth LTMO), a disordered rocksalt.^37^ Disordered rocksalts are a class of cathode materials which are distinct from (but related to) layered cathodes. They comprise two interpenetrating face-centered-cubic lattices of octahedrally coordinated ions: one of O^2–^ anions and the other with a disordered (or partially ordered^99^) array of cations [Li^+^ and *TM*; Figure 7a]. Some disordered rocksalts may be made lithium-rich, where the Li:*TM* ratio exceeds 1 and Li^+^ cations replace *TM* centers; here, it is hypothesized that the highly ionic Li–O bonds raise the energy of the O non-bonding lone pairs, making these electrons available for reversible redox reactions.^7,8,100−103^

To examine the charge compensation scheme, Geng et al. sought to identify the local structural changes through ^17^O NMR and, as discussed in their paper, ^7^Li NMR and EPR.^37^ The ^17^O NMR spectra for LTMO were acquired using a solid echo pulse sequence; the difference between this and the Hahn-echo and single-pulse experiments used in the earlier studies is detailed in the SI (section S4, p S-13).

Pristine LTMO has a ^17^O NMR spectrum which is severely broadened, with sidebands that overlap significantly with the most dominant and most intense isotropic resonance at approximately 550 ppm. The 550 ppm peak was assigned to O^2–^ bound to Mn^3+^ centers [Figure 7b] and was confirmed as the isotropic resonance by acquiring spectra at different MAS speeds and field strengths. Given the shifts seen in the related layered materials where the paramagnetic ions have been found to cause large hyperfine shifts, it seems unlikely that this assignment is correct. Since the spectra in this work are not VOCS, we anticipate that only a small portion of the spectrum was recorded, and that additional isotropic resonances may lie at higher frequencies—even in the spectrum presented, a broader signal (which may not have been fully excited under the acquisition conditions) sitting underneath, but at higher frequencies than the 550 ppm resonance, is likely present.

The severe broadening of the observed spectrum was assigned to a broad distribution of local environments, a consequence of the disordered nature of the material, as well as the presence of strongly paramagnetic Mn^3+^ cations, resulting in rapid nuclear relaxation rates.

On charging LTMO, the ^17^O isotropic shift moved to slightly higher frequencies (ca. 780 ppm). The shift decreased on discharge but did not return to the same shift as the pristine material, reflecting the hysteretic behavior seen over the first charge–discharge cycle [Figure 7b,c]. It was suggested that the small increase in shift is due to the similar Fermi contact shifts induced by Mn^4+^ and (O_2_)^*n*−^ (peroxo-like) species. The authors also noted a broader sideband manifold in the ^17^O NMR spectrum on charging and ascribed it to a stronger quadrupolar interaction (i.e., a larger *C*_Q_ and therefore a more covalent *TM*–O bond) and/or a broader distribution in local environments.

Given that O bound to Ti typically resonates near 500 ppm^31,104^ and that the probability of not having a paramagnetic Mn in the first coordination sphere, but instead having a configuration OTi_*x*_Li_6–*x*_ (where 0 < *x* ≤ 6), constitutes a significant fraction of the oxygen sites (26%), we reassign the 550 ppm resonance to O bound to Ti^4+^ rather than Mn^3+^ centers. The broad shoulder to higher frequencies is then due to configurations with Mn^3+^ ions in the first coordination shell. At C3, all Mn^3+^ should have been oxidized to Mn^4+^, based on the typical bond pathways expected for Mn^4+^ bound to O—i.e., 900–1100 ppm for a nearest neighbor and 200 ppm for a next-nearest neighbor, based on the bond pathways in Li_2_MnO_3_—and given that no new resonances are observed at this state of charge, we expect that any environments in which O is surrounded by Mn are at higher frequencies and likely buried in the broad shoulder.

The typical ^17^O shift range of diamagnetic peroxide species is 200–800 ppm,^43^ suggesting that the assignment of some of the observed signal as (O_2_)^*n*−^ is at least plausible. However, it is well-known that peroxide species have large *C*_Q_ values^105^ (up to 20 MHz for Li_2_O_2_), suggesting that this assignment would need to be verified via calculations of the C_*Q*_ and shift parameters. It appears likely that distortions in the O sublattice are seen as the Li^+^ ions are removed, resulting in broadening of the O signals, and it is more likely that these species are, in fact, lattice oxygen.

More importantly, if there are unpaired electrons on O (i.e., if *n* in (O_2_)^*n*−^ deviates from 2), it seems highly unlikely that the ^17^O signals could be seen using NMR: the rapid relaxation induced by the extremely strong hyperfine interactions would likely cause signal decay within the dead time, resulting in severely broadened features and/or a significant loss of signal intensity.

### 9.2 Li_1.2_Ni_0.13_Co_0.13_Mn_0.54_O_2_ (LR-NMC)

**Figure 8:**
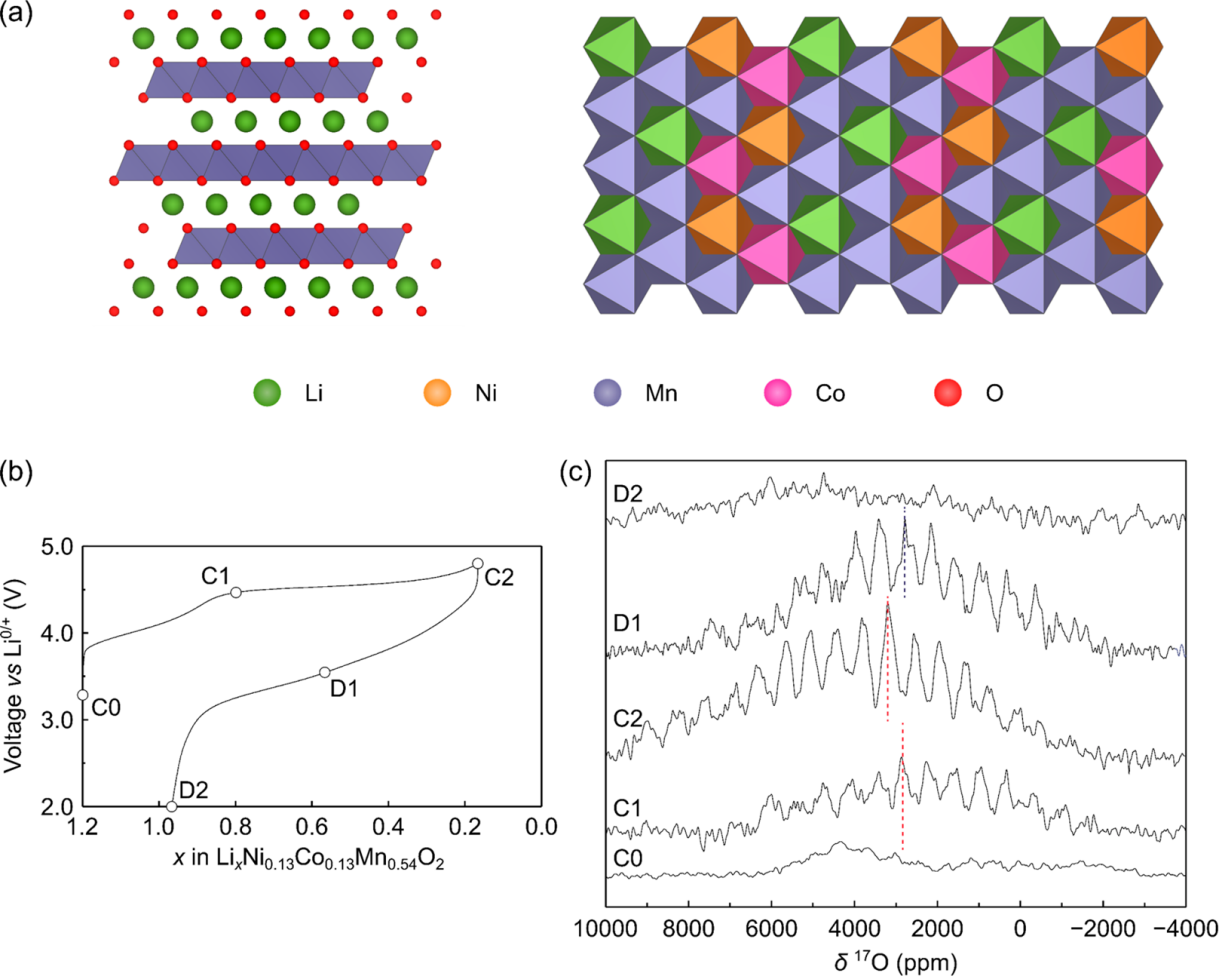
^17^O NMR spectroscopy of the ^17^O-enriched lithium-rich cathode Li_1.2_Ni_0.13_Co_0.13_Mn_0.54_O_2_. (a) Structure of Li_1.2_Ni_0.13_Co_0.13_Mn_0.54_O_2_, with a honeycomb arrangement of the Li^+^ and TM cations. (b) *Ex situ* sampling points on the voltage curve, with the corresponding spectra shown in (c), recorded at 9.45 T under 34 kHz MAS. Adapted with permission from ref (39). Copyright 2020 Nature-Springer group.

The second (published) study to have explored the charge compensation mechanism of high-capacity cathode materials using ^17^O NMR (among other techniques) was from House et al., who studied a lithium-rich nickel–manganese–cobaltate (NMC) material.^39^ As in lithium-rich rocksalts, Li^+^ ions in lithium-rich layered cathodes substitute some of the *TM* cations in the *TM*O_2_ layers [Figure 8a]. In this work, House et al. studied Li_1.2_Ni_0.13_Co_0.13_Mn_0.54_O_2_ (henceforth LR-NMC) to understand the charge compensation scheme during the first charge–discharge cycle [Figure 8b].

On the basis of O *K*-edge RIXS data obtained at the end of charge, the authors proposed that charge compensation in LR-NMC proceeded via the oxidation of the lattice oxygen, which is subsequently stabilized by pairing, *TM* migration, and the formation of trapped O_2_. To complement their RIXS data, they acquired *ex situ*^17^O NMR spectra using a short-delay pre-saturation echo pulse sequence (as illustrated and discussed in the SI, Figure S9). Briefly, the pre-saturation sequence enables only species with short longitudinal relaxation times, *T*_1_, to be observed. In theory, if O becomes strongly paramagnetic and relaxes quickly, it would be more clearly separated from the diamagnetic species than if longer delays were used.

The ^17^O NMR spectrum of pristine LR-NMC showed a severely broadened signal, which, while not explicitly assigned or fitted by the authors, likely arises from overlapping resonances which correspond to O^2–^ ligands bound to varying numbers of Mn, Ni, and Co cations;^39^ each of these resonances will likely have a rapid transverse relaxation time (*T*_2_), due to the strongly paramagnetic nature of the *TM* cations,^45^ resulting in a broad resonance [Figure 8c], consistent with the ^17^O spectrum obtained for NCA [Figure 7].

On charging, a (comparatively) sharp resonance develops at approximately 2800 ppm, which becomes more intense and moves to approximately 3200 ppm at the end of the first charge [Figure 8c]. This resonance was assigned to trapped molecular O_2_, based on a prior study that reported the ^17^O NMR spectrum of condensed (liquid) ^17^O_2_ at 77 K. While the experimental magnet strengths and temperatures differ between the two studies, a Curie–Weiss scaling may be used to translate between the shifts.^46^ Assuming a Weiss constant of −71.3 K (from ref (106)), and converting from the reported shift of 8220 G for liquid O_2_, we obtain a shift of approximately 10 980 ppm for O_2_ at 310 K (the temperature expected at this MAS frequency; see SI, section S7, for details on conversion calculations), which is significantly different from the observed resonance reported by House et al.^39^

The shift of the observed resonance is, however, comparable to those seen for ^17^O_2_ environments in isolated haem complexes (whose shifts lie around 1600–2000 ppm).^107^ These complexes contain strongly coupled *TM*–O_2_ interactions, such that the Fe–O_2_ complex is diamagnetic, with the ^17^O_2_ changing very little between 298 and 77 K. Thus, if this is indeed “O_2_”, then it cannot be free triplet (*S* = 1) oxygen, and it must interact strongly with the metal sublattice. Such O_2_ species would also likely have large *C*_Q_ values (comparable to peroxides, up to 5 MHz^107^); this quantity can be readily obtained via DFT.

Based on the shift and rapidly relaxing nature of the resonance seen by House et al.,^39^ it is also possible that this resonance corresponds to O environments bound to strongly paramagnetic Mn^4+^ and/or Co^4+^ cations (note that Ni^4+^ is expected to be diamagnetic). Furthermore, as previously shown for the Li_2_MnO_3_ system, O sites connected to two Mn^4+^ are expected to have shifts around 2000 pm, suggesting that these new features are simply O bound to Mn with longer ^17^O T_1,2_ relaxation times (giving rise to sharper resonances), and which become visible due to the loss of paramagnetic Ni^2+^ and Ni^3+^ centers.

The study by House et al. highlights the importance of using several techniques—both theoretical and experimental—to probe the changes to electronic and chemical structure so as to verify the assignments.

### 9.3 Li_2_MnO_3_

The most recent study of the O redox mechanism using ^17^O NMR examined Li_2_MnO_3_ both *ex situ* and *in situ*.^108^ On cycling, the reversible capacity extracted from this material is, in principle, charge compensated by O redox only, as Mn is already in the +4 oxidation state. In this study, Li et al. synthesized ^17^O-enriched Li_2_MnO_3_*as per* the method used by Seymour et al.^40^ and carried out pjMATPASS experiments on *ex situ* cycled samples. Importantly, the authors showed that *in situ* (static) ^17^O NMR was possible through use of the quadrupolar Carr–Purcell–Meiboom–Gill (qCPMG) to increase the sensitivity and temporal resolution of spectra.^109,110^ A gradual loss in the intensity of all O peaks on charging was seen, where the losses in intensity were greatest for ^17^O environments from the stacking-faulted (*P*3_1_12) domains. This was ascribed to formation of paramagnetic O species, predominantly stabilized via Mn–O π redox (see SI, sections S3 and S9 and Figure S17). Additional signals were observed, which we suggest arise from degradation products, most likely facilitated by proton insertion and densification at the surface of Li_2_MnO_3_ particles.^111−113^ This work highlights the utility of ^17^O in analyzing local structures during operation of a battery—i.e., without the structural relaxation that may occur in *ex situ* samples after cycling.

## 10 Outlook and Conclusions

^17^O NMR spectroscopy is an invaluable technique for determining the local structures of LIB—and indeed other—cathode materials. It is capable of reporting the changes to chemical structure, phase transformations, and electronic structure during charge and discharge.^40,41^

In the pristine material, the O starts as an oxide ion and is generally bound to at least one highly paramagnetic *TM* center, resulting in a strong hyperfine interaction, large shifts, and rapid nuclear relaxation. How well-defined or broadened a resonance is depends primarily on the strength of the hyperfine interaction, the ^17^O nuclear relaxation times, and the distribution of local environments. The quadrupolar interaction also contributes to the observed breadth, but generally to a much smaller extent.

Spectra with well-defined resonances are anticipated for ordered compounds (e.g., Li_2_RuO_3_), systems where the O species are not bound to a dynamic Jahn–Teller distorted paramagnetic center (i.e., not bound to Mn^3+^ or Ni^3+^), where O has distinct chemical environments, and/or where little or no spin–orbit coupling is present. Where resolved signals are seen, e.g., in Mn^4+^-containing compounds such as Li_2_MnO_3_, disorder can be probed—for example, stacking faults which generate discrete local environments with distinct (and calculable) hyperfine shifts. For systems containing Ni^2+/3+^, Mn^3+^, and Co^4+^, significant broadening of the resonances results, likely from JT distortions and/or non-zero orbital angular momentum, *L*, particularly when fluctuations (such as the dynamic JT effect) are present on the time scale of the hyperfine interaction. Magnetic interactions between paramagnetic ions, or spin pairing for paramagnetic systems that undergo metal–insulator transitions, will also result in broadening as the magnetic or metallic phase, respectively, is approached. Additional work is required to understand these broadening mechanisms, perhaps using EPR and/or magnetic susceptibility studies.

Hybrid DFT calculations of the expected shifts not only assist the assignment and interpretation of a spectrum but also aid in the initial search for a resonance. However, care must be taken when making assignments: one must ask whether the observed resonance(s) are from diamagnetic surface species (and thus easier to see, e.g., Li_2_CO_3_, Li_2_O) or from the bulk, and indeed if the full spectrum has been excited (i.e., whether VOCS is necessary). Are the spectra quantitative, and are all the signals from the whole sample being seen, or are some signals lost via some of the mechanisms discussed above?

Further information may be gleaned by extracting the quadrupolar parameters, since these report the degree of ionicity and covalency. To properly assess and extract these quadrupolar parameters, further experiments are often required, be it measurements at different field strengths, nutation profiles (by acquiring spectra with different pulse lengths), or the use of quadrupolar-filtering pulse sequences.^29^ Variable-temperature experiments are also important to help tease apart the various interactions—be they diamagnetic, Fermi-contact, or Knight shift—and to explore the nature of magnetic interactions in these systems.

Having established the nature of O environments in the pristine material, the consequences of cycling can be explored using similar approaches. The width of the observed resonances and the short nuclear relaxation times make it extremely challenging to assign spectra to specific O environments, be they O^2–^ anions or more oxidized oxygen species.

Moving forward, ^17^O NMR should be a useful tool for exploring the inherently challenging O redox chemistry, as it provides a non-destructive method of examining the local chemical environment around O. From the studies presented in this Perspective, it is clear that further work is required to fully understand how paramagnetic O centers (either as holes on O, delocalized spin states, or (O_2_)^*n*−^-like species) manifest in the ^17^O NMR spectrum of cathodes at the end of charge.

As new materials are discovered, it may be possible to find systems containing fewer paramagnetic ions and local environments, to simplify the analysis. Dynamic nuclear polarization (DNP) using endogenous radicals may enhance the ^17^O signals and select for nearby environments,^114^ and, again, with a judicious choice of system with only dilute paramagnetic ions, low-concentration O defects may become visible. Care must also be taken that electrolyte reactions do not complicate the analysis, particularly at high states of charge where proton insertion is common.

It is also advisable to obtain additional data, for example, magnetometry to understand the number of and interactions between unpaired electrons and EPR to probe the local environment of these unpaired electrons. When combined, a truly holistic picture of both the charge compensation mechanism and the structural evolution of a cathode during cycling may be obtained, and controversial questions regarding the nature of oxygen’s involvement in the electrochemistry of this class of materials may be answered.

## Supporting Information Available

Discussion of the O redox charge compensation schemes, results, and subsequent discussion of NMR data acquired for NCA (PDF)

## References

[ref1] Ma J.; Li Y.; Grundish N. S.; Goodenough J. B.; Chen Y.; Guo L.; Peng Z.; Qi X.; Yang F.; Qie L.; Wang C.-A.; Huang B.; Huang Z.; Chen L.; Su D.; Wang G.; Peng X.; Chen Z.; Yang J.; He S.; Zhang X.; Yu H.; Fu C.; Jiang M.; Deng W.; Sun C.-F.; Pan Q.; Tang Y.; Li X.; Ji X.; Wan F.; Niu Z.; Lian F.; Wang C.; Wallace G. G.; Fan M.; Meng Q.; Xin S.; Guo Y.-G.; Wan L.-J. The 2021 Battery Technology Roadmap. J. Phys. Appl. Phys. 2021, 54 (18), 183001 10.1088/1361-6463/abd353.

[ref2] Tarascon J.-M.; Armand M. Issues and Challenges Facing Rechargeable Lithium Batteries. Nature 2001, 414 (6861), 359–367. 10.1038/35104644. 11713543

[ref3] Blomgren G. E. The Development and Future of Lithium Ion Batteries. J. Electrochem. Soc. 2017, 164 (1), A5019 10.1149/2.0251701jes.

[ref4] Schmuch R.; Wagner R.; Hörpel G.; Placke T.; Winter M. Performance and Cost of Materials for Lithium-Based Rechargeable Automotive Batteries. Nat. Energy 2018, 3 (4), 267–278. 10.1038/s41560-018-0107-2.

[ref5] Manthiram A. A Reflection on Lithium-Ion Battery Cathode Chemistry. Nat. Commun. 2020, 11 (1), 1–9. 10.1038/s41467-020-15355-0. 32214093 PMC7096394

[ref6] Radin M. D.; Hy S.; Sina M.; Fang C.; Liu H.; Vinckeviciute J.; Zhang M.; Whittingham M. S.; Meng Y. S.; Van Der Ven A. Narrowing the Gap between Theoretical and Practical Capacities in Li-Ion Layered Oxide Cathode Materials. Adv. Energy Mater. 2017, 7, 1602888–1602888. 10.1002/aenm.201602888.

[ref7] Lu Z.; MacNeil D. D.; Dahn J. R. Layered Li[NixCo1–2xMnx]O2 Cathode Materials for Lithium-Ion Batteries. Electrochem. Solid-State Lett. 2001, 4 (12), A200 10.1149/1.1413182.

[ref8] Lu Z.; MacNeil D. D.; Dahn J. R. Layered Cathode Materials Li [ Ni x Li (1/3 - 2x/3) Mn (2/3 - x/3) ] O 2 for Lithium-Ion Batteries. Electrochem. Solid-State Lett. 2001, 4 (11), A191 10.1149/1.1407994.

[ref9] He P.; Yu H.; Li D.; Zhou H. Layered Lithium Transition Metal Oxide Cathodes towards High Energy Lithium-Ion Batteries. J. Mater. Chem. 2012, 22 (9), 3680–3695. 10.1039/c2jm14305d.

[ref10] Manthiram A.; Song B.; Li W. A Perspective on Nickel-Rich Layered Oxide Cathodes for Lithium-Ion Batteries. Energy Storage Mater. 2017, 6, 125–139. 10.1016/j.ensm.2016.10.007.

[ref11] Madhavi S.; Subba Rao G. V.; Chowdari B. V. R.; Li S. F. Y. Effect of Aluminium Doping on Cathodic Behaviour of LiNi0.7Co0.3O2. J. Power Sources 2001, 93 (1–2), 156–162. 10.1016/S0378-7753(00)00559-0.

[ref12] Weaving J. S.; Coowar F.; Teagle D. A.; Cullen J.; Dass V.; Bindin P.; Green R.; Macklin W. J. Development of High Energy Density Li-Ion Batteries Based on LiNi1-x-yCoxAlyO2. J. Power Sources 2001, 97–98, 733–735. 10.1016/S0378-7753(01)00700-5.

[ref13] Yabuuchi N.; Ohzuku T. Novel Lithium Insertion Material of LiCo1/3Ni1/3Mn1/3O2 for Advanced Lithium-Ion Batteries. J. Power Sources 2003, 119–121, 171–174. 10.1016/S0378-7753(03)00173-3.

[ref14] Delmas C.; Fouassier C.; Hagenmuller P. Structural Classification and Properties of the Layered Oxides. Phys. BC 1980, 99 (1–4), 81–85. 10.1016/0378-4363(80)90214-4.

[ref15] Xu C.; Märker K.; Lee J.; Mahadevegowda A.; Reeves P. J.; Day S. J.; Groh M. F.; Emge S. P.; Ducati C.; Layla Mehdi B.; Tang C. C.; Grey C. P. Bulk Fatigue Induced by Surface Reconstruction in Layered Ni-Rich Cathodes for Li-Ion Batteries. Nat. Mater. 2021, 20, 84–92. 10.1038/s41563-020-0767-8. 32839589

[ref16] Märker K.; Reeves P. J.; Xu C.; Griffith K. J.; Grey C. P. Evolution of Structure and Lithium Dynamics in LiNi 0.8 Mn 0.1 Co 0.1 O 2 (NMC811) Cathodes during Electrochemical Cycling. Chem. Mater. 2019, 31 (7), 2545–2554. 10.1021/acs.chemmater.9b00140.

[ref17] Grenier A.; Reeves P. J.; Liu H.; Seymour I. D.; Märker K.; Wiaderek K. M.; Chupas P. J.; Grey C. P.; Chapman K. W. Intrinsic Kinetic Limitations in Substituted Lithium Layered Transition-Metal Oxide Electrodes. J. Am. Chem. Soc. 2020, 142 (15), 7001–7011. 10.1021/jacs.9b13551. 32202112

[ref18] Lebens-Higgins Z. W.; Faenza N. V.; Radin M. D.; Liu H.; Sallis S.; Rana J.; Vinckeviciute J.; Reeves P. J.; Zuba M. J.; Badway F.; Pereira N.; Chapman K. W.; Lee T.-L.; Wu T.; Grey C. P.; Melot B. C.; Van Der Ven A.; Amatucci G. G.; Yang W.; Piper L. F. J. Revisiting the Charge Compensation Mechanisms in LiNi0.8Co0.2-yAlyO2 Systems. Mater. Horiz. 2019, 6 (10), 2112–2123. 10.1039/C9MH00765B.

[ref19] Lebens-Higgins Z. W.; Halat D. M.; Faenza N. V.; Wahila M. J.; Mascheck M.; Wiell T.; Eriksson S. K.; Palmgren P.; Rodriguez J.; Badway F.; Pereira N.; Amatucci G. G.; Lee T.-L.; Grey C. P.; Piper L. F. J. Surface Chemistry Dependence on Aluminum Doping in Ni-Rich LiNi0.8Co0.2-yAlyO2 Cathodes. Sci. Rep. 2019, 9 (1), 17720 10.1038/s41598-019-53932-6. 31776363 PMC6881288

[ref20] Croy J. R.; Balasubramanian M.; Kim D.; Kang S.-H.; Thackeray M. M. Designing High-Capacity, Lithium-Ion Cathodes Using X-Ray Absorption Spectroscopy. Chem. Mater. 2011, 23 (24), 5415–5424. 10.1021/cm2026703.

[ref21] Ceder G.; Chiang Y.-M.; Sadoway D. R.; Aydinol M. K.; Jang Y.-I.; Huang B. Identification of Cathode Materials for Lithium Batteries Guided by First-Principles Calculations. Nature 1998, 392 (6677), 694–696. 10.1038/33647.

[ref22] Meng Y. S.; Dompablo M. E. A. Recent Advances in First Principles Computational Research of Cathode Materials for Lithium-Ion Batteries. Acc. Chem. Res. 2013, 46 (5), 1171–1180. 10.1021/ar2002396. 22489876

[ref23] Seymour I. D.; Chakraborty S.; Middlemiss D. S.; Wales D. J.; Grey C. P. Mapping Structural Changes in Electrode Materials: Application of the Hybrid Eigenvector-Following Density Functional Theory (DFT) Method to Layered Li_0.5_MnO_2_. Chem. Mater. 2015, 27 (16), 5550–5561. 10.1021/acs.chemmater.5b01674.

[ref24] Seymour I. D.; Wales D. J.; Grey C. P. Preventing Structural Rearrangements on Battery Cycling: A First-Principles Investigation of the Effect of Dopants on the Migration Barriers in Layered Li 0.5 MnO 2. J. Phys. Chem. C 2016, 120 (35), 19521–19530. 10.1021/acs.jpcc.6b05307.

[ref25] Yoshida T.; Hongo K.; Maezono R. First-Principles Study of Structural Transitions in LiNiO2 and High-Throughput Screening for Long Life Battery. J. Phys. Chem. C 2019, 123 (23), 14126–14131. 10.1021/acs.jpcc.8b12556.

[ref26] Carlier D.; Ménétrier M.; Grey C. P.; Delmas C.; Ceder G. Understanding the NMR Shifts in Paramagnetic Transition Metal Oxides Using Density Functional Theory Calculations. Phys. Rev. B 2003, 67 (17), 174103 10.1103/PhysRevB.67.174103.

[ref27] Trease N. M.; Seymour I. D.; Radin M. D.; Liu H.; Liu H.; Hy S.; Chernova N.; Parikh P.; Devaraj A.; Wiaderek K. M.; Chupas P. J.; Chapman K. W.; Whittingham M. S.; Meng Y. S.; Van Der Van A.; Grey C. P. Identifying the Distribution of Al 3+ in LiNi_0.8_Co_0.15_Al_0.05_O_2_. Chem. Mater. 2016, 28 (22), 8170–8180. 10.1021/acs.chemmater.6b02797.

[ref28] Grey C. P.; Dupré N. NMR Studies of Cathode Materials for Lithium-Ion Rechargeable Batteries. Chem. Rev. 2004, 104 (10), 4493–4512. 10.1021/cr020734p. 15669160

[ref29] Halat D. M.; Dunstan M. T.; Gaultois M. W.; Britto S.; Grey C. P. Study of Defect Chemistry in the System La2-XSrxNiO4+δ by 17O Solid-State NMR Spectroscopy and Ni K-Edge XANES. Chem. Mater. 2018, 30 (14), 4556–4570. 10.1021/acs.chemmater.8b00747.

[ref30] Hope M. A.; Halat D. M.; Lee J.; Grey C. P. A 17O Paramagnetic NMR Study of Sm2O3, Eu2O3, and Sm/Eu-Substituted CeO2. Solid State Nucl. Magn. Reson. 2019, 102, 21–30. 10.1016/j.ssnmr.2019.05.010. 31226536

[ref31] Ashbrook S. E.; Smith M. E. Solid State 17O NMR—an Introduction to the Background Principles and Applications to Inorganic Materials. Chem. Soc. Rev. 2006, 35 (8), 718–735. 10.1039/B514051J. 16862272

[ref32] Kong X.; Terskikh V. V.; Khade R. L.; Yang L.; Rorick A.; Zhang Y.; He P.; Huang Y.; Wu G. Solid-State 17O NMR Spectroscopy of Paramagnetic Coordination Compounds. Angew. Chem., Int. Ed. 2015, 54 (16), 4753–4757. 10.1002/anie.201409888. PMC4418630 25694203

[ref33] Verkhovskii S.; Trokiner A.; Gerashenko A.; Yakubovskii A.; Medvedeva N.; Litvinova Z.; Mikhalev K.; Buzlukov A. 17O NMR Evidence for Vanishing of Magnetic Polarons in the Paramagnetic Phase of Ceramic CaMnO3. Phys. Rev. B 2010, 81 (14), 144415 10.1103/PhysRevB.81.144415.

[ref34] Ashbrook S. E.; Berry A. J.; Frost D. J.; Gregorovic A.; Pickard C. J.; Readman J. E.; Wimperis S. 17O and 29Si NMR Parameters of MgSiO3 Phases from High-Resolution Solid-State NMR Spectroscopy and First-Principles Calculations. J. Am. Chem. Soc. 2007, 129 (43), 13213–13224. 10.1021/ja074428a. 17924628

[ref35] Hope M. A.; Halat D. M.; Magusin P. C. M. M.; Paul S.; Peng L.; Grey C. P. Surface-Selective Direct 17 O DNP NMR of CeO 2 Nanoparticles. Chem. Commun. 2017, 53 (13), 2142–2145. 10.1039/C6CC10145C. 28134945

[ref36] Peng L.; Liu Y.; Kim N.; Readman J. E.; Grey C. P. Detection of Brønsted Acid Sites in Zeolite HY with High-Field 17O-MAS-NMR Techniques. Nat. Mater. 2005, 4 (3), 216–219. 10.1038/nmat1332. 15711551

[ref37] Geng F.; Hu B.; Li C.; Zhao C.; Lafon O.; Trébosc J.; Amoureux J. P.; Shen M.; Hu B. Anionic Redox Reactions and Structural Degradation in a Cation-Disordered Rock-Salt Li1.2Ti0.4Mn0.4O2cathode Material Revealed by Solid-State NMR and EPR. J. Mater. Chem. A 2020, 8 (32), 16515–16526. 10.1039/D0TA03358H.

[ref38] Geng F.; Shen M.; Hu B.; Liu Y.; Zeng L.; Hu B. Monitoring the Evolution of Local Oxygen Environments during LiCoO_2_ Charging: Via Ex Situ 17O NMR. Chem. Commun. 2019, 55 (52), 7550–7553. 10.1039/C9CC03304A. 31188369

[ref39] House R. A.; Rees G. J.; Pérez-Osorio M. A.; Marie J.-J.; Boivin E.; Robertson A. W.; Nag A.; Garcia-Fernandez M.; Zhou K.-J.; Bruce P. G. First-Cycle Voltage Hysteresis in Li-Rich 3d Cathodes Associated with Molecular O2 Trapped in the Bulk. Nat. Energy 2020, 5, 777–785. 10.1038/s41560-020-00697-2.

[ref40] Seymour I. D.; Middlemiss D. S.; Halat D. M.; Trease N. M.; Pell A. J.; Grey C. P. Characterizing Oxygen Local Environments in Paramagnetic Battery Materials via 17O NMR and DFT Calculations. J. Am. Chem. Soc. 2016, 138 (30), 9405–9408. 10.1021/jacs.6b05747. 27404908

[ref41] Reeves P. J.; Seymour I. D.; Griffith K. J.; Grey C. P. Characterizing the Structure and Phase Transition of Li 2 RuO 3 Using Variable-Temperature 17 O and 7 Li NMR Spectroscopy. Chem. Mater. 2019, 31 (8), 2814–2821. 10.1021/acs.chemmater.8b05178.

[ref42] Griffin J. M.; Clark L.; Seymour V. R.; Aldous D. W.; Dawson D. M.; Iuga D.; Morris R. E.; Ashbrook S. E. Ionothermal 17 O Enrichment of Oxides Using Microlitre Quantities of Labelled Water. Chem. Sci. 2012, 3 (7), 2293–2300. 10.1039/c2sc20155k.

[ref43] Gerothanassis I. P. Oxygen-17 NMR Spectroscopy: Basic Principles and Applications (Part I). Prog. Nucl. Magn. Reson. Spectrosc. 2010, 56 (2), 95–197. 10.1016/j.pnmrs.2009.09.002. 20633350

[ref44] Gerothanassis I. P. Oxygen-17 NMR Spectroscopy: Basic Principles and Applications (Part II). Prog. Nucl. Magn. Reson. Spectrosc. 2010, 57 (1), 1–110. 10.1016/j.pnmrs.2009.12.001. 20633360

[ref45] Pell A. J.; Pintacuda G.; Grey C. P. Paramagnetic NMR in Solution and the Solid State. Prog. Nucl. Magn. Reson. Spectrosc. 2019, 111 (May), 1–271. 10.1016/j.pnmrs.2018.05.001. 31146806

[ref46] Kim J.; Middlemiss D. S.; Chernova N. A.; Zhu B. Y. X.; Masquelier C.; Grey C. P. Linking Local Environments and Hyperfine Shifts: A Combined Experimental and Theoretical 31 P and 7 Li Solid-State NMR Study of Paramagnetic Fe(III) Phosphates. J. Am. Chem. Soc. 2010, 132 (47), 16825–16840. 10.1021/ja102678r. 21053901

[ref47] Clément R. J.; Pell A. J.; Middlemiss D. S.; Strobridge F. C.; Miller J. K.; Whittingham M. S.; Emsley L.; Grey C. P.; Pintacuda G. Spin-Transfer Pathways in Paramagnetic Lithium Transition-Metal Phosphates from Combined Broadband Isotropic Solid-State MAS NMR Spectroscopy and DFT Calculations. J. Am. Chem. Soc. 2012, 134 (41), 17178–17185. 10.1021/ja306876u. 23004936

[ref48] Goodenough J. B. An Interpretation of the Magnetic Properties of the Perovskite-Type Mixed Crystals La(1-x)Sr(x)CoO(3-y). J. Phys. Chw Solids Pergamon Press 1958, 6, 287–297. 10.1016/0022-3697(58)90107-0.

[ref49] Kanamori J. Theory of the Magnetic Properties of Ferrous and Cobaltous Oxides, I. Prog. Theor. Phys. 1957, 17 (2), 177–196. 10.1143/PTP.17.177.

[ref50] Kanamori J. Theory of the Magnetic Properties of Ferrous and Cobaltous Oxides, II. Prog. Theor. Phys. 1957, 17 (2), 197–222. 10.1143/PTP.17.197.

[ref51] Pell A. J.; Clément R. J.; Grey C. P.; Emsley L.; Pintacuda G. Frequency-Stepped Acquisition in Nuclear Magnetic Resonance Spectroscopy under Magic Angle Spinning. J. Chem. Phys. 2013, 138 (11), 114201 10.1063/1.4795001. 23534632

[ref52] Schramm S.; Oldfield E. High-Resolution Oxygen-17 NMR of Solids. J. Am. Chem. Soc. 1984, 106 (9), 2502–2506. 10.1021/ja00321a002.

[ref53] Bréger J.; Jiang M.; Dupré N.; Meng Y. S.; Shao-Horn Y.; Ceder G.; Grey C. P. High-Resolution X-Ray Diffraction, DIFFaX, NMR and First Principles Study of Disorder in the Li 2MnO 3-Li[Ni 1/2Mn 1/2]O 2 Solid Solution. J. Solid State Chem. 2005, 178 (9), 2575–2585. 10.1016/j.jssc.2005.05.027.

[ref54] Miura Y.; Sato M.; Yamakawa Y.; Habaguchi T.; O̅no Y. Structural Transition of Li 2 RuO 3 Induced by Molecular-Orbit Formation. J. Phys. Soc. Jpn. 2009, 78, 094706 10.1143/JPSJ.78.094706.

[ref55] Levitt M. H. Motional Lineshapes and Two-Site Exchange. Spin Dynamics; John Wiley & Sons Ltd: Southampton, 2007; pp 516–527.

[ref56] Ménétrier M.; Saadoune I.; Levasseur S.; Delmas C. The Insulator-Metal Transition upon Lithium Deintercalation from LiCoO_2_: Electronic Properties and 7Li NMR Study. J. Mater. Chem. 1999, 9 (5), 1135–1140. 10.1039/a900016j.

[ref57] Zhuo Z.; Liu Y.; Guo J.; Chuang Y.; Pan F.; Yang W. Full Energy Range Resonant Inelastic X-Ray Scattering of O 2 and CO 2 : Direct Comparison with Oxygen Redox State in Batteries. J. Phys. Chem. Lett. 2020, 11 (7), 2618–2623. 10.1021/acs.jpclett.0c00423. 32154725

[ref58] Li N.; Sallis S.; Papp J. K.; Wei J.; McCloskey B. D.; Yang W.; Tong W. Unraveling the Cationic and Anionic Redox Reactions in a Conventional Layered Oxide Cathode. ACS Energy Lett. 2019, 4 (12), 2836–2842. 10.1021/acsenergylett.9b02147.

[ref59] Wu J.; Li Q.; Sallis S.; Zhuo Z.; Gent W. E.; Chueh W. C.; Yan S.; Chuang Y.-d.; Yang W. Fingerprint Oxygen Redox Reactions in Batteries through High-Efficiency Mapping of Resonant Inelastic X-Ray Scattering. Condens. Matter 2019, 4 (1), 5 10.3390/condmat4010005.

[ref60] Rong X.; Liu J.; Hu E.; Liu Y.; Wang Y.; Wu J.; Yu X.; Page K.; Hu Y.-S.; Yang W.; Li H.; Yang X.-Q.; Chen L.; Huang X. Structure-Induced Reversible Anionic Redox Activity in Na Layered Oxide Cathode. Joule 2018, 2 (1), 125–140. 10.1016/j.joule.2017.10.008.

[ref61] Whittingham M. S. Lithium Batteries and Cathode Materials. Chem. Rev. 2004, 104 (10), 4271–4301. 10.1021/cr020731c. 15669156

[ref62] Amatucci G. G.; Tarascon J. M.; Klein L. C. CoO2, The End Member of the Li x CoO2 Solid Solution. J. Electrochem. Soc. 1996, 143 (3), 1114 10.1149/1.1836594.

[ref63] Van der Ven A.; Aydinol M. K.; Ceder G.; Kresse G.; Hafner J. First-Principles Investigation of Phase Stability in LixCoO2. Phys. Rev. B 1998, 58 (6), 2975 10.1103/PhysRevB.58.2975.

[ref64] Chen Z.; Lu Z.; Dahn J. R. Staging Phase Transitions in Li x CoO 2. J. Electrochem. Soc. 2002, 149 (12), A1604 10.1149/1.1519850.

[ref65] Rinkel B. L. D.; Hall D. S.; Temprano I.; Grey C. P. Electrolyte Oxidation Pathways in Lithium-Ion Batteries. J. Am. Chem. Soc. 2020, 142 (35), 15058–15074. 10.1021/jacs.0c06363. 32697590

[ref66] Yano A.; Shikano M.; Ueda A.; Sakaebe H.; Ogumi Z. LiCoO_2_ Degradation Behavior in the High-Voltage Phase Transition Region and Improved Reversibility with Surface Coating. J. Electrochem. Soc. 2017, 164 (1), A6116 10.1149/2.0181701jes.

[ref67] Yang X.; Lin M.; Zheng G.; Wu J.; Wang X.; Ren F.; Zhang W.; Liao Y.; Zhao W.; Zhang Z.; Xu N.; Yang W.; Yang Y. Enabling Stable High-Voltage LiCoO_2_ Operation by Using Synergetic Interfacial Modification Strategy. Adv. Funct. Mater. 2020, 30 (43), 2004664 10.1002/adfm.202004664.

[ref68] Xu N.; Shi J.; Liu G.; Yang X.; Zheng J.; Zhang Z.; Yang Y. Research Progress of Fluorine-Containing Electrolyte Additives for Lithium Ion Batteries. J. Power Sources Adv. 2021, 7, 100043 10.1016/j.powera.2020.100043.

[ref69] Takahashi Y.; Kijima N.; Tokiwa K.; Watanabe T.; Akimoto J. Single-Crystal Synthesis, Structure Refinement and Electrical Properties of Li0.5CoO2. J. Phys.: Condens. Matter 2007, 19, 436202 10.1088/0953-8984/19/43/436202.

[ref70] Assat G.; Delacourt C.; Corte D. A. D.; Tarascon J.-M. Editors’ Choice—Practical Assessment of Anionic Redox in Li-Rich Layered Oxide Cathodes: A Mixed Blessing for High Energy Li-Ion Batteries. J. Electrochem. Soc. 2016, 163 (14), A2965–A2976. 10.1149/2.0531614jes.

[ref71] Fong R.; Dahn J. R.; Jones C. H. W. Electrochemistry of Pyrite-Based Cathodes for Ambient Temperature Lithium Batteries. J. Electrochem. Soc. 1989, 136 (11), 3206–3206. 10.1149/1.2096426.

[ref72] Goodenough J. B.; Kim Y. Locating Redox Couples in the Layered Sulfides with Application to Cu[Cr2]S4. J. Solid State Chem. 2009, 182 (10), 2904–2911. 10.1016/j.jssc.2009.08.005.

[ref73] Saha S.; Assat G.; Sougrati M. T.; Foix D.; Li H.; Vergnet J.; Turi S.; Ha Y.; Yang W.; Cabana J.; Rousse G.; Abakumov A. M.; Tarascon J. M. Exploring the Bottlenecks of Anionic Redox in Li-Rich Layered Sulfides. Nat. Energy 2019, 4 (11), 977–987. 10.1038/s41560-019-0493-0.

[ref74] Hansen C. J.; Zak J. J.; Martinolich A. J.; Ko J. S.; Bashian N. H.; Kaboudvand F.; Van Der Ven A.; Melot B. C.; Nelson Weker J.; See K. A. Multielectron, Cation and Anion Redox in Lithium-Rich Iron Sulfide Cathodes. J. Am. Chem. Soc. 2020, 142 (14), 6737–6749. 10.1021/jacs.0c00909. 32223192

[ref75] Bonaventura C.; Henkens R.; Alayash A. I.; Banerjee S.; Crumbliss A. L. Molecular Controls of the Oxygenation and Redox Reactions of Hemoglobin. Antioxid. Redox. Signal. 2013, 18 (17), 2298–2313. 10.1089/ars.2012.4947. 23198874 PMC4047995

[ref76] House R. A.; Marie J.-J.; Pérez-Osorio M. A.; Rees G. J.; Boivin E.; Bruce P. G. The Role of O2 in O-Redox Cathodes for Li-Ion Batteries. Nat. Energy 2021, 6, 781–789. 10.1038/s41560-021-00780-2.

[ref77] House R. A.; Maitra U.; Jin L.; Lozano J. G.; Somerville J. W.; Rees N. H.; Naylor A. J.; Duda L. C.; Massel F.; Chadwick A. V.; Ramos S.; Pickup D. M.; McNally D. E.; Lu X.; Schmitt T.; Roberts M. R.; Bruce P. G. What Triggers Oxygen Loss in Oxygen Redox Cathode Materials?. Chem. Mater. 2019, 31 (9), 3293–3300. 10.1021/acs.chemmater.9b00227.

[ref78] McCalla E.; Sougrati M. T.; Rousse G.; Berg E. J.; Abakumov A.; Recham N.; Ramesha K.; Sathiya M.; Dominko R.; Van Tendeloo G.; Novák P.; Tarascon J.-M. Understanding the Roles of Anionic Redox and Oxygen Release during Electrochemical Cycling of Lithium-Rich Layered Li 4 FeSbO 6. J. Am. Chem. Soc. 2015, 137 (14), 4804–4814. 10.1021/jacs.5b01424. 25811894

[ref79] McCalla E.; Abakumov A. M.; Saubanère M.; Foix D.; Berg E. J.; Rousse G.; Doublet M.-L.; Gonbeau D.; Novák P.; Van Tendeloo G.; Dominko R.; Tarascon J.-M. Visualization of O-O Peroxo-like Dimers in High-Capacity Layered Oxides for Li-Ion Batteries. Science 2015, 350 (6267), 1516–1521. 10.1126/science.aac8260. 26680196

[ref80] Grimaud A.; Hong W. T.; Shao-Horn Y.; Tarascon J.-M. Anionic Redox Processes for Electrochemical Devices. Nat. Mater. 2016, 15 (2), 121–126. 10.1038/nmat4551. 26796721

[ref81] Kitchaev D. A.; Vinckeviciute J.; Van Der Ven A. Delocalized Metal-Oxygen π-Redox Is the Origin of Anomalous Nonhysteretic Capacity in Li-Ion and Na-Ion Cathode Materials. J. Am. Chem. Soc. 2021, 143 (4), 1908–1916. 10.1021/jacs.0c10704. 33481574

[ref82] Radin M. D.; Vinckeviciute J.; Seshadri R.; Van der Ven A. Manganese Oxidation as the Origin of the Anomalous Capacity of Mn-Containing Li-Excess Cathode Materials. Nat. Energy 2019, 4, 639–646. 10.1038/s41560-019-0439-6.

[ref83] Vinckeviciute J.; Kitchaev D. A.; Van der Ven A. A Two-Step Oxidation Mechanism Controlled by Mn Migration Explains the First-Cycle Activation Behavior of Li2MnO3-Based Li-Excess Materials. Chem. Mater. 2021, 33 (5), 1625–1636. 10.1021/acs.chemmater.0c03734.

[ref84] Seo D.-H.; Lee J.; Urban A.; Malik R.; Kang S.; Ceder G. The Structural and Chemical Origin of the Oxygen Redox Activity in Layered and Cation-Disordered Li-Excess Cathode Materials. Nat. Chem. 2016, 8 (7), 692–697. 10.1038/nchem.2524. 27325096

[ref85] House R. A.; Maitra U.; Pérez-osorio M. A.; Lozano J. G.; Jin L.; Somerville J. W.; Duda L. C.; Nag A.; Walters A.; Zhou K.; Roberts M. R.; Bruce P. G. Superstructure Control of First-Cycle Voltage Hysteresis in O-Redox Cathodes. Nature 2020, 577, 502–508. 10.1038/s41586-019-1854-3. 31816625

[ref86] Luo K.; Roberts M. R.; Hao R.; Guerrini N.; Pickup D. M.; Liu Y.-S.; Edström K.; Guo J.; Chadwick A. V.; Duda L. C.; Bruce P. G. Charge-Compensation in 3d-Transition-Metal-Oxide Intercalation Cathodes through the Generation of Localized Electron Holes on Oxygen. Nat. Chem. 2016, 8. 684 10.1038/nchem.2471. 27325095

[ref87] Luo K.; Roberts M. R.; Guerrini N.; Tapia-Ruiz N.; Hao R.; Massel F.; Pickup D. M.; Ramos S.; Liu Y.-S.; Guo J.; Chadwick A. V.; Duda L. C.; Bruce P. G. Anion Redox Chemistry in the Cobalt Free 3d Transition Metal Oxide Intercalation Electrode Li[Li 0.2 Ni 0.2 Mn 0.6]O 2. J. Am. Chem. Soc. 2016, 138 (35), 11211–11218. 10.1021/jacs.6b05111. 27498756

[ref88] Hong J.; Gent W. E.; Xiao P.; Lim K.; Seo D.-H.; Wu J.; Csernica P. M.; Takacs C. J.; Nordlund D.; Sun C.-J.; Stone K. H.; Passarello D.; Yang W.; Prendergast D.; Ceder G.; Toney M. F.; Chueh W. C. Metal-Oxygen Decoordination Stabilizes Anion Redox in Li-Rich Oxides. Nat. Mater. 2019, 18 (3), 256–265. 10.1038/s41563-018-0276-1. 30718861

[ref89] Gent W. E.; Lim K.; Liang Y.; Li Q.; Barnes T.; Ahn S.-J.; Stone K. H.; McIntire M.; Hong J.; Song J. H.; Li Y.; Mehta A.; Ermon S.; Tyliszczak T.; Kilcoyne D.; Vine D.; Park J.-H.; Doo S.-K.; Toney M. F.; Yang W.; Prendergast D.; Chueh W. C. Coupling between Oxygen Redox and Cation Migration Explains Unusual Electrochemistry in Lithium-Rich Layered Oxides. Nat. Commun. 2017, 8 (1), 2091–2091. 10.1038/s41467-017-02041-x. 29233965 PMC5727078

[ref90] Gent W. E.; Abate I. I.; Yang W.; Nazar L. F.; Chueh W. C. Design Rules for High-Valent Redox in Intercalation Electrodes. Joule 2020, 4 (7), 1369–1397. 10.1016/j.joule.2020.05.004.

[ref91] Zhang M.; Kitchaev D. A.; Lebens-Higgins Z.; Vinckeviciute J.; Zuba M.; Reeves P. J.; Grey C. P.; Whittingham M. S.; Piper L. F. J.; Van der Ven A.; Meng Y. S. Pushing the Limit of 3d Transition Metal-Based Layered Oxides That Use Both Cation and Anion Redox for Energy Storage. Nat. Rev. Mater. 2022, 7, 522–540. 10.1038/s41578-022-00416-1.

[ref92] Lebens-Higgins Z. W.; Chung H.; Zuba M. J.; Rana J.; Li Y.; Faenza N. V.; Pereira N.; McCloskey B. D.; Rodolakis F.; Yang W.; Whittingham M. S.; Amatucci G. G.; Meng Y. S.; Lee T. L.; Piper L. F. J. How Bulk Sensitive Is Hard X-Ray Photoelectron Spectroscopy: Accounting for the Cathode-Electrolyte Interface When Addressing Oxygen Redox. J. Phys. Chem. Lett. 2020, 11 (6), 2106–2112. 10.1021/acs.jpclett.0c00229. 32101006

[ref93] Lebens-Higgins Z. W.; Vinckeviciute J.; Wu J.; Faenza N. V.; Li Y.; Sallis S.; Pereira N.; Meng Y. S.; Amatucci G. G.; Der Ven A. V.; Yang W.; Piper L. F. J. Distinction between Intrinsic and X-Ray-Induced Oxidized Oxygen States in Li-Rich 3d Layered Oxides and LiAlO2. J. Phys. Chem. C 2019, 123 (21), 13201–13207. 10.1021/acs.jpcc.9b01298.

[ref94] Yang W.; Devereaux T. P. Anionic and Cationic Redox and Interfaces in Batteries: Advances from Soft X-Ray Absorption Spectroscopy to Resonant Inelastic Scattering. J. Power Sources 2018, 389, 188–197. 10.1016/j.jpowsour.2018.04.018.

[ref95] Maitra U.; House R. A.; Somerville J. W.; Tapia-Ruiz N.; Lozano J. G.; Guerrini N.; Hao R.; Luo K.; Jin L.; Pérez-Osorio M. A.; Massel F.; Pickup D. M.; Ramos S.; Lu X.; McNally D. E.; Chadwick A. V.; Giustino F.; Schmitt T.; Duda L. C.; Roberts M. R.; Bruce P. G. Oxygen Redox Chemistry without Excess Alkali-Metal Ions in Na2/3[Mg0.28Mn0.72]O2. Nat. Chem. 2018, 10 (3), 288–295. 10.1038/nchem.2923. 29461536

[ref96] Csernica P. M.; Kalirai S. S.; Gent W. E.; Lim K.; Yu Y.-S.; Liu Y.; Ahn S.-J.; Kaeli E.; Xu X.; Stone K. H.; Marshall A. F.; Sinclair R.; Shapiro D. A.; Toney M. F.; Chueh W. C. Persistent and Partially Mobile Oxygen Vacancies in Li-Rich Layered Oxides. Nat. Energy 2021, 6, 642–652. 10.1038/s41560-021-00832-7.

[ref97] Saubanère M.; McCalla E.; Tarascon J.-M.; Doublet M.-L. The Intriguing Question of Anionic Redox in High-Energy Density Cathodes for Li-Ion Batteries. Energy Environ. Sci. 2016, 9 (3), 984–991. 10.1039/C5EE03048J.

[ref98] Song B.; Tang M.; Hu E.; Borkiewicz O. J.; Wiaderek K. M.; Zhang Y.; Phillip N. D.; Liu X.; Shadike Z.; Li C.; Song L.; Hu Y.-Y.; Chi M.; Veith G. M.; Yang X.-Q.; Liu J.; Nanda J.; Page K.; Huq A. Understanding the Low-Voltage Hysteresis of Anionic Redox in Na 2 Mn 3 O 7. Chem. Mater. 2019, 31 (10), 3756–3765. 10.1021/acs.chemmater.9b00772.

[ref99] Jones M. A.; Reeves P. J.; Seymour I. D.; Cliffe M. J.; Dutton S. E.; Grey C. P. Short-Range Ordering in a Battery Electrode, the ‘Cation-Disordered’ Rocksalt Li1.25Nb0.25Mn0.5O2. Chem. Commun. 2019, 55, 9027–9030. 10.1039/C9CC04250D. 31290883

[ref100] Ohzuku T.; Makimura Y. Layered Lithium Insertion Material of LiNi1/2Mn1/2O2 : A Possible Alternative to LiCoO_2_ for Advanced Lithium-Ion Batteries. Chem. Lett. 2001, 30 (8), 744–745. 10.1246/cl.2001.744.

[ref101] Yabuuchi N.; Nakayama M.; Takeuchi M.; Komaba S.; Hashimoto Y.; Mukai T.; Shiiba H.; Sato K.; Kobayashi Y.; Nakao A.; Yonemura M.; Yamanaka K.; Mitsuhara K.; Ohta T. Origin of Stabilization and Destabilization in Solid-State Redox Reaction of Oxide Ions for Lithium-Ion Batteries. Nat. Commun. 2016, 7 (1), 13814–13814. 10.1038/ncomms13814. 28008955 PMC5196437

[ref102] Wang R.; Li X.; Liu L.; Lee J.; Seo D.-H.; Bo S.-H.; Urban A.; Ceder G. A Disordered Rock-Salt Li-Excess Cathode Material with High Capacity and Substantial Oxygen Redox Activity: Li 1.25 Nb 0.25 Mn 0.5 O 2. Electrochem. Commun. 2015, 60, 70–73. 10.1016/j.elecom.2015.08.003.

[ref103] Kan W. H.; Chen D.; Papp J. K.; Shukla A. K.; Huq A.; Brown C. M.; McCloskey B. D.; Chen G. Unravelling Solid-State Redox Chemistry in Li1.3Nb0.3Mn0.4O2 Single-Crystal Cathode Material. Chem. Mater. 2018, 30 (5), 1655–1666. 10.1021/acs.chemmater.7b05036.

[ref104] Li Y.; Wu X. P.; Jiang N.; Lin M.; Shen L.; Sun H.; Wang Y.; Wang M.; Ke X.; Yu Z.; Gao F.; Dong L.; Guo X.; Hou W.; Ding W.; Gong X. Q.; Grey C. P.; Peng L. Distinguishing Faceted Oxide Nanocrystals with 17O Solid-State NMR Spectroscopy. Nat. Commun. 2017, 8 (1), 581 10.1038/s41467-017-00603-7. 28924155 PMC5603560

[ref105] Leskes M.; Moore A. J.; Goward G. R.; Grey C. P. Monitoring the Electrochemical Processes in the Lithium-Air Battery by Solid State NMR Spectroscopy. J. Phys. Chem. C 2013, 117 (51), 26929–26939. 10.1021/jp410429k. PMC3905693 24489976

[ref106] Dundon J. M. 17O NMR in Liquid O2. J. Chem. Phys. 1982, 76 (5), 2171–2173. 10.1063/1.443233.

[ref107] Oldfield E.; Lee H. C.; Coretsopoulos C.; Adebodun F.; Park K. D.; Yang S.; Chung J.; Phillips B. Solid-State Oxygen-17 Nuclear Magnetic Resonance Spectroscopic Studies of [17O2] Picket Fence Porphyrin, Myoglobin, and Hemoglobin. J. Am. Chem. Soc. 1991, 113 (23), 8680–8685. 10.1021/ja00023a015.

[ref108] Li X.; Li X.; Monluc L.; Chen B.; Tang M.; Chien P.-H.; Feng X.; Hung I.; Gan Z.; Urban A.; Hu Y.-Y. Stacking-Fault Enhanced Oxygen Redox in Li2MnO3. Adv. Energy Mater. 2022, 12 (18), 2200427 10.1002/aenm.202200427.

[ref109] Larsen F. H.; Jakobsen H. J.; Ellis P. D.; Nielsen N. C. QCPMG-MAS NMR of Half-Integer Quadrupolar Nuclei. J. Magn. Reson. 1998, 131 (1), 144–147. 10.1006/jmre.1997.1341. 9533917

[ref110] O’Dell L. A.; Schurko R. W. QCPMG Using Adiabatic Pulses for Faster Acquisition of Ultra-Wideline NMR Spectra. Chem. Phys. Lett. 2008, 464 (1–3), 97–102. 10.1016/j.cplett.2008.08.095.

[ref111] Rana J.; Papp J. K.; Lebens-Higgins Z.; Zuba M.; Kaufman L. A.; Goel A.; Schmuch R.; Winter M.; Whittingham M. S.; Yang W.; McCloskey B. D.; Piper L. F. J. Quantifying the Capacity Contributions during Activation of Li2MnO3. ACS Energy Lett. 2020, 5 (2), 634–641. 10.1021/acsenergylett.9b02799.

[ref112] Yan P.; Xiao L.; Zheng J.; Zhou Y.; He Y.; Zu X.; Mao S. X.; Xiao J.; Gao F.; Zhang J. G.; Wang C. M. Probing the Degradation Mechanism of Li2MnO3 Cathode for Li-Ion Batteries. Chem. Mater. 2015, 27 (3), 975–982. 10.1021/cm504257m.

[ref113] Dogan F.; Croy J. R.; Balasubramanian M.; Slater M. D.; Iddir H.; Johnson C. S.; Vaughey J. T.; Key B. Solid State NMR Studies of Li 2 MnO 3 and Li-Rich Cathode Materials: Proton Insertion, Local Structure, and Voltage Fade. J. Electrochem. Soc. 2015, 162 (1), A235–A243. 10.1149/2.1041501jes.

[ref114] Wolf T.; Kumar S.; Singh H.; Chakrabarty T.; Aussenac F.; Frenkel A. I.; Major D. T.; Leskes M. Endogenous Dynamic Nuclear Polarization for Natural Abundance 17 O and Lithium NMR in the Bulk of Inorganic Solids. J. Am. Chem. Soc. 2019, 141 (1), 451–462. 10.1021/jacs.8b11015. 30525555

