# Supplementary material for: 17O NMR Spectroscopy in Lithium-Ion Battery Cathode Materials: Challenges and Interpretation
Source: J Am Chem Soc. 2022 Oct 6;144(41):18714–29. doi: 10.1021/jacs.2c02927 (PMC9585580; doi:10.1021/jacs.2c02927)
Supplement: Supplementary file 1 — ja2c02927_si_001.pdf [file ja2c02927_si_001.pdf]

Supporting Information

^{17}O NMR Spectroscopy in Lithium-ion Battery Cathode Materials: Challenges and Interpretation

Euan N. Bassey,[1] Philip J. Reeves,[1] Ieuan D. Seymour,[1,2] Clare P. Grey[1,*]

[1]Department of Chemistry, University of Cambridge, Lensfield Road, Cambridge, CB2 1EW, United Kingdom

[2]Department of Materials, Imperial College London, South Kensington Campus, London, SW7 2AZ, United Kingdom

* To whom correspondence should be addressed:

E-mail: cpg27@cam.ac.uk

Contents

1. **Variable Offset Cumulative Spectroscopy (VOCS)**
2. **The Quadrupolar Interaction in ^{17}O NMR**
3. **Calculation of ^{17}O NMR Parameters**
4. **Origin of Broadening of ^{17}O NMR Spectra for NCA**
5. **Anomalous "Negative Intensity" in ^{17}O NMR Spectra of Li_xCoO_2**
6. **Oxygen Redox Mechanisms**
7. **Pulse Sequences used in ^{17}O NMR**
8. **Comment on Li_2MnO_3 Bond Pathways**
9. **Experimental and Additional NMR data for NCA**
10. **Comment on EPR of $Li_{1.2}Ti_{0.4}Mn_{0.4}O_2$**
11. **Curie-Weiss Shift Conversion Calculations**
12. **Results obtained from *ex situ* and *in situ* ^{17}O NMR of Li_2MnO_3**

List of Figures

Figure S1: Schematic of VOCS
Figure S2: Schematic describing the effect of the quadrupolar interaction on nuclear spin microstate energies
Figure S3: Schematic nutation profile for a quadrupolar nucleus under selective and non-selective radiofrequency pulses.
Figure S4: Peroxo-formation mechanism for O redox and TEM evidence.
Figure S5: O_2-trapping stabilisation mechanism for O redox.
Figure S6: Evidence of localised holes in $Li_xNi_{0.13}Co_{0.13}Mn_{0.54}O_2$.
Figure S7: Schematics showing the *TM* migration mechanism for oxidised O stabilisation.
Figure S8: Schematics showing the mechanism and structural consequences of π-redox for stabilising oxidised O species.
Figure S9: Schematic showing the pre-saturation Hahn-echo pulse sequence
Figure S10: Pristine NCA characterisation by ^{27}Al and ^{59}Co NMR.
Figure S11: Fitted ^{27}Al NMR spectra of pristine NCA, acquired with selective and non-selective radiofrequency pulses.
Figure S12: Fitted ^{59}Co NMR spectra for pristine NCA, acquired with selective and non-selective radiofrequency pulses.

Figure S13: Schematics showing different bond pathways between Ni^{3+} and Al or Co in the NCA lattice.
Figure S14: *Ex situ* ^{27}Al NMR spectra of NCA at different states of charge.
Figure S15: *Ex situ* ^{59}Co NMR spectra of NCA at different states of charge.
Figure S16: Analysis of ^{59}Co NMR shifts, integrals and bond lengths.
Figure S17: *Ex situ* and *in situ* ^{17}O NMR spectra for Li_2MnO_3

List of Tables

Table S1: Comparison of DFT-calculated bond pathways for ^{27}Al and ^{59}Co
Table S2: Fitted parameters for ^{27}Al and ^{59}Co NMR spectra of pristine NCA
Table S3: Probability distribution for different coordination environments in NCA and comparison to fraction seen in NMR spectra.

1. Variable Offset Cumulative Spectroscopy (VOCS)

Since the ^{17}O NMR spectra of LiTMO_2 materials typically span several thousand ppm, full excitation of the spectrum is not possible using a single pulse at one receiver offset frequency. Instead, variable offset cumulative spectroscopy (VOCS) is required. Here, the receiver in the NMR circuit is stepped over a range of frequencies (the step size is generally chosen as ω_1, the frequency of the exciting pulse, to ensure at least 90% of the full intensity is recorded[1]), such that the overall spectrum is generated by appropriately summing over the slices [Figure S1].[2]

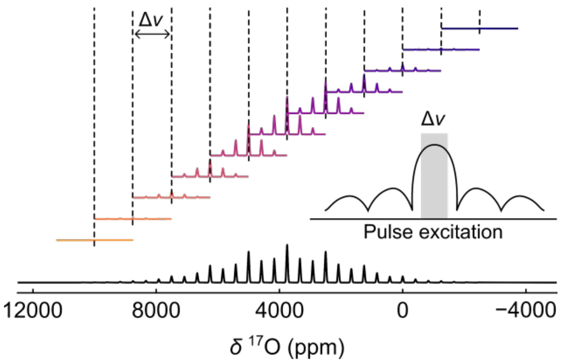

Figure S1: Schematic highlighting the variable offset cumulative spectrum (VOCS) method. Individual slices which span the width of the excitation pulse (top spectra, in colour) are summed to give the overall spectrum (bottom spectrum, in black). Slices are stepped by an offset, Δv, chosen to cover at least 90% of the excitation intensity (ca. half the pulse length).

2. The Quadrupolar Interaction in ^{17}O NMR

The ^{17}O quadrupole moment interacts with the local electric field gradient (EFG) at the O site [Figure S2(a)], shifting the energies of the different nuclear spin microstates and altering the appearance of the NMR spectrum [Figure S2(b)].[3–5] This interaction is spatially anisotropic and its strength is measured through the quadrupolar coupling constant, C_Q. Whilst MAS can mitigate the effect of spatially anisotropic interactions, in the case of strong quadrupolar coupling (at least 10% of the nuclear Zeeman splitting[4])—as commonly seen in the ^{17}O NMR spectra of LIB cathodes—MAS is unable to completely average out these interactions, resulting in a broad spinning sideband manifold, broadening of the individual sidebands and central transition (or isotropic resonance), and a field-dependent contribution to the chemical shift (known as the quadrupole-induced shift, QIS).[6]

As discussed in the main text, the effect of the quadrupolar interaction may be mitigated by increasing the field strength at which the experiments are performed (as the quadrupolar splitting scales with the nuclear Larmor frequency), thereby reducing the size of the QIS and the broadening of the central transition, CT, between the m_I = +1/2 and −1/2 microstates (where m_I is the magnetic nuclear spin quantum number) and sidebands.[3,4,7] We note that, in addition to the central transition, CT, satellite transitions (STs) corresponding to m_I = 3/2 to ½ and from m_I = 5/2 to 3/2, may be observed which under MAS result in broad sideband manifold;

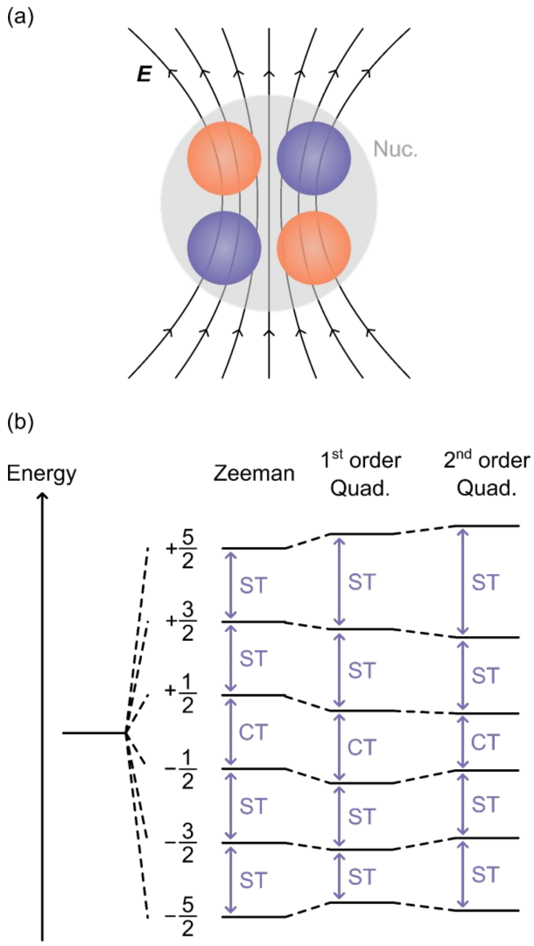

Figure S2: NMR spectroscopy of quadrupolar nuclei; **(a)** shows a cartoon representation of the interaction between the nuclear quadrupole moment and local electric field gradient, **E. (b)** shows the energies of the nuclear spin microstates for an I = 5/2 nucleus (such as ^{17}O) in a magnetic field, with central and satellite transitions (CT and ST, respectively) indicated.

the isotropic resonances from these ST transitions give rise to shoulders to the CT resonances at low and high frequencies, respectively for the m_I = 3/2-½ and 5/2-3/2, transitions respectively. By changing the pulse length, the degree to which the CT and STs are excited can be changed [Figure S3] and corresponding text), resulting in a change to the lineshape, from which the strength of the quadrupolar interaction may be modelled.

The strong quadrupolar interaction also results in a nutation profile (the variation in signal intensity with length of the applied radiofrequency pulse used to excite the signal) that differs depending on the size of the quadrupolar interaction relative to the size of the radio frequency field used to excited the spins. Consequently, shorter pulse lengths are required (compared to a non-quadrupolar nucleus) to ensure the spectra are quantitative for all sites, regardless of the size of C_Q.[3] Briefly, when the quadrupolar interaction is strong, the applied radiofrequency pulse inefficiently excites the satellite transitions (ST, Figure S2(b)), and the pulse selectively excites the central transition (CT, Figure S2(b)); this is known as the selective limit. In this case, the pulse length must be shortened by a factor of (I+½)—e.g., where a $\frac{\pi}{2}$ pulse would be used for a non-quadrupolar nucleus, a $\frac{\pi}{6}$ pulse is needed for quadrupolar ^{17}O [Figure S3] to obtain the maximum signal for the CT; this 'maximum' is only one third of the intensity obtained when using a selective pulse, however; the equations for non-selective and

selective pulses are given in eqs. (S-1) and (S-2), respectively. As different sites experience different quadrupolar interaction strengths, each site may have a different nutation curve and require a different pulse length to achieve the same flip angle. One must carefully consider whether optimising the pulse length on a sample which has ^{17}O with a smaller C_Q is appropriate when studying a sample whose ^{17}O environments are strongly quadrupolar. To obtain quantitative signals of quadrupolar nuclei, very short flip angles are generally used in one-pulse experiments, where the signals obtained for selective and non-selective excitation are essentially identical; it is however challenging to obtain quantitative spectra when echo pulse sequences are used.

$$S(\tau_\mathrm{p}) = S_0 \sin(\omega_1 \tau_\mathrm{p}), \tag{S-1}$$

$$S(\tau_\mathrm{p}) = \frac{S_0}{I + \frac{1}{2}} \sin\left(\left(I + \frac{1}{2}\right)\omega_1 \tau_\mathrm{p}\right), \tag{S-2}$$

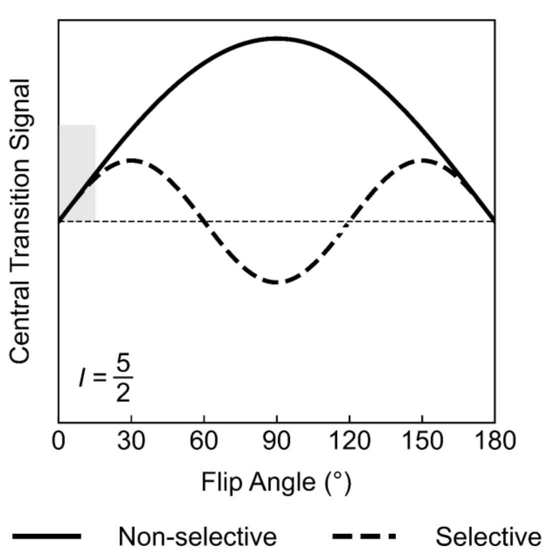

Figure S3: A schematic nutation profile for selective and non-selective radiofrequency pulses, again for an I = 5/2 nucleus. The range of flip angles close to quantitative excitation is illustrated by the shaded [illegible]

To obtain quantitative information about the quadrupolar interaction, it is recommended that spectra are acquired at different field strengths and/or with different pulse lengths. By carefully fitting the experimental spectra (with either different applied fields or different pulse lengths), one can extract a value of C_Q, which in turn may be used to assign the spectrum, based on how ionic or covalent a site is anticipated to be. It should be noted, however, that the changes in both quadrupolar and hyperfine interaction (as a result of field or pulse length changes) may make it challenging to separate the contributions of each interaction to the changes seen in the spectrum. Both the hyperfine and quadrupolar interactions contribute to the breadth of the resonance(s) observed in a spectrum; of these, the short relaxation time (transverse, T_2) imposed by the hyperfine interaction dominates in Li*TM*O_2 systems. The size of the hyperfine interaction is typically on the order of 10^1 to 10^2 MHz, whilst that of the quadrupolar interaction is limited to < 10 MHz.

3. Calculation of ^{17}O NMR Parameters

Calculations on LIB cathode systems require DFT methods such as Hubbard-corrected DFT+*U* or hybrid functionals. That said, it is unclear whether a single Hubbard *U* parameter may be used to model all *TM* sites in a material (due to differences in the local chemical environments and densities of states expected), and whether the *U* determined for a transition metal is relevant when considering spin density transfer to the oxygen ion. Whilst localised hybrid DFT atomic basis sets may be used, it is also unclear to what extent these are influenced by finite cell effects and interactions between localised polarons.[8]

A calculation of the isotropic hyperfine shift requires a determination of the isotropic hyperfine coupling constant:[2,9]

$$A_{\text{iso}} = \frac{\mu_0 \mu_{\text{B}} \mu_{\text{N}} g_{\text{e}} g_{\text{N}} \left|\psi^{\alpha-\beta}(\boldsymbol{R}_{\text{N}})\right|^2}{3S}, \tag{S-3}$$

where μ_0 is the permittivity of free space; μ_{B} the Bohr magneton; μ_{N} the nuclear magneton; g_{e} and g_{N} the free-electron and nuclear *g*-factors, respectively; $\left|\psi^{\alpha-\beta}(\boldsymbol{R}_{\text{N}})\right|^2$ is the unpaired spin density at the nuclear position and S is the total electronic spin. This last quantity is calculable; from this, A_{iso} may be determined and hence the isotropic Fermi contact shift, δ_{FC}, can be found:

$$\delta_{\text{FC}} = \frac{2SA_{\text{iso}}\chi_M}{N_{\text{A}} \mu_0 \mu_{\text{B}} \mu_{\text{N}} g_{\text{e}} g_{\text{N}}}, \tag{S-4}$$

where χ_M is the molar magnetic susceptibility of the material and N_{A} is Avogadro's number. As δ_{FC} depends on the magnetic susceptibility, it is possible to extract information about the paramagnetic behaviour of the material from the paramagnetic shift.

The values of $\left|\psi^{\alpha-\beta}(\boldsymbol{R}_{\text{N}})\right|^2$ are determined *via* a single-point energy calculation of a fictitious ferromagnetic ground state—i.e., the electron spins on all *TM* centres are fixed to be parallel. Whilst this magnetic state is not (in general) a true description of the $T = 0$ K magnetic structure of the material, this state is more convenient for DFT calculations. To ensure good convergence of this state and ensure the spin densities are determined accurately, the calculations proceed *via* the following steps:

1. Perform a geometry optimisation using 'contracted' basis sets (i.e., bases which contain the bare minimum number of functions required to describe the orbitals) when the spins of all *TM* ions are fixed parallel to each other (a 'spin-locked' state);

2. Re-optimise the structure from step (1), this time starting from the 'spin-locked' converged wavefunctions, without any spin constraints in place;

3. Using the optimised structure, begin a single-point energy calculation using a set of 'extended' basis sets (i.e., bases which describe the electronic structure of the ions accurately, by including unoccupied valence orbitals in its description) with all *TM* spins fixed parallel to each other;

4. Run a final single-point energy calculation on the converged wavefunction from step (3), this time removing the constraints on the relative spin orientations.

4. Origin of Broadening of ^{17}O NMR Spectra for NCA

The Co^{4+} centres generated at the end of charge in NCA induce rapid relaxation and any resonances arising from environments with pathways to Co^{4+} may be severely broadened, analogous to the disappearance of the ^{7}Li NMR signal in $LiCoO_2$ on charging.[56] As low-spin Co^{4+} is a t_{2g}^{5} ion, its degenerate ground state has residual orbital angular momentum potentially introducing further broadening mechanisms. Co^{4+} can also undergo a JT distortion to give a singlet state. Unlike an e_g-driven JT distortion, the t_{2g} JT is weaker, and thus likely to be a dynamic rather than static JT (at least at ambient temperatures). If the JT fluctuations are on the timescale of the hyperfine interaction, this is likely to result in a severe broadening analogous to the intermediate regime of site exchange.[55] The additional effect of Li^+ ion hopping and dynamic JT distortions on the ^{17}O NMR spectrum in the presence of a system with ground states of non-zero L is unclear, but may also contribute to the observed breadth of the spectrum. While more detailed electron paramagnetic resonance (EPR) studies are required to understand this phenomenon, when combined with the dilution of Co^{4+} centres by the Ni and Al ions in the TMO_2 layers—resulting in fewer, weaker electron-electron dipolar interactions—this likely results in a lengthened T_{1e} and thus a faster nuclear relaxation time,[45] giving severely broadened signals.

5. Anomalous "Negative Intensity" in ^{17}O NMR Spectra of Li_xCoO_2

In the main text, Geng *et al.* proposed that the negative intensity seen at 378 ppm arises from left-shifting the FID before Fourier transformation (necessary to eliminate baseline distortions), but it may also be due to differences in the effective pulse length felt by ZrO_2 due to offset effects (which result in different effective nutation frequencies[10]). Differences in the C_Qs of the different sites may also result in different effective pulse lengths. However, the C_Qs of pristine LCO and ZrO_2 are not substantially different: approximately 1.47 MHz and 1.0 MHz, respectively,[11,12] so while this effect may be important at higher states of charge (see below) this is a less likely source of this phase shift. The change in phase of the ZrO_2 peak at the spectra obtained at points C8 and C9 likely arises from a change in the receiver offset position at these states of charge.

6. Oxygen Redox Mechanisms

At present, there is no consensus on whether oxidised O states form, and if so, how they may be stabilised. Different stabilisation mechanisms for O redox have been proposed, based on data collected from synchrotron diffraction,[3] transmission electron microscopy (TEM),[13,14] X-ray photoelectron spectroscopy (XPS),[15] X-ray absorbance spectroscopy (XAS) and resonant inelastic X-ray scattering (RIXS)[16–21] experiments and *ab initio* calculations.[22–24] The proposed mechanisms include: the formation of peroxo-like (O_2^{n-}) species;[13,25,26] molecular oxygen trapping;[19,20,27] localisation of holes onto O;[28–30] *TM* migration[31–33] and π redox.[24] Each of these mechanisms are described below.

6.1 Peroxo Formation

One of the proposed mechanisms for the stabilisation of oxidised O is the formation of peroxo-like species, $(O_2)^{n-}$. It has been proposed that, when removing electrons from non-bonding O states, the O sublattice can distort by moving pairs of O centres close in space, leading to a bonding interaction between the partially depopulated non-bonding O states and the formation of peroxo-like O–O dimers [Figure S4 (a) and (b)].[14,22,34]

To examine this mechanism, 'model' compounds, such as Li_2IrO_3 and Na_2RuO_3,[13,35] were studied.[a] Although layered cathodes containing 4*d* and 5*d* *TM* cations are less commercially viable than 3*d*-based cathodes, the highly covalent *TM*–O interactions ensure that high-energy O-based states are generated, which promotes O redox reactions. In both compounds, a large increase in capacity was observed at charging voltages beyond the conventional ($Ir^{4+/5+}$ and $Ru^{4+/5+}$) redox couples. It was suggested that, the *TM*–O bonds shorten during charge, causing the O^{2-} ligands to move closer together, eventually leading to orbital overlap to generate O–O σ bonds.[13,35,36] As a result, partially occupied, high-energy O–O σ^* states are generated, from which electrons may be removed. Stabilisation was therefore proposed to arise from the spreading of electron density over the O–O dimer, as opposed to localisation onto single O centres.

For Li_2IrO_3, TEM was used to observe the IrO_6 coordination polyhedra, revealing three short O–O distances and three long O–O distances, suggesting the formation of $(O_2)^{n-}$ [Figure S4(c) and (d)]. It was claimed that the long O–O bond length observed (compared to a typical peroxo species) indicates weak O–O bonding, due to O oxidation.[13] Furthermore, *ab initio* density functional theory (DFT) electronic structure calculations indicated that $(O_2)^{n-}$ species may be involved in the high-voltage redox couple.[13]

A subsequent study investigated the two polymorphs of Na_2RuO_3, where Na and Ru are either ordered or disordered across the metal sublattice in the TMO_2 layers.[35] Whilst the ordered polymorph exhibited a high-voltage redox couple—attributed to O oxidation—the disordered polymorph did not, but instead released O_2 during charging. With the support of electronic structure calculations, it was proposed that whilst the ordered polymorph was able to accommodate the strains induced by the formation of $(O_2)^{n-}$ species, the disordered polymorph was not; this inhibits $(O_2)^{n-}$ formation and prevents stable O redox.[35]

[a] These materials can also be described as $Li[Li_{1/3}Ir_{2/3}]O_2$ and $Na[Na_{1/3}Ru_{2/3}]O_2$.

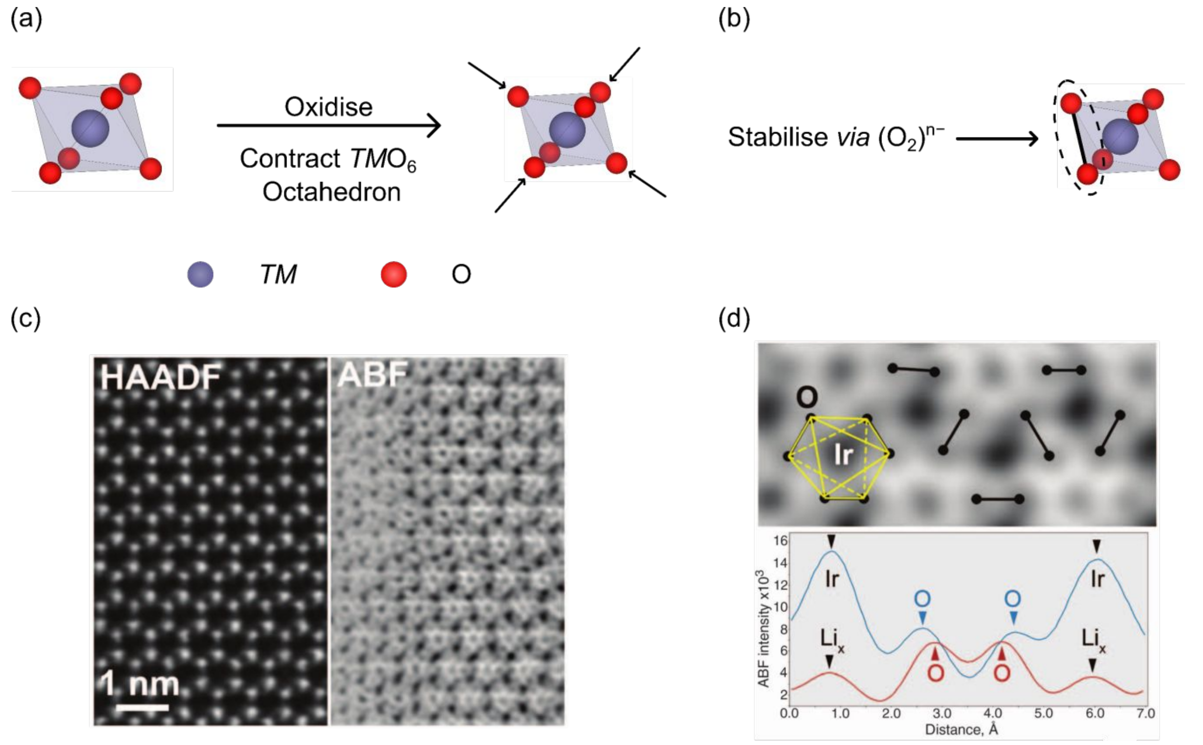

Figure S4: Peroxo-formation mechanism and TEM evidence. **(a)** shows a schematic of the contractive effect of oxidation on the TMO_6 octahedra in $Na_xM_yTM_{1-y}O_2$, whilst **(b)** indicates how peroxo $(O_2)^{n-}$ species can form around the TMO_6 octahedron. **(c)** and **(d)** show TEM high-angle annular dark field (HAADF) and annular bright field (ABF) images of Li_2IrO_3, with the short and long O–O distances highlighted in **(d)**. In the HAADF images, Ir atoms are white, whilst O are not seen; in the ABF images, Ir and O appear dark. **(c)** and **(d)** are adapted with permission from ref. 13. Copyright 2015 American Association for the Advancement of Science.

6.2 O_2 Trapping

A related mechanism for the stabilisation of oxidised O species is the formation and trapping of molecular O_2 inside the lattice of the layered cathode material. This mechanism has been proposed for Li-rich layered cathodes, as well as Li-doped, Mn-based NIB cathodes. It is suggested that, as O^{2-} is oxidised, it spontaneously disproportionates into O^{2-} and O^0—i.e., $2O^- \rightarrow O^{2-} + O^0$; when repeated, two O^0 species may combine to form O_2. At the surface of the cathode particle these O_2 species may readily desorb and are released into the electrolyte, but in the bulk of the particles, it is hypothesised that these O_2 units are 'rigidly contained' within the lattice by clustering around vacancies in the TMO_2 layers.[20] These vacancies are generated by the loss of Li^+ from the TMO_2 layers, either by deintercalation (for the Li-rich cathodes), or by Li^+ migration into vacant tetrahedral sites in the Na^+ O-type layers (for the Li-doped, Mn-based NIB cathodes). Subsequent migration of *TM* cations within the TMO_2 layers can then generate clusters of vacancies, which can trap the O_2 generated [Figure S5(a)]. It is also hypothesised that the O redox behaviour of cathodes can therefore be tuned by modifying the in-plane ordering of *TM* cations over the TMO_2 layers—for example, honeycomb ordering of vacancies encourages O_2 species to form and become stabilised, whilst "ribbon ordering" over the TMO_2 layers encourages localised O holes to form.[19]

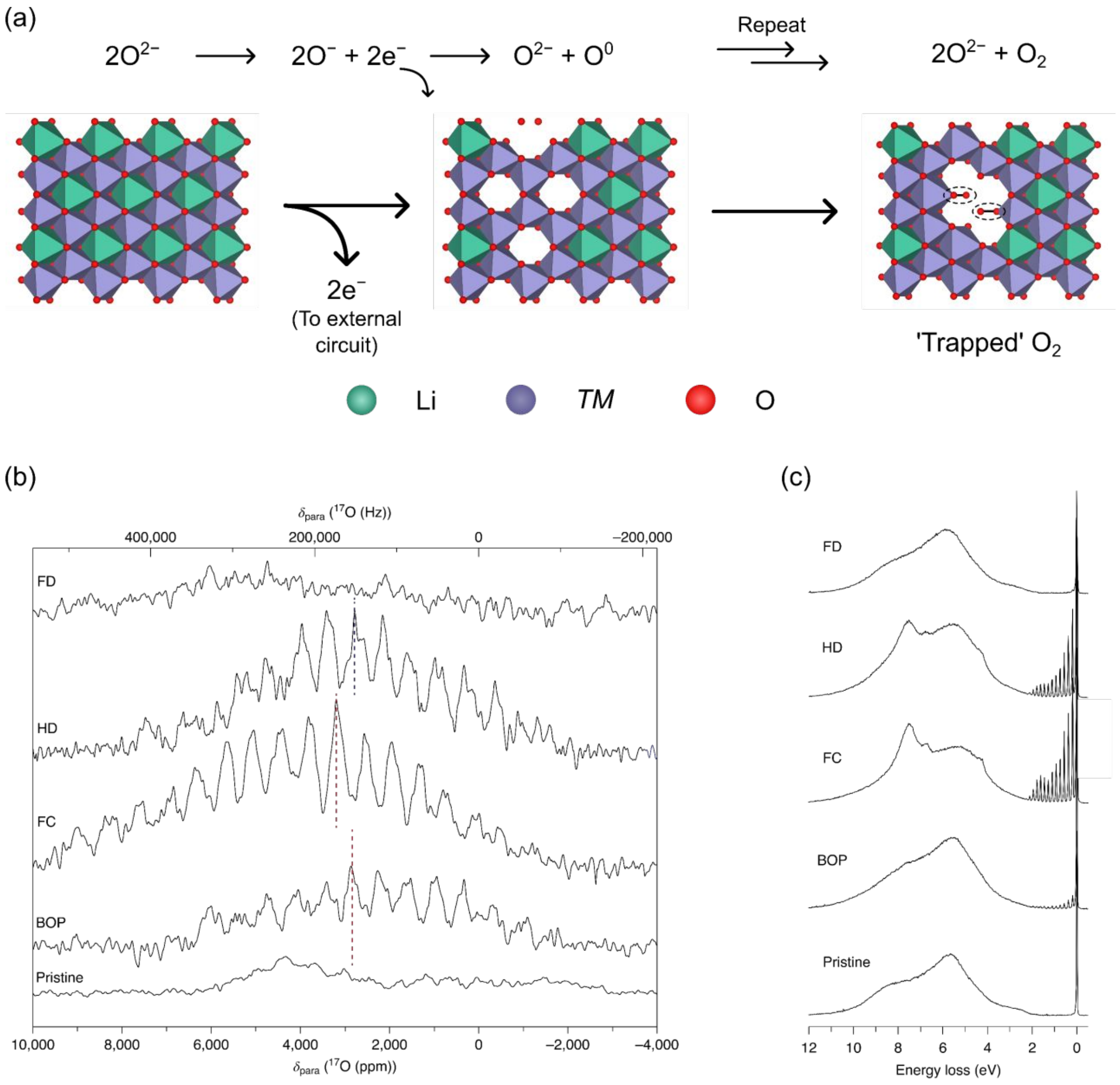

Figure S5: O_2-trapping stabilisation mechanism for O redox. **(a)** shows a schematic of the O_2 trapping mechanism in a honeycomb-ordered Li-rich layered cathode, with views onto the TMO_2 plane at each stage during the mechanism shown below. **(b)** shows ^{17}O NMR spectra recorded for $Li_{1.2}Ni_{0.13}Co_{0.13}Mn_{0.54}O_2$ in the pristine, beginning of plateau (BOP), end of first charge (FC), half-discharged (HD) and end of first discharge (FD) states; each spectrum was recorded at 9.45 T and a magic angle spinning frequency of 34 kHz. **(c)** shows the O *K*-edge RIXS spectra for $Li_{1.2}Ni_{0.13}Co_{0.13}Mn_{0.54}O_2$ at each state of charge **(b)** and **(c)** were adapted with permission from ref. 20.
Copyright 2020 Nature-Springer group.

Two studies have proposed the O_2 trapping mechanism: the first using $Na_{0.6}[Li_{0.2}Mn_{0.8}]O_2$ and $Na_{0.75}[Li_{0.25}Mn_{0.75}]O_2$[19] and the second using $Li_{1.2}Ni_{0.13}Co_{0.13}Mn_{0.54}O_2$.[20] In the first study, the O redox reactions of $Na_{0.6}[Li_{0.2}Mn_{0.8}]O_2$ and $Na_{0.75}[Li_{0.25}Mn_{0.75}]O_2$ were compared;[19] the former exhibits 'ribbon ordering' of Li and Mn over the TMO_2 layers, whilst the latter shows a honeycomb superstructure. During charge, the Li^+ ions in both materials migrate from the TMO_2 layer into adjacent desodiated Na^+ layers; on discharge, Li^+ migrates back to the TMO_2 layer. For ribbon-ordered $Na_{0.6}[Li_{0.2}Mn_{0.8}]O_2$, the superstructure was retained over the charge-discharge cycle, but the honeycomb ordering in $Na_{0.75}[Li_{0.25}Mn_{0.75}]O_2$ was lost. Similarly, the honeycomb ordering of *TM* cations in $Li_{1.2}Ni_{0.13}Co_{0.13}Mn_{0.54}O_2$ was also lost on charging.

To investigate the origin of this disordering process, DFT calculations of the energies of different TM/vacancy configurations in the TMO_2 layers were run: these revealed that the loss of honeycomb ordering arose from *TM* migration and clustering of vacancies. This clustering

generated O centres which were no longer coordinated by Mn and dimerised to form O–O species, resulting in a lower total energy.

To further investigate the nature of oxidised O species in $Li_{1.2}Ni_{0.13}Co_{0.13}Mn_{0.54}O_2$, ^{17}O nuclear magnetic resonance (NMR) spectroscopy was used: here, the authors identified a new, paramagnetically-shifted signal which appeared towards the end of charge and assigned this new resonance to trapped O_2 [Figure S5].[20] The authors also used RIXS at the O *K*-edge to probe the valence electronic states of O at high states of charge in all three cathode materials, and to search for evidence of O–O species. For each material at the end of charge, in addition to the inelastic region of the spectrum (which reveals information about the valence electronic states on O), the authors noted the appearance of a progression of energy loss peaks about the elastic peak, whose spacings were consistent with the energy of vibration of an O–O bond and resembled the RIXS spectrum for molecular O_2 [Figure S5]. The authors also highlighted that the O–O dimer formed in $Na_{0.75}[Li_{0.25}Mn_{0.75}]O_2$ has a longer bond length than peroxo-like species and remains trapped in the lattice, as it cannot diffuse to the surface of the cathode particles and escape.

Ribbon-ordered $Na_{0.6}[Li_{0.2}Mn_{0.8}]O_2$ also showed the O_2-like progression feature, but with a much lower intensity, suggesting that much less trapped O_2 is formed in this material. Instead, localised holes on O were identified using *ex situ* soft X-ray absorption spectroscopy (SXAS) at the O *K*-edge, suggesting that dimerization of oxidised O species does not occur in $Na_{0.6}[Li_{0.2}Mn_{0.8}]O_2$. The reason for the lack of dimerization proposed by the authors was that the ribbon ordering of TM cations suppressed intralayer Mn migration (compared to the honeycomb-ordered case). As a result, O_2 species could not be stabilised, so the driving force to disproportionate O^- species decreases.

6.3 Hole Localisation

Unlike 4*d* and 5*d* 'model' compounds, some 3*d* TM-based cathode materials known to exhibit an increase in capacity at high voltages show no $(O_2)^{n-}$ formation at high states of charge.[30] For example, recent *ex situ* Raman spectroscopy studies of Li-rich nickel manganese cobaltates (NMCs) showed that O–O bonds were absent at high potentials.[29,30] Instead, localised holes on O were identified at high potentials using SXAS and RIXS [Figure S6]. The authors suggested that hole states only form on O coordinated by Mn^{4+} and Li^+, due to the highly ionic nature of these interactions.

Whilst the localisation of holes on O and the formation of peroxo- or O_2-like species appear as different mechanisms, a recent investigation identified the conditions under which holes may condense to form $(O_2)^{n-}$ species, or remain localised.[28] Here, the authors argued that peroxo species may form provided that neighbouring oxidised O species can rotate to undergo orbital overlap without sacrificing the overlap between O and nearby TM cations. This corresponds to situations where O is covalently bonded to as few cations as possible (ideally, none). The pairing and localisation of oxidised O centres for different TM arrangements in the TMO_2 layer is also consistent with the O_2 trapping mechanism.

6.4 *TM* and *M* Migration Induced Oxygen Oxidation

In addition to having high capacities, most O-redox-active cathodes also show a large difference in the charge and discharge potentials of the cathode (known as *voltage hysteresis*),

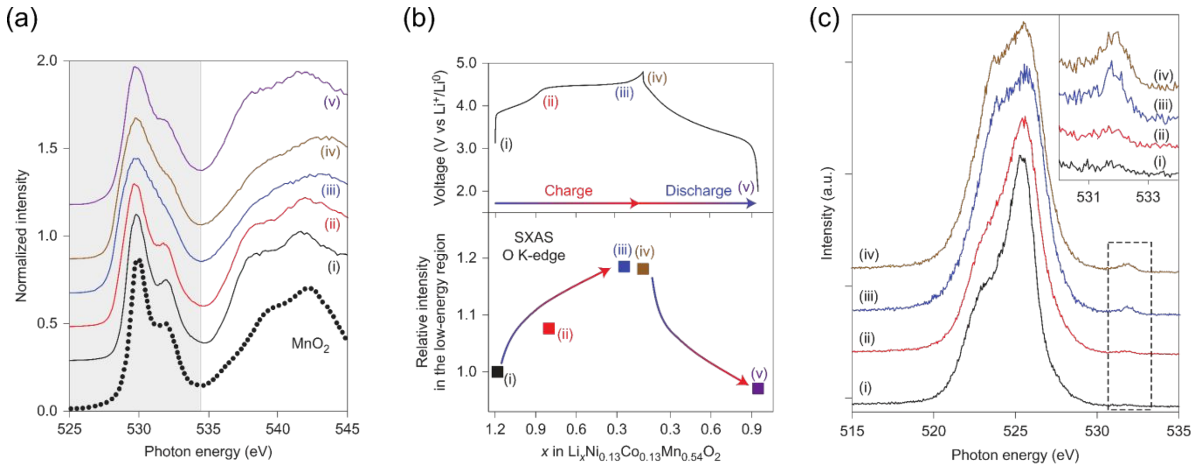

Figure S6: Evidence of localised O holes in $Li_xNi_{0.13}Co_{0.13}Mn_{0.54}O_2$. **(a)** shows the O *K*-edge SXAS data recorded at different states of charge, with the low-energy region of the spectra highlighted to show the gradual appearance of a new shoulder peak whose energy was identified with hole states on O. **(b)** shows the electrochemical profile for $Li_xNi_{0.13}Co_{0.13}Mn_{0.54}O_2$ during the first charge-discharge cycle (top) and the change in the intensity of the low-energy region of the SXAS data. **(c)** shows the change in the RIXS spectra at different states of charge in $Li_xNi_{0.13}Co_{0.13}Mn_{0.54}O_2$, with the low-intensity peak at approximately 532 eV (assigned to O hole states) highlighted in the inset. Reprinted by permission from Nature Chemistry, ref. 29, copyright 2016.

as well as large overpotentials throughout the high-voltage region of the voltage profile (i.e., the region where O redox is proposed to take place). These electrochemical features suggest that O redox is coupled with a dynamic process whose activation energy barrier is large and/or strongly asymmetric.[b, 31–33] The asymmetry of the energy barrier is proposed to cause the voltage hysteresis and high overpotentials seen. Several authors believe that this dynamic process is the migration of *TM* or *M* cations from their octahedral sites in the TMO_2 layer to tetrahedral sites in the Na^+ (or Li^+) layer—known as interlayer migration—or migration of *TM* cations to *TM* or *M* vacancies within the TMO_2 layer, known as interlayer migration. The migration process is believed to stabilise oxidised O species and facilitate O redox—i.e., $\{O^{2-} + TM\} \rightarrow \{O^{-} + TM_{mig.} + e^{-}\}$.[31] A variant on this mechanism instead suggests that the migration of *TM* cations into tetrahedral sites in the Li^+ or Na^+ layers enables high oxidation state *TM* cations to form—i.e., O redox does not occur at all.[23]

[b] An asymmetric energy profile indicates a large difference between the rates at which the 'forward' and 'reverse' processes take place. As a result, whilst the forward reaction may occur quickly, the reverse may need a large driving force (electrochemically: a larger overpotential is required to achieve the same Li^+ or Na^+ content on charge and discharge).

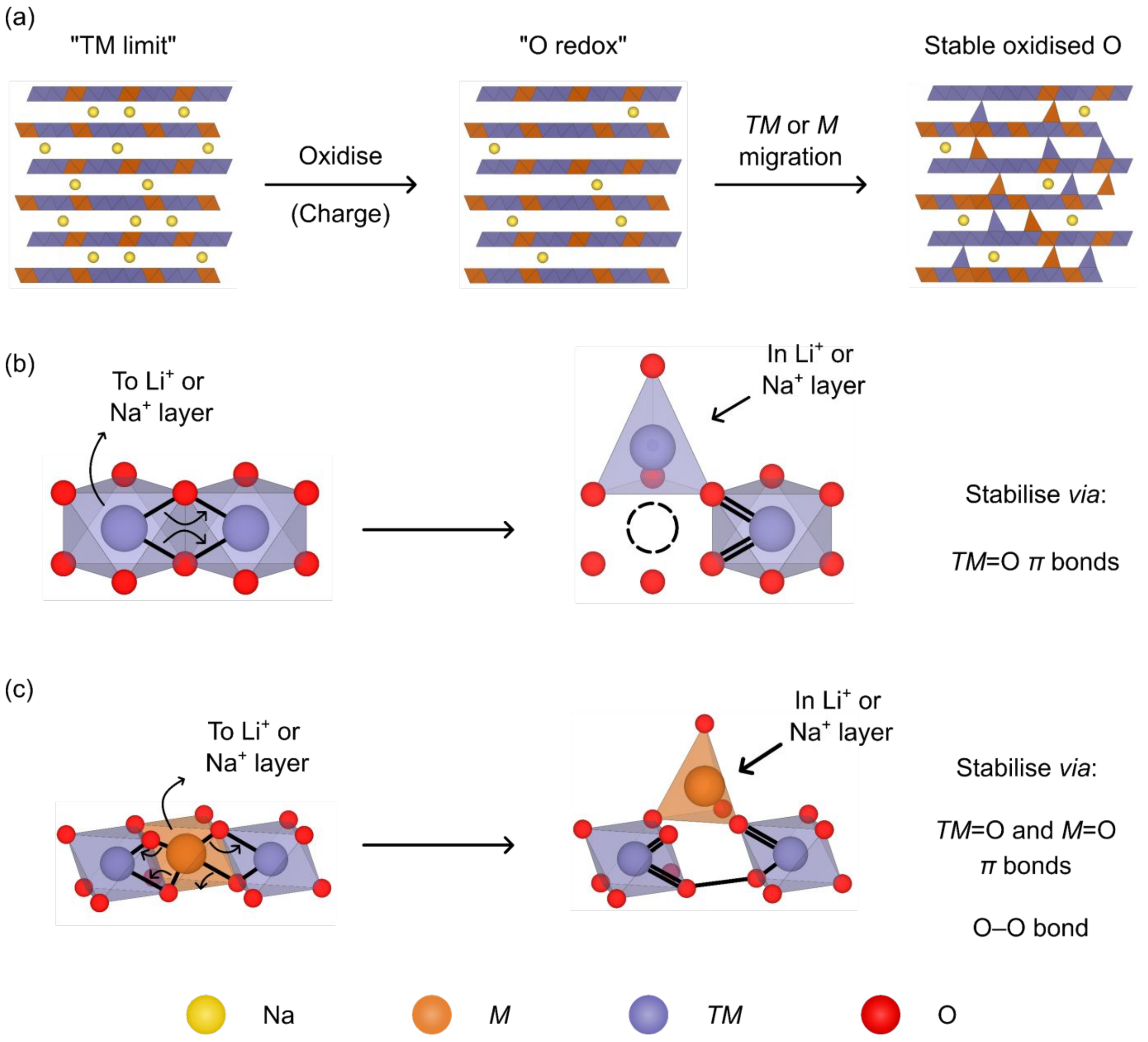

Figure S7: Schematics showing the *TM* migration mechanism for oxidised O stabilisation. **(a)** shows an O2-type $Na_xM_yTM_{1-y}O_2$ cathode at the limit of *TM* redox (far left), which may be oxidised (charged) *via* O redox to extract more Na^+ (centre). It is hypothesised that the system may then be stabilised *via* *TM* or *M* migration (far right). **(b)** and **(c)** show the changes in local environment when the **(b)** *TM* and **(c)** *M* cations migrate. The *TM*–O and *M*–O bonds which change during the mechanism are highlighted in black, with curly arrows indicating the flow of electron pairs.

The case of intralayer migration was mentioned earlier, where it was suggested that *TM* migration enabled underbonded O species to dimerise and form O_2-like species, ultimately stabilising oxidised O. This mechanism was further explored in a Li-rich nickel manganese cobaltate (NMC), $Li_{1.17}Ni_{0.21}Co_{0.08}Mn_{0.54}O_2$, where the authors used synchrotron X-ray diffraction (SXRD), scanning transmission X-ray microscopy (STXM), XAS and RIXS to show that *TM* migration occurs on charging to high voltages.[32] The SXRD patterns collected revealed a loss of the *TM* ordering present in the pristine material, indicating intra-layer migration of the *TM* cations, whilst STXM and XAS revealed that O 2*p* states were depopulated at high charging voltages (above 4.35 V *vs* $Li^{0/+}$). It was also revealed that the non-bonding O 2*p* states appeared at energies *above* the (now vacant) *TM*–O states. This was interpreted as a 'reshuffling' of the electronic states during charge.[32] On further charging, the O 2*p* states were depopulated more and moved to increasingly higher energies; on discharge, these states were not refilled until lower voltages (compared to the voltages used

to remove electrons from these states during charge), consistent with the proposed reshuffling. Critically, the reshuffling of states seen in XAS and the *TM* migration seen in SXRD were strongly correlated; hybrid DFT calculations revealed that, as the *TM* cations migrated within the TMO_2 layers, the redox-active O 2*p* states shifted to higher energies, with states based on O bound to Mn increasing the most in energy.

Interlayer migration was been proposed to occur in the family of model compounds $Li_2Ir_{1-y}Sn_yO_3$, where *TM* migration was correlated with O redox activity.[31] In this work, Ir L_3-edge and O *K*-edge XAS was employed to show that the high-voltage capacity in Li_2IrO_3 arose from Ir oxidation only (up to $Ir^{5.5+}$), whilst the high-voltage capacity seen for the Sn-substituted material arose from Ir oxidation up to $Ir^{5.5+}$, followed by O oxidation. Furthermore, SXRD revealed that Sn migration occurs when charged beyond $Ir^{5.5+}$, suggesting that O redox activity is modulated by Sn migration. Hybrid DFT calculations showed that the migration of Sn and concomitant formation of a Sn vacancy in the TMO_2 layer stabilised the delithiated structure, which was attributed to a transformation of the 'dangling' *TM*–O bonds (formed when Li^+ ions are removed from the TMO_2 layers). The authors suggested that the fate of these dangling bonds depends on the cation identity: for Ir, the newly-generated non-bonding O 2*p* states rehybridize to form Ir−O π bonds; for Sn, O–O dimers form to straddle the vacancy left behind by Sn migration [Figure S7(b) and (c), respectively]. Both scenarios result in the non-bonding O 2*p* states becoming higher in energy (i.e., available for redox) whilst ensuring that O does not become under-bonded and unstable. As a result, O redox can occur stably. For undoped Li_2IrO_3, O redox was not observed (contrary to previous studies); this was attributed to the lack of Sn cations, which were deemed to encourage migration.

6.5 π Redox

Whilst it was stated earlier that O redox materials are plagued with voltage hysteresis and irreversible structural changes, two layered cathodes, $Na_2Mn_3O_7$ and Li_2IrO_3,[13,37–39] both known to exhibit anomalously high capacities, show neither of these effects during cycling. The proposed charge compensation mechanism for these materials is known as π redox.

A recent computational study proposed that the high-voltage redox couples in these materials arise from oxidation of a delocalised π system generated from overlap of the *TM d* orbitals and O *p* orbitals [Figure S8(a)] arranged in a ring of six *TM* cations.[24] These TM_6 rings are seen throughout O redox active cathodes, where honeycomb ordering of the *TM* and *M* cations over the *TM* sublattice occurs.[19,27,35] Crucially, the redox-active π states have contributions from both O and Mn, rather than from predominantly O-based orbitals, meaning that the origin of the high-voltage capacity arises from redox reactions involving both Mn and O, rather than O alone. It was then shown that the formation of this π system inhibited *TM* migration and O de-coordination, meaning that these cathodes undergo little structural changes during charge and discharge—i.e., the voltage hysteresis in these materials is significantly smaller than for other O-redox-active cathodes, as observed experimentally.[13,37,40]

The authors further proposed that, as well as preserving the structure of the cathode during charge and discharge, the π system formed accounts for the apparent formation of O–O dimers and partial charge transfer to nearby *TM* cations. The authors claim that, on oxidising the *TM*–O states, the *TM*–O bonds shorten, resulting in an expansion of the π system; if adjacent π systems expand simultaneously, pairs of O centres move closer together, giving the shortened O–O distances which other studies[4] interpreted as peroxo-like O–O dimers

[Figure S8(b)]. The charge transfer process is proposed to be an artefact of the redistribution of electron density over the oxidised π system.

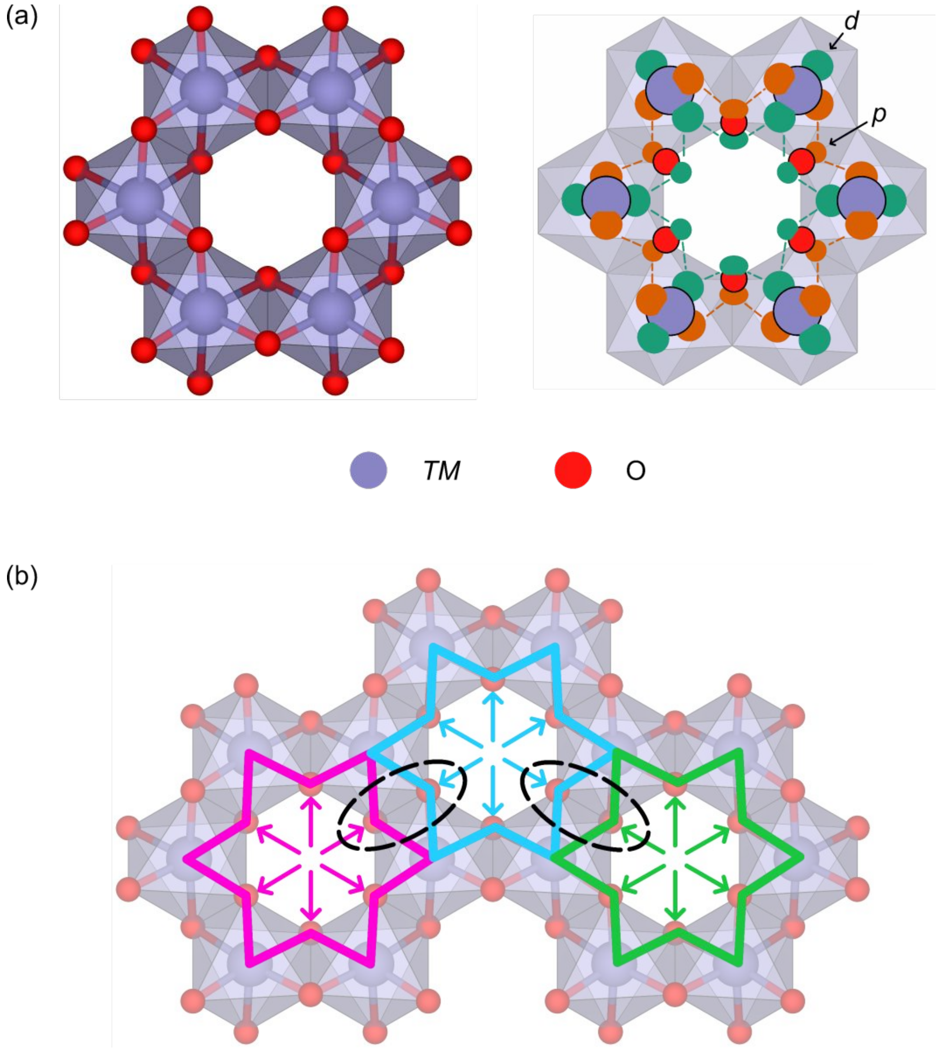

Figure S8: Schematics showing the mechanism and structural consequences of π redox. **(a)** shows the TM_6 rings commonly seen in O-redox-active cathodes (left), with the orbital overlap between *TM d* orbitals and O *p* orbitals (right); positive and negative lobes of the orbitals are shown in orange and green, respectively. **(b)** shows the effect of 'expanding' the π-bonded ring by shortening the *TM*–O bonds – this expansion generates shortened O–O distances between the TM_6 rings, giving the appearance of peroxo-like O–O dimer species. Adapted with permission from ref. 24. Copyright 2021 American Chemical Society.

7. Pulse Sequences Used in ^{17}O NMR

Owing to the strong electron-nuclear hyperfine and quadrupolar interactions at the ^{17}O centres in Li_xTMO_2 materials, the spin-spin relaxation time, T_2, can be significantly shortened. To counteract this, Geng *et al.* used a single-pulse experiment—i.e., $\pi/2$–acquire—to examine the ^{17}O environments in LCO. Whilst excitement of the pristine spectrum is likely efficient—the fitted C_Q the authors obtained for pristine LCO (1.47 MHz) corresponds to under 10% of the nuclear Zeeman splitting, so this coupling may be considered as "weak",[4] meaning the second-order quadrupolar effect is small and the STs are inefficiently excited by the $\pi/2$ pulse—this may not be the case for the charged samples. For delithiated LCO, the large C_Qs (7.22–7.98 MHz) exceed 10% of the nuclear Zeeman splitting, such that second-order quadrupolar coupling is significant and STs are no longer inefficiently excited by $\pi/2$. In these cases, the use of a $\pi/6$ pulse may be more appropriate to excite the spectra. Furthermore, a Hahn-echo could be used to refocus the second-order quadrupolar interaction, if the T_2s of the local environments permit.

In contrast, when studying $Li_{1.2}Ti_{0.4}Mn_{0.4}O_2$ (LTMO), Geng *et al.* employed a solid echo pulse sequence this differs from a typical Hahn-echo in that the second pulse is a $\pi/2$ pulse applied about the *y* axis of magnetisation, rather than a π pulse applied about *x* (i.e., the solid echo sequence is $\pi/2_x$–τ–$\pi/2_y$–τ–acquire, with τ rotor-synchronized). This pulse sequence is used to refocus spins that have evolved under quadrupolar or homonuclear interactions in static samples.[4] Some authors use it for quadrupolar systems, even under MAS (where the first order quadrupolar interaction is already refocussed by MAS), possibly because, in practice, it may provide more efficient refocussing of the signal, because of the more efficient excitation and broad-band nature of a shorter pulse. The second-order quadrupolar coupling is refocussed by π pulses (but not by MAS), however, suggesting that a standard Hahn-echo may be more suitable for this system.

When studying the lithium-rich nickel manganese cobaltate (NMC) compound, $Li_{1.2}Ni_{0.13}Co_{0.13}Mn_{0.54}O_2$, House *et al* used a short-delay pre-saturation echo pulse sequence to record the ^{17}O NMR spectra; here, a series of $\pi/2$ pulses is applied and then a Hahn-echo with short delays (29.4 μs, so that the pulse sequence is rotor-synchronised) between the pulses is recorded [Figure S9].[20] By pre-saturating the resonance and using short delays between pulses, only the species which relax rapidly (i.e., those species with short longitudinal relaxation times, T_1) can be observed.

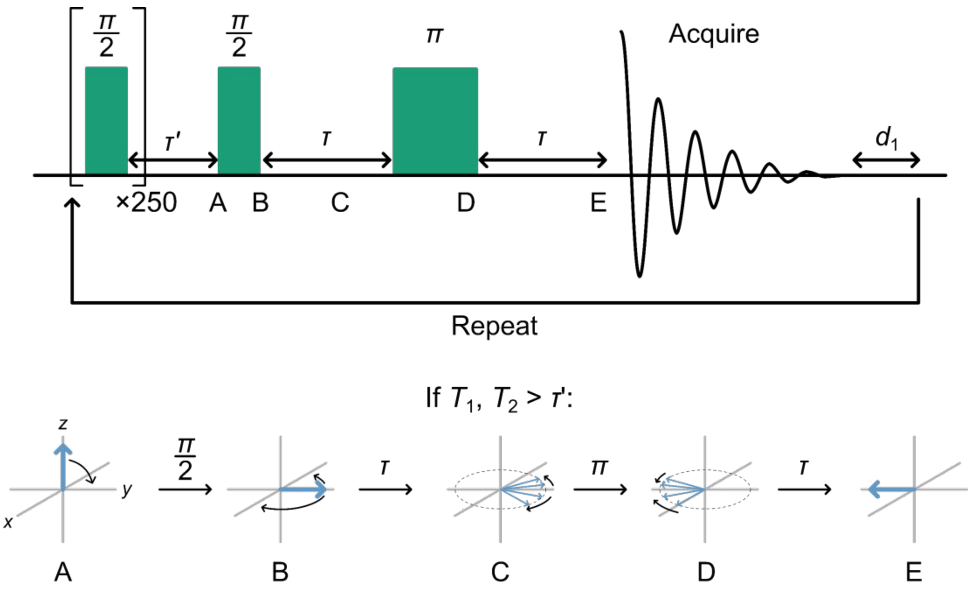

Figure S9: Schematics showing the pre-saturation Hahn-echo pulse sequence used for ^{17}O NMR experiments on $Li_{1.2}Ni_{0.13}Co_{0.13}Mn_{0.4}O_2$ alongside the corresponding vector diagram of nuclear magnetisation.

Finally, to perform *in situ* ^{17}O NMR experiments on Li_2MnO_3, Li *et al.* used the quadrupolar Carr-Purcell-Meiboom-Gill (qCPMG) to increase sensitivity by increasing signal-to-noise. Briefly, the qCPMG pulse sequence initially records a Hahn-echo, then applies a refocussing π pulse and records another echo; repetition of the π-acq section of the pulse sequence generates a series of free induction decays (recorded in the time domain), which when Fourier transformed become a set of "spikelets" in the frequency domain whose spacing is the reciprocal of the time separation between each π-acq step and whose intensity envelope describes the static spectrum lineshape.[41,42] This pulse sequence effectively increases sensitivity by concentrating the spectral intensity in each spikelet, rather than spreading it over the entire frequency span (this effectively increases signal-to-noise); it relies on having a sufficiently long T_2 such that the transverse magnetisation does not decay between the π-acq steps. It is unclear whether the T_2 of ^{17}O in Li_2MnO_3 is sufficiently long to enable quantitative acquisition of all resonances. Due to experimental constraints, MAS is not possible in *in situ* experiments, so the resonances remain severely broadened by the hyperfine interaction (in a manner akin to the *in situ* ^7Li resonances in $LiNi_{0.8}Mn_{0.1}Co_{0.1}O_2$[43]); the authors nevertheless analysed these spectra. They also highlight that whilst the cell is not optimised in terms of the capacity extracted, the results obtained are nevertheless illustrative of the charge-discharge processes taking place.

8. Comment on Li_2MnO_3 ^{17}O NMR Bond Pathways

The authors noted that there was a weak correlation between the hyperfine interaction strength (i.e., the bond pathway shift) and the Mn–O distance. Since the major interaction of the Mn *d* orbitals is with the 2*p* orbitals on O [Figure 2, main text], but the unpaired electron density in the *s* orbitals is the only contributor to the Fermi contact shift, it was found that short Mn–O distances and larger Mn–O–Mn angles (i.e., further from 90°) gave larger bond pathway shifts. These findings were consistent with the Goodenough-Kanamori rules: longer distances give weaker spin density transfer due to weaker orbital overlap, whilst larger angles give greater mixing of the Mn–O orbitals and a stronger polarisation of spin density. By contrast, the next-nearest and next-next-nearest pathways have a much smaller angle and distance dependence, again, as expected, due to the weaker effect of orbital overlap over more than one bond.

9. Experimental and Additional NMR Data for NCA

Experimental

Materials and Synthesis

For the pristine ^{7}Li, ^{27}Al and ^{59}Co NMR study, NCA ($Li(Ni_{0.8}Co_{0.15}Al_{0.05})O_2$), from TODA America (NAT-1050), was used as received and is henceforth referred to as TODA-NCA. The cycled and variable temperature ^{7}Li NMR was performed on the same TODA America (NAT-1050) NCA but as the material had been stored outside a glovebox for a significant period, the material was calcined at 800°C for 4hrs (5°C min^{-1} heating rate, cooled by switching off the heating elements) to remove surface species—sample henceforth referred to as HT-TODA-NCA.[44] Refinements of lab X-ray diffraction showed that HT-TODA-NCA has slightly higher Ni-Li anti-site disorder (5%) compared to the as received TODA-NCA (1%). To enable ^{17}O NMR measurements, NCA samples were enriched in the I = 5/2 NMR active nucleus ^{17}O. A previously successful strategy for performing such enrichments in Li_2MnO_3 and Li_2RuO_3 has been to heat the synthesised material under a $^{17}O_2$ enriched atmosphere.[45,46] The TODA-NCA has a primary particle size between 300 nm and 1 µm and a spherical secondary particle morphology with a radius <10 µm,[47] therefore NCA particles with a smaller particle size were desired to facilitate uniform enrichment and to maximise O-exchange. The NCA hydroxide precursors were heated at 400°C for 4 hrs (5°C min^{-1} heating rate, cooled by switched off the heating elements) to form oxides. These were annealed at 800°C for 1 hour and 700°C for 12 hours (5°C min^{-1} heating rate, cooled by switching off the heating elements). The results of this synthesis yielded a phase pure NCA powder, henceforth referred to as "primary synthesis NCA". This was subsequently enriched via secondary annealing step in a static O_2 (70% ^{17}O NUKEM Isotopes) environment at ~320 kPa at 800°C for 12 hours (5°C min^{-1} heating rate, cooled by switched off the heating elements). Both the NCA resulting from the primary synthesis and the enrichment were confirmed to be phase pure with anti-site disorder of 4.6% and 10.6% respectively, determined from Rietveld refinement using X-ray diffraction data. Attempts to perform an enrichment during the primary synthesis (i.e., annealing the NCA and Li oxides at 700°C for 12 hours and then 800°C 1 hour in the ^{17}O atmosphere) resulted in significantly high anti-site disorder >20%. The enrichments were performed with sample masses ~80 mg and with a $^{17}O_2$ volume of 12-15 cm^3.

NMR Methods

^{17}O NMR was conducted at 11.7 T using a Bruker 1.3 mm MAS probe at 60 kHz MAS frequency. Due to the extremely broad line shapes, the ^{17}O spectra were recorded as variable offset cumulative spectra (VOCS) composed of several individual rotor-synchronised Hahn echo spectra, summed together to give the full spectrum. As ^{17}O is a spin I = 5/2 nucleus, a $\pi/2$ pulse, optimised on the reference compound (0.1 M $K_3Co(CN)_6$ in D_2O) was divided by 3 to give a shorter effective $\pi/2$ pulse which will be approximately quantitative for all signals regardless of quadrupolar coupling. The radiofrequency field strength of the effective $\pi/2$ pulses (0.71 µs at 100 W) is 350 kHz which equates to ~5200ppm at 11.7 T. For an excitation of 90% for the effective π-pulse a spacing of 1.6 times this value is possible,[1] however a flat excitation profile for the full width of the spectrum is possible if the VOCs experiments are much closer together hence the spectra were spaced 2500 ppm apart from each other. The frequency range covered and number of experiments accompany the spectra. T_1 relaxation was only explicitly measured for the sharp 0 ppm component (due to the long acquisition times required for the broad component) and was ~30 ms, the broad component at high shift was assumed to be much shorter as the intensity of this broad component did not change when

the recycle delay was shortened from 100 ms to 5 ms. Due to the long spectral acquisition times, individual slices of the VOCS were recorded with a recycle delay of 5 ms except for the slices around 0 ppm in the cycled sample which used a recycle delay of 100 ms. This compromise allows the observation of both the fast and slow relaxing components of the spectra in a semi-quantitative fashion without making the acquisition times impractically long. The VOCS were collected with the aid of an automatic tuning/matching robot.[40] All ^{17}O spectra were referenced to the secondary reference CeO_2 at 877 ppm or H_2O at 0 ppm. A filtering script was applied to each sub-spectrum of the VOCS, such that the central portion of excitation is retained, whilst the remainder is set to zero; this was shown to alleviate phasing problems by Tong.[48]

^{27}Al NMR was conducted at 4.7 T using a Bruker 1.3 mm MAS probe. ^{27}Al is a spin I = 5/2 nucleus $\pi/2$ pulse, optimised on the reference compound (solid AlF_3 at −17 ppm) was divided by 3 to give a shorter effective $\pi/2$ pulse (0.75 μs at 100 W) which will be quantitative for all signals regardless of quadrupolar coupling. Note that the ^{27}Al NMR uses a solid reference rather than a liquid (as for ^{17}O and ^{59}Co); we anticipate that, whilst the effective radiofrequency (rf) field inside a liquid is smaller than a solid (due to the higher dielectric constant in the liquid state), the difference in the shorter effective $\pi/2$ pulse lengths between the solid and liquid will be minimal. Recycle delays of 0.1 s were used which is quantitative for the paramagnetically shifted Al signal (T_1s on the order of μs)[49] but not for the diamagnetic signals.

^{59}Co NMR was conducted at 4.7 T and 16.4 T using a Bruker 1.3 mm MAS probe. ^{59}Co is a spin I = 7/2 nucleus $\pi/2$ pulse, optimised on the reference compound (0.5 M $K_3[Co(CN)_6]$ in D_2O) was divided by 4 to give a shorter effective $\pi/2$ pulse (1.8 μs at 150 W) which will be quantitative for all signals regardless of quadrupolar coupling. Saturation recovery measurements for the pristine material were performed and a single component fit for the entire distribution of shifts was used and gave a T_1 = 1 ms. Recycle delays of 2 ms were used for the cycled samples to minimise acquisition times. For the pristine material recycle delays of 10 ms were used.

Fits to the ^{27}Al and ^{59}Co NMR spectra were carried out using DMFit,[50] where only the isotropic resonances were fit. Sidebands were "exploded" and allow to refine freely without restraints from a quadrupolar or CSA model; attempts to fit including the quadrupolar and/or CSA interactions yielded poor results.

Electrode Preparation and Electrochemistry

Cathodes for NMR characterisation were produced by grinding by hand, with an agate pestle and mortar, the sample, Super P carbon (Timcal) and Kynar Flex® (Polyvinylidene Fluoride PVDF) in a ratio 80:10:10 by mass for 20 minutes until well mixed. *N*-methyl-2-pyrrolidone (NMP) was added to form a slurry which was transferred onto aluminium foil and spread using a 150 μm doctor-blade. The films were dried under vacuum at 100°C overnight. Cathodes were punched to a diameter of 1.11 cm giving a surface area of 0.97 cm^2 and a mass of 6 – 8 mg per cathode (i.e., a mass loading of 6.2 – 8.2 mg cm^{-2}). Coin cells were made in an argon filled glovebox with H_2O and O_2 levels below 0.5 ppm. Cathodes were placed on steel current collectors (0.5 mm thickness) and separated from the counter electrode by glass fibre soaked with electrolyte. The anode was a lithium foil (Sigma Aldrich, 99.9 %), and backed with a steel current collector. The entire assembly was compressed by a steel wave spring and crimped. The electrolyte used was 1 M $LiPF_6$ in a 1:1 ratio of ethylene carbonate and dimethyl carbonate (EC:DMC, Sigma Aldrich).

All potentials are with respect to Li/Li^+. Currents were set at *C*-rate *C*/20 based on the active mass loading of each cell and according to the theoretical 0.8 Li capacity (i.e., corresponding to the removal of the full 0.8 Li per formula unit, or 247.6 mAh g^{-1}). Measurements were carried out on a Bio-Logic and Arbin battery cycler. Once charged, cells for NMR were deconstructed in the same glovebox, the cathodes rinsed with DMC solution (99.9 % Sigma Aldrich) and dried under vacuum for 30 minutes. The cathode material was scraped off the foil and packed into 1.3 mm zirconia rotors with Vespel caps for NMR analysis.

Pristine Material Characterisation

^{27}Al NMR

The ^{27}Al NMR spectrum for pristine TODA-NCA is shown in Figure S10(a). The spectrum is composed of two distinct regions. As in the ^{7}Li NMR spectra,[51] there is a small diamagnetic peak around 0 ppm. Typical diamagnetic chemical shifts for octahedrally coordinated Al species are 0-10 ppm with tetrahedrally coordinated Al around 50-80 ppm.[52] The peaks in this region are assigned to diamagnetic Al species, mostly likely octahedrally coordinated Al from γ-$LiAlO_2$.[53] The paramagnetic region is at a negative shift—all the Al associated within the structure is expected to produce signal in this region. DFT calculated shift contributions from a previous study[53] can be used to estimate the shift from Al environments where Al is has one or more Ni^{3+} nearest neighbours. The ^{27}Al spectrum is most intense at the most negative (most shifted) end of the paramagnetic region and it has been suggested that this indicates that the most highly shift local ordering (Al with 6 Ni^{3+} nearest neighbours) is the preferred Al coordination.[49] This is at odds with other experimental data (and the Li NMR) which do not support strong local ordering or nano-domains.[53] Another proposed assignment of the spectrum speculates that there is a preference for the Ni^{3+} JT axes to point towards the Al sites[53]—this will be discussed in more detail along with the Co NMR.

^{59}Co NMR

The ^{59}Co NMR spectrum for pristine TODA-NCA is shown in Figure S10(b). Whilst the very large shifts observed for ^{59}Co in NCA might initially appear to be due to very large positive paramagnetic Fermi-contact shifts, the greater part of the magnitude of the signal is due to chemical shift; the ^{59}Co NMR shift for $LiCoO_2$, which can be considered a diamagnetic analogue to NCA, is ~14000 ppm. If the position of the diamagnetic region is recalibrated to 14000 ppm (rather than 0 ppm which is intuitively expected), then the observed shift range of ~13000-11000 ppm is ascribed to negative Fermi-contact shifts originating from bond pathways to Ni^{3+}. The wedge-like lineshape observed for the isotropic resonances is similar to that observed for ^{27}Al, with the most intense part of the spectrum (at 11600 ppm) furthest from the diamagnetic region.

Al and Co occupy the same crystallographic sites and therefore their spectra offer two complementary probes to understand the distribution of Ni^{3+} and its JT distortion in the presence of the Co and Al "defect" sites. In the spectra for both nuclei the wedge-like lineshapes (Figure S10) suggest the most common local environments are those which give rise to large negative shifts. Al/Co lie within the *TM* layer and therefore they only have six *TM* 90° nearest neighbours (in contrast to Li which has six *TM* 90° nearest neighbours and six *TM* 180° next nearest neighbours). Each 90° Ni^{3+} bond pathway is mediated via two Ni–O–Al/Co bonds and the shift contribution of each Ni^{3+} is strongly dependent on whether one of the Ni–O–Al/Co pathways go via a JT lengthened bond (δ_{SL}) or whether both Ni–O–Al/Co are short

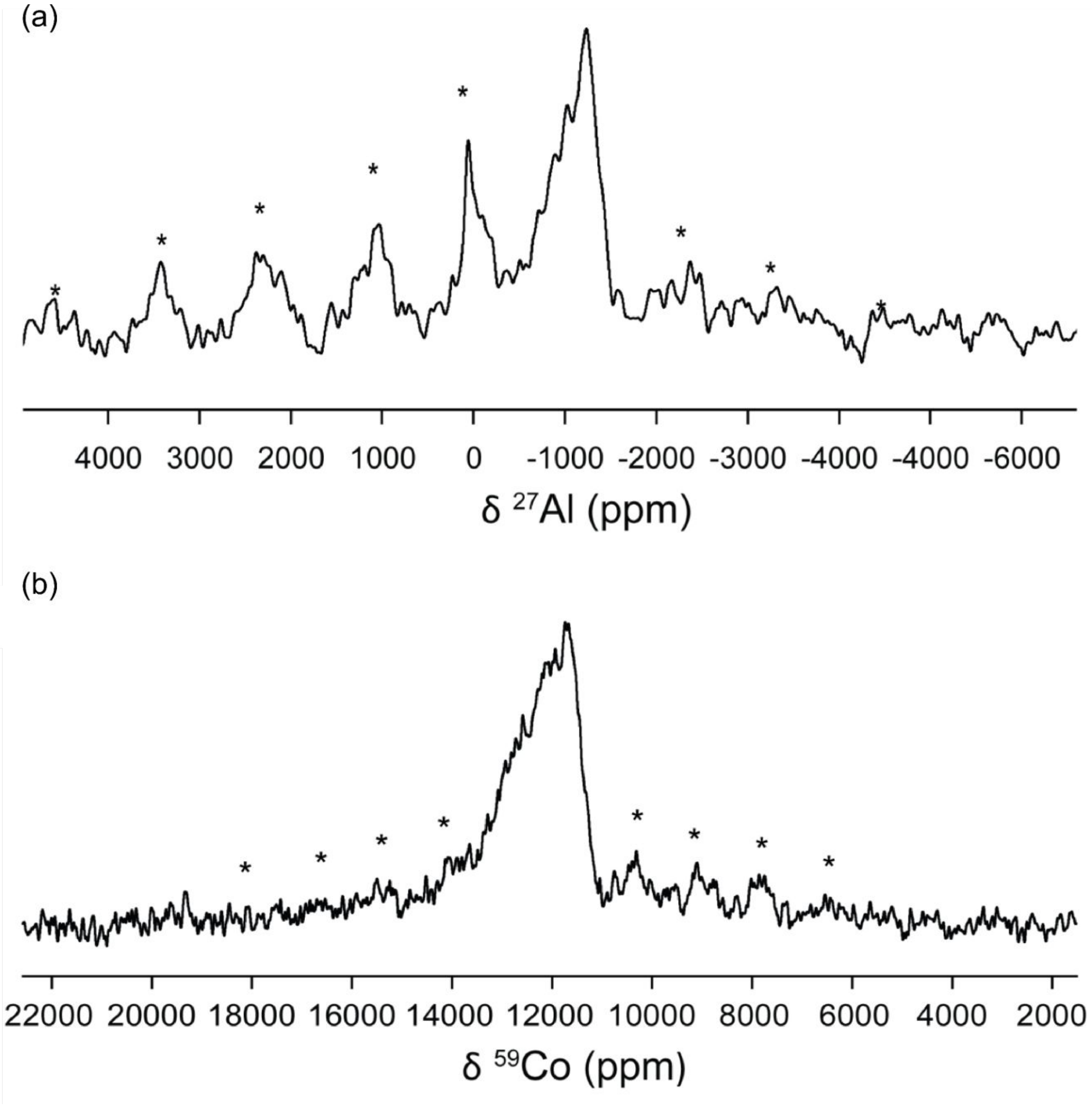

Figure S10: Pristine NCA characterisation by NMR: **(a)** the ^{27}Al NMR spectrum and **(b)** the ^{59}Co NMR spectrum; both spectra were acquired at 60 kHz MAS and 4.7 T field strength. Sidebands are indicated with asterisks.

(δ_{SS}). DFT calculations for an Al doped into a $LiNiO_2$ lattice predict the δ_{SS} bond pathway gives small positive shifts, whereas the δ_{SL} pathway gives large negative shifts (*ca.* −300 ppm).[53] As expected, similar calculations performed for Co and Al show the similar behaviour (Table S1).

For both ^{59}Co and ^{27}Al, the magnitude of the negative δ_{SL} is so much larger than δ_{SS}, that the shift of a given environment does not necessarily reflect the number of Ni neighbours, but rather the number of δ_{SL} bond pathways it experiences. The most intense parts of the spectra occur at ca. −1200 ppm and *ca.* 11600 ppm for Al and Co respectively, which are extremely close to the values predicted by the calculations for 4 δ_{SL} bond pathways (−1344 ppm and 11652 ppm respectively). These shifts could be the result of Al/Co surrounded by 4,5 or 6 Ni nearest neighbours (Figure S13).

Table S1: Comparison of DFT-calculated values for δ_{SS} and δ_{SL} bond pathways for ^{27}Al and ^{59}Co using a B3LYP functional with 20% Hartree-Fock exchange in the CRYSTAL code. [1]From ref. 48. [2]Calculated by I. Seymour.

Type of bond pathway	Calculated ^{27}Al NMR shift (ppm)[1]	Calculated ^{59}Co NMR shift (ppm)[2]
δ_{SS}	11	16
δ_{SL}	−336	−587
Dynamic average, $(2\delta_{SS} + \delta_{SL})/3$	−220	−386

With the caveat that a static, cooperative JT distortion does not occur for NCA, and that on the "NMR timescale" (10s of μs) the JT axes are in flux, it is worth considering which local coordination environments are likely to be thermodynamically favourable and therefore make up a larger part of the average structure sampled in the NMR spectra. By considering how defect atoms can be accommodated in an ordered array of JT bonds, a picture of these favourable local environments can be built up.

One striking absence from both the ^{27}Al and ^{59}Co spectra is the lack of peaks at even lower shifts, resulting from Al/Co with five or six δ_{SL} bond pathways. Such environments should be possible for five or six Ni neighbour environments. Doping a single defect into either a layered $LiMnO_2$-like structure[54,55] or into a zig-zag ordered structure,[53] results in a defect environment with $4\delta_{SL}$ bond pathways (Figure S13(a)). This configuration causes the minimum disruption to the Ni^{3+} JT array. Reorientation of JT long axes such that there are $5\delta_{SL}$ pathways (Figure S13(b)) disrupts the pairwise coupling of JT axes between adjacent Ni^{3+} ions. Such pairwise couplings are predicted by first principle calculations to be stabilising.[56] Similarly for a single defect reducing the number of δ_{SL} bond pathways below four requires distortion of the Ni^{3+} pairing and the creation of O sites which must be highly distorted. This argument suggests that for a defect atom surrounded by six Ni neighbours, the most energetically favourable configuration will have $4\delta_{SL}$ (and $24\delta_{SS}$) pathways.

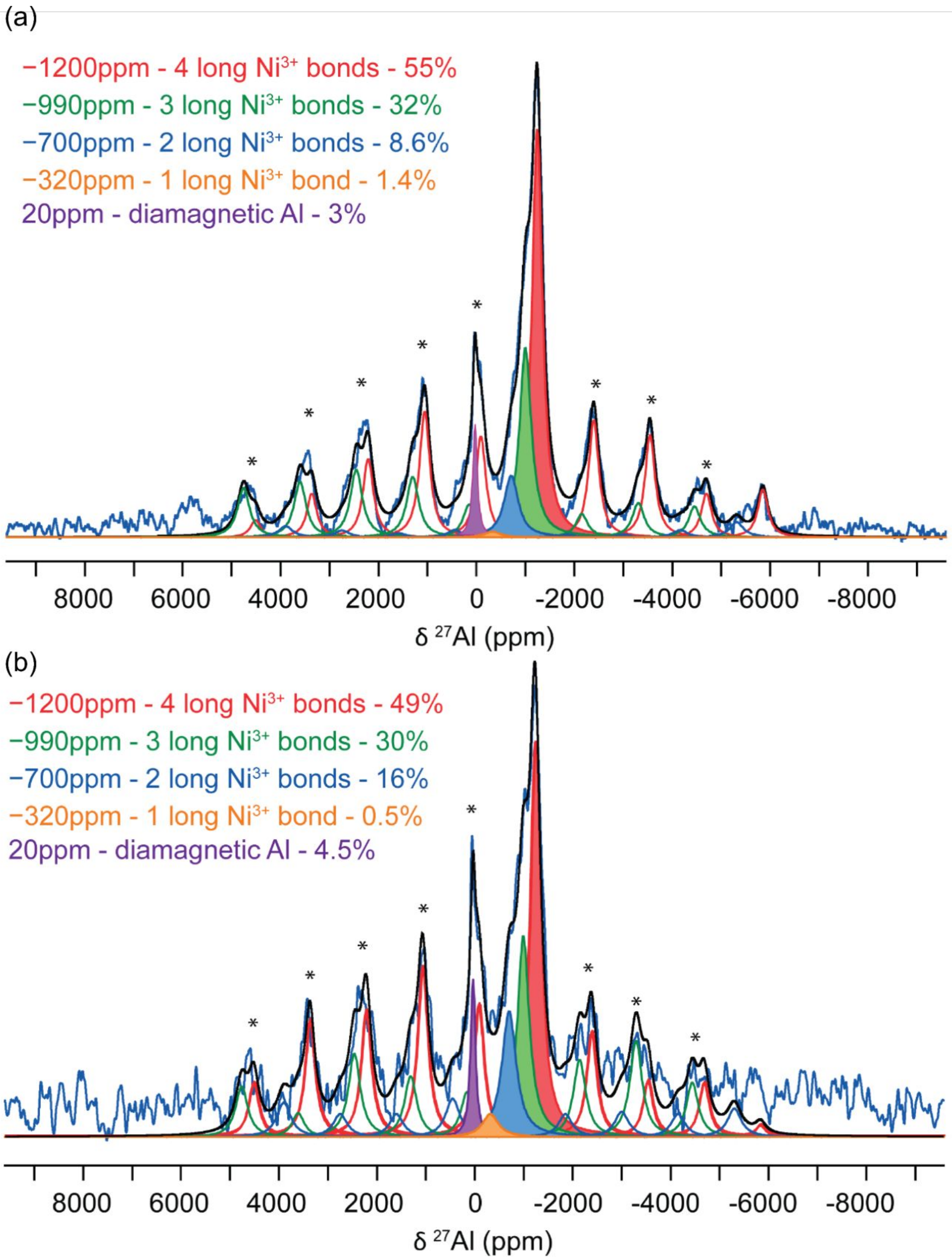

Figure S11: ^{27}Al NMR spectrum of TODA NCA at 4.7 T and 60 kHz MAS frequency. **(a)** shows the spectrum recorded using a π/2 pulse optimised on AlF_3. In the π/2 experiment, the peak at the highest shift, assigned to environments with $4\delta_{SL}$ bond pathways, is enhanced. **(b)** shows the spectra recorded a shorter π/6 pulse. This leads to quantitative spectrum although the signal-to-noise ratio is reduced. The fitted isotropic resonances are shown in filled colour whilst their corresponding sidebands are shown by the outlined peaks. Note that the high baseline in **(b)** arises from a fit to the baseline at higher and lower frequencies.

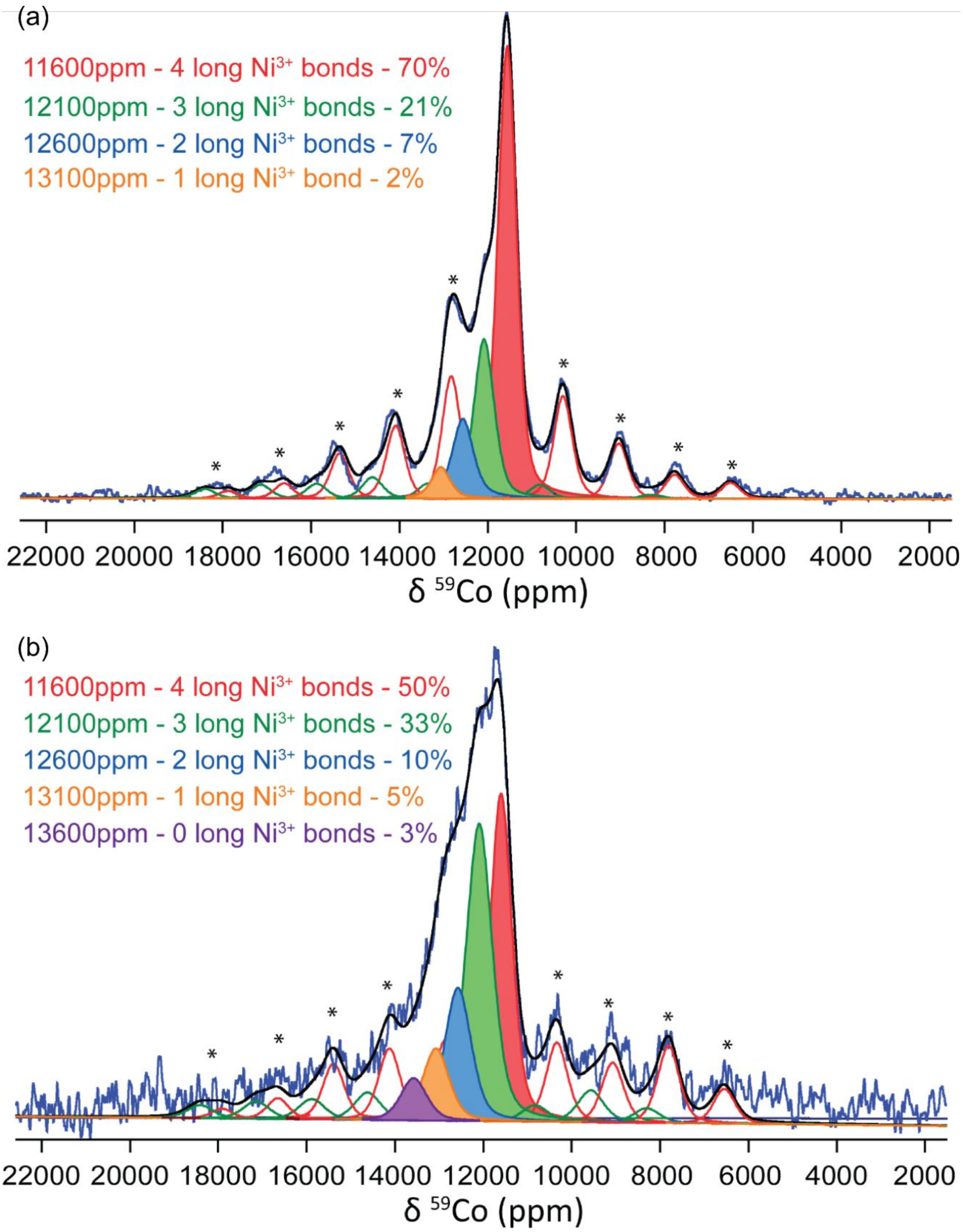

Figure S12: ^{59}Co NMR spectra for TODA-NCA at 4.7 T and 60 kHz MAS frequency. **(a)** shows the spectrum recorded using a $\pi/2$ pulse optimised on 0.1 M $K_3[Co(CN)_6]$ in D_2O. The use of a longer pulse leads to an enhancement of the signals from environments with lower quadrupolar coupling constants (C_Q). In the $\pi/2$ experiment, the peak at lowest shift, assigned to environments with $4\delta_{SL}$ bond pathways, is enhanced. **(b)** shows the spectra recorded a short $\pi/8$ pulse. This leads to quantitative spectrum although the signal-to-noise ratio is reduced. The fitted isotropic resonances are shown in filled colour whilst their corresponding sidebands are shown by the outlined peaks.

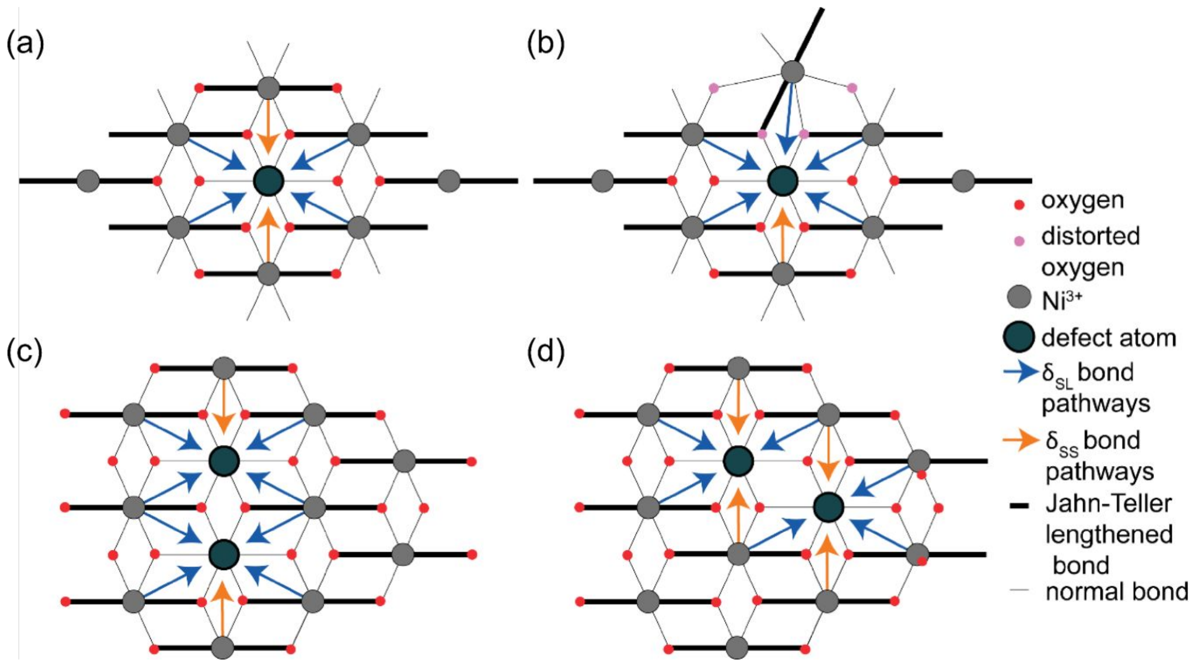

Figure S13: Schematic representation of introduction of defect atoms into a cooperatively distorted JT lattice (layered $LiMnO_2$-like). **(a)** shows the $4\delta_{SL}$ + $2\delta_{SS}$ bond pathway environment that would be expected for the introduction of one defect atom. **(b)** Illustrates the distortion that must occur for a $5\delta_{SL}$ + $1\delta_{SS}$ bond pathway environment to occur. Disrupted oxygen ions are shown in pink. **(c)** and **(d)** show the possible organisation of two defect sites (i.e., an Al or Co sites with only 5Ni ions). **(c)** demonstrates the $4\delta_{SL}$ + $1\delta_{SS}$ coordination and d) the $3\delta_{SL}$ + $2\delta_{SS}$ coordination showing how both may be coordinated without large disruption of the Ni-Ni JT interactions. Ni atoms are show in grey, defect atoms in blue, oxygen in red, highly disrupted oxygen in green, bonds are shown by black lines with thick black lines representing the long JT axes for each Ni, δ_{SL} bond pathways are shown by blue arrows and δ_{SS} bond pathways by orange arrows.

The case of defect atoms surrounded by four or five Ni^{3+} is slightly more complex. Two adjacent defect atoms (creating two 5Ni neighbour sites) can be arranged, without disrupting the Ni^{3+} array, to create two local environments one with $4\delta_{SL}$ and one with $3\delta_{SL}$ (Figure S13(c) and (d)). Similarly, there are five arrangements of three defect atoms which can give a $4Ni^{3+}$ neighbour environment with $4\delta_{SL}$, $3\delta_{SL}(\times2)$ or $2\delta_{SL}(\times2)$. Without knowing the energetic preference, or additionally in the case of three or more defects, the preferred clustering geometry, it is not possible to say which are the lowest energy environments. However, it is clear there isn't a strong preference for defect atoms surrounded by 4/5 Ni^{3+} to have $4\delta_{SL}$ pathways, as this would result in 90% of the signal occurring at −1200 ppm and 11600ppm for Al and Co respectively, which is not observed experimentally (Table S3). If it is assumed that all the arrangements that cause minimal distortion of the JT axes are equally probable, then the random solution probabilities of each number of Ni neighbours can be converted into an expected distribution of $4\delta_{SL}$, $3\delta_{SL}$ and $\leq2\delta_{SL}$ environments (Table S2). There is excellent agreement between this model and the integrated intensities of both the ^{59}Co and ^{27}Al NMR spectra (Table S3).

Whilst this model cannot separate the specific local environments and clusters which make up the thermodynamic minima in the JT orientation energy surface, it does demonstrate that the energy surface is far from flat. On average the JT distortion adopts a kind of pseudo-

ordered state where local defects are accommodated by coordination environments which resemble those expected for a totally ordered JT array, these low energy states can easily interconvert and do so on the timescale much faster than that of the NMR experiments.

The integrated intensities of the NMR spectra change, particularly in the case of ^{59}Co, depending on the pulse length used. The use of the $\pi/2$ pulse, optimised on a non-quadrupolar reference, exploits the differences in nutation behaviour for environments with different quadrupolar coupling constants (C_Q), enhancing those signals with smaller electric field gradient anisotropy (Figure S11(a) and Figure S12(a)). In this case, the highly shifted peaks at −1200 ppm and 11600 ppm, for Al and Co respectively, increase in intensity. This supports the assignment that these peaks are at least partly caused by the more symmetric Al/Co-6Ni type environments. The larger enhancement for ^{59}Co stems from its larger nuclear quadrupole moment (Q) compared to ^{27}Al (0.42 barns *vs* 0.15 barns).

Table S2: Probability distribution for Ni coordination of "defect atoms" in NCA and the combination of these probabilities with the fraction of configurations which give rise to different bond pathways as described in the text, giving a prediction of the fractions for the fitted NMR spectra.

No of Ni neighbours	Random solution probability	Possible δ_{SS} and δ_{SL} configurations	Expected fraction of environments	Bond pathway configuration	Predicted fraction of NMR signal
6	0.2621	$2\delta_{SS} + 4\delta_{SL}$	1	$4\delta_{SL}$	0.51
5	0.3932	$1\delta_{SS} + 4\delta_{SL}$	½	$3\delta_{SL}$	0.29
		$2\delta_{SS} + 3\delta_{SL}$	½	$\leq 2\delta_{SL}$	0.20
4	0.2458	$0\delta_{SS} + 4\delta_{SL}$	1/5		
		$1\delta_{SS} + 3\delta_{SL}$	2/5		
		$2\delta_{SS} + 2\delta_{SL}$	2/5		

Table S3: Results of fitting the ^{27}Al and ^{59}Co NMR spectra of pristine TODA-NCA.

Peak Assignment	^{27}Al			^{59}Co		
	Shift (ppm)	Relative Integration (%) Quadrupolar ($\pi/6$)	Relative Integration (%) Non-Quadrupolar ($\pi/2$)	Shift (ppm)	Relative Integration (%) Quadrupolar ($\pi/6$)	Relative Integration (%) Non-Quadrupolar ($\pi/2$)
$4\delta_{SL}$	−1200	49	55	11600	50	70
$3\delta_{SL}$	−990	30	32	12100	33	21
$2\delta_{SL}$	−700	16	8.6	12600	10	7
$1\delta_{SL}$	−320	0.5	1.4	13100	5	2
$0\delta_{SL}$	0	4.5	3.0	13600	3	0

Cycled NCA Characterisation

NMR spectra of TODA-NCA upon delithiation are shown in Figure S14 and Figure S15 for ^{27}Al and ^{59}Co, respectively. For all nuclei measured in the pristine material, the distribution of shifts was assigned by considering first the number of Ni^{3+} neighbours and, for ^{59}Co and ^{27}Al, combining this with likely JT orderings to deconvolute the observed NMR lineshape. In all cases, the presence of Ni^{3+} neighbours determine centre of mass of the shift. As Ni^{3+} is oxidised a similar shift trend is expected for the ^{59}Co, ^{27}Al and ^{17}O as was observed for ^{7}Li[51]—i.e., a shift toward the diamagnetic region as delithiation proceeds.

^{27}Al NMR

The ^{27}Al NMR spectra show the expected trend of a gain in signal in the diamagnetic region at the expense of the paramagnetic sites. The spectra we recorded with short recycle delays (0.1 s) in order to maximise any paramagnetic signals and are not quantitative in the diamagnetic region. Despite this biasing of the spectra no paramagnetic Al sites are observed at high states of charge in contrast to the ^{17}O VOCS. It has been proposed that at the end of charge Al migrate into tetrahedral sites in the Li layer in Al containing $Li\mathit{TM}O_2$, particularly in the case where Al is coordinated by 6Co.[57] Whilst such environments are statistically unlikely to occur in NCA there may still be migration of Al into tetrahedral sites. The ^{27}Al shift for tetrahedral Al in a diamagnetic site is ~70 ppm.[58] No clear evidence for a peak at this shift was observed in the majority of samples, although there is definitely signal above 0 ppm particularly for the 0.16 sample, which, given possibility of 2nd order quadrupole shifts at low fields and the low signal to noise ratios, is not inconsistent with tetrahedral sites. For an NCA sample charged to 4.8V and then potentiostatically charged until the expended capacity was 270 mAh g^{-1}, the peak moved to higher ppm, with a shoulder at ~73 ppm. This change was only visible when a non-quadrupolar $\pi/2$ pulse was used (Figure S14 bottom). This observation supports the hypothesis that some Al migrates into the *TM* layer at the end of charge. The peak is only clearly observed in the sample which is potentiostatically charged suggesting this migration occurs at the highest levels of delithiation. Additionally, most of the signal remains in octahedral environments—notably the “tetrahedral” signal can only be observed clearly using $\pi/2$ pulse experiments implying a lower electric field gradient anisotropy for this environment. A more detailed study would be required to quantify the relative amounts of each of the Al environments at the end of charge.

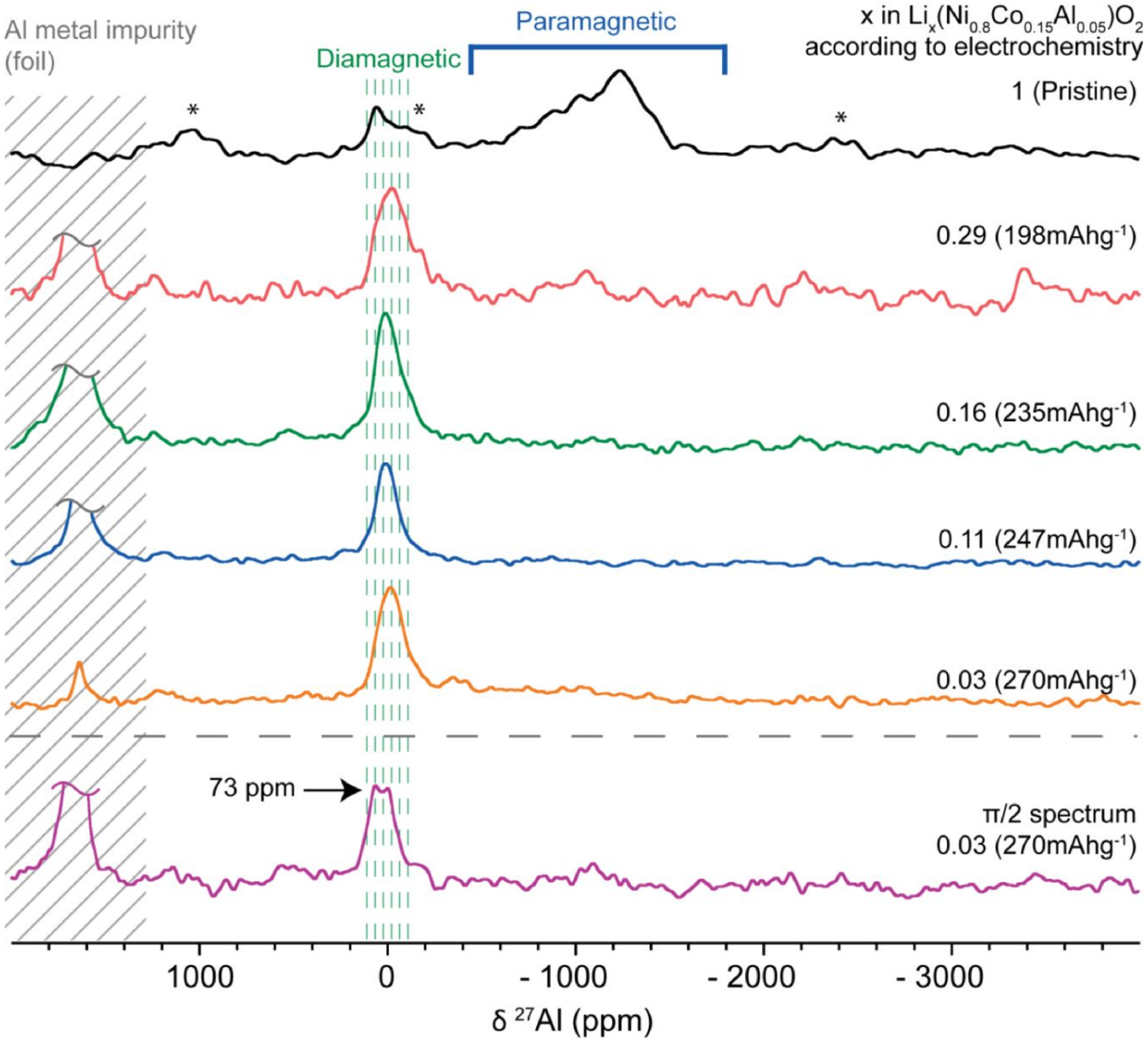

Figure S14: ^{27}Al NMR spectra for TODA-NCA recorded at 4.70 T and 60 kHz MAS frequency various states of charge. Shift regions for paramagnetic (blue), diamagnetic (green) and impurity metal (grey) are indicated. All spectra except the bottom spectrum are recorded with $\pi/6$ pulses used as the effective $\pi/2$ pulse to quantitatively excite all signals. The bottom spectrum was recorded with a $\pi/2$ pulse as optimised for solid AlF_3.

^{59}Co NMR

Most of the ^{59}Co spectra of cycled NCA, were recorded at higher fields than the pristine spectra discussed previously. The spectra of cycled ^{59}Co samples, which contain less observable Co^{3+} near the end of charge, could be acquired much more quickly at 16.4 T. Additionally, the electric field gradients at the nucleus are expected to become larger as the charge state of the adjacent ions is changed and Li is removed, hence a higher field is desirable. This has the unfortunate effect of increasing the broadening due to the e-n dipolar coupling. As the observable ^{59}Co signal decreases in intensity, wide sweeps using variable offsets were performed to ascertain whether the signal became significantly broader, or whether new signals could have emerged; between −1000 ppm and 30000 ppm no new signals were observed.

The ^{59}Co spectra are scaled by mass and scans and their integrated intensities (in relation to the pristine material) are also listed (Figure S15). There is a loss of signal with delithiation although even at the end of charge some ^{59}Co signal remains (35%). In the pristine material the Co^{3+} is diamagnetic and hence observable by NMR. The ^{59}Co NMR signal is not expected to be observable for Co^{4+}; Since Co^{4+} is itself paramagnetic, the effective spin transfer will be extremely large, hence extremely rapid relaxation will be induced, causing a loss of signal. It is likely that the signal loss in the ^{59}Co spectrum on delithiation is as a result of $Co^{3+} \rightarrow Co^{4+}$ oxidation. It is possible that the loss of signal is due to faster T_2 relaxation during the echo delay for the more delithiated samples; the spectra are recorded as Hahn-echoes to reduce probe ringing and background. Whilst, in the cycled ^{7}Li NMR the T_2 relaxation was enhanced on delithiation,[51] this was an effect of the increased Li motion. In the case of Co/Al/O the T_2s would be expected to increase as the sample becomes less paramagnetic overall due to Ni^{3+} oxidation. This possibility warrants further investigation.

The change in peak position for the ^{59}Co spectra is more challenging to rationalise. At the nominal composition $x = 0.91$, the peak shifts towards the diamagnetic region (14000 ppm) as expected. However, once the composition reaches $x = 0.56$, the peak returns to approximately the shift of the pristine material (*ca*. 12000 ppm). Between compositions $x = 0.56$ and $x = 0.29$ the Co NMR barely changes with only a small loss in signal and virtually no peak shift observed. Finally, upon delithiation to the end of charge the remaining Co signal is shifted further from the expected diamagnetic region with a peak position ~11500 ppm. The nominal composition in this case is −0.08, i.e., the nominal electrochemical capacity corresponds to the removal of more lithium than implied by the stoichiometry of the sample, assuming 100% coulombic efficiency. The cells for ^{59}Co NMR were cycled at *C*/50 in order to ensure low overpotentials, hence some of the erroneous capacity observed for the highly delithiated sample can be attributed to electrolyte decomposition, and its true Li stoichiometry can be assumed to be at or close to the maximum delithiation assuming Al^{3+} is electrochemically inactive of $x = 0.05$.

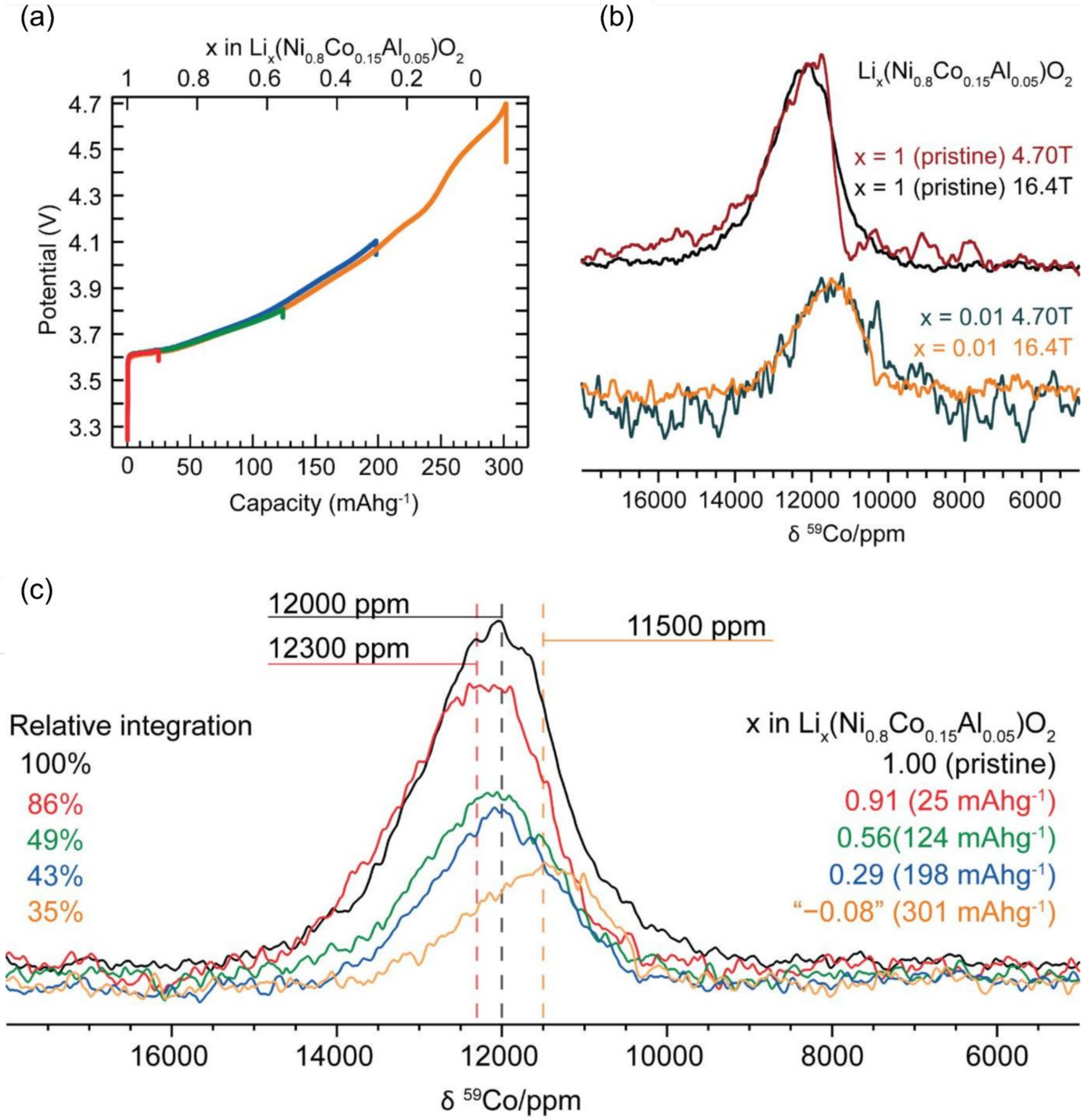

Figure S15: (a) Voltage curve showing states of charge sampled for the ^{59}Co NMR study. **(b)** ^{59}Co spectra for pristine and cycled TODA-NCA at 4.70 and 16.4 T demonstrating the lack of a significant 2nd order quadrupolar shift for either sample. **(c)** ^{59}Co spectrum for TODA-NCA at various points of charge with labels indicating the relative integration and the Li remaining per formula unit (derived from electrochemistry) in each case.

This unanticipated shift in the ^{59}Co resonances is intriguing, particularly as similar behaviour is not observed in the ^{27}Al, ^{17}O or ^{7}Li.[51] One possible factor could be a large shift caused by the 2nd order quadrupolar interaction, however this can be ruled out as the no change in peak position is observed for the pristine sample or a sample at the end of charge at different field strengths (Figure S15(b)). A second possibility is that, for the portion of Co^{3+} that remains, whilst the hyperfine shift from Ni^{3+} is lost a new shift from paramagnetic Co^{4+} arises. This is also not likely, as the shifts from Co^{4+}–O–Co^{3+} arise from unpaired spins in the t_{2g} orbitals, the dominant shift interaction (which would occur via a delocalisation mechanism) would be the 90° interaction and it would be expected to be positive, contrary to the observed shift. The

180° Co^{4+}–O–Co^{3+} shift is expected to be negative but cannot explain the shift as, without significant Co^{4+} migration to the Li-layer, Co^{3+} can have no Co^{4+} 180° neighbours.

The peak which remains at the end of charge is on the highly shifted end of the pristine Co spectrum and in the pristine material fits the shift assigned for Co with 4δ_{SL} Ni^{3+} pathways (11600 ppm). This raises the possibility that at the end of charge, the remaining Co^{3+} is still coordinated by Ni^{3+} and specially via δ_{SL} pathways. If in the pristine material a Co^{3+} surrounded by 4δ_{SL} pathways, for example Co-6Ni, were particularly energetically favourable, possibly due to favourable JT ordering accommodated by the Co^{3+}, then such a region could be more difficult to oxidise and could lead to a region that remains largely 3+. This is also seems unlikely, as if the Co NMR is quantitative and 35% of the Co is in these domains, 18-27% of the Ni in the sample would be required to remain 3+ (depending on whether the stable domain requires 4, 5 or 6Ni^{3+} ions to form). Additionally, this hypothesis is not supported by the ^{17}O NMR where the O coordination expected for this type of domain is absent (i.e., O with one long JT bond to Ni^{3+} and two other bonds); 9-14% of the O should be in this environment. Although it is true that, if such, essentially static, JT domains existed then the ^{17}O signal may be shifted or unobservable due to the large EFG anisotropy that a static JT arrangement would create. The lack of evidence for Ni^{3+} in either the ^{27}Al or ^{7}Li NMR spectra[51] is also convincing evidence against this hypothesis—it is hard to imagine 3+ domains making up significant proportion of the *TM* layer not interacting with either ^{7}Li or ^{27}Al.

One very interesting property of ^{59}Co NMR is the extremely large chemical shift range observed for diamagnetic ^{59}Co complexes. The source of these large chemical shifts is the so-called paramagnetic contribution to the diamagnetic chemical shift. This shift is derived from the mixing of symmetry-allowed, low lying excited states with the ground state and should not be confused with the paramagnetic Fermi-contact shift.[59] The size of this chemical shift will increase if the energy difference between the ground state and the lowest lying excited states decreases. As the ground state of Co^{3+} is low spin d^6 $t_{2g}^6e_g^0$ and the low-lying excited states have the configuration $t_{2g}^6e_g^1$, for Co^{3+} energy difference is simply the crystal field splitting energy (Δ). In Co^{3+} complexes the paramagnetic chemical shift is lowest for strong field ligands, such as $(CN)^-$, which increase Δ,[60] hence $K_3Co(CN)_6$, which is the primary reference for ^{59}Co resonates at much lower shift (0 ppm) than does $LiCoO_2$ ~14000 ppm) (Figure S16).

In NCA or $LiCoO_2$ the energy separation, Δ, is between the non-bonding Co t_{2g} states and the Co–O antibonding e_g orbitals. For $LiCoO_2$, the Co *K*-edge spectrum does not show the same continuous edge shift observed for Ni and Mn complexes.[61–63] However, the $L_{II,III}$-edge XANES, which are sensitive to transitions from 2*p*→3*d* do show a shift to higher energy.[64] As no edge shift is seen in the *K*-edge spectra, the changes in the $L_{II,III}$-edge spectra are most likely not simply due to an increase in the oxidation state of the Co ion. Rather, the energy gap between the 2*p* and 3*d* states has increased as the vacant 3*d* states (e_g orbitals) are pushed up in energy. This rise in the energy of the e_g states is caused by a lowering in energy of the Co–O e_g bonding states due to increasing hybridisation. *L*-edge XANES show a similar trend.[65] Increasing hybridisation of the Co^{3+}–O states should, as observed in the *L*-edge- XANES, result in an increase in Δ and hence a reduction in the paramagnetic chemical shift. The change in peak position for the ^{59}Co spectrum is therefore dependent on two principal factors: loss of the Ni^{3+} negative Fermi-contact shifts (principally via δ_{SL} bond pathways) and the reduction in positive, chemical shift caused by an increasing energy separation of the t_{2g} and e_g levels in Co due to increased Co–O hybridisation. The ^{59}Co NMR must be understood in the context of complementary techniques such as L-edge XANES, EXAFS and XRD, all of which offer information about the Co–O bond lengths and hybridisation.

During the initial period of discharge, from $x = 1$ to $x = 0.91$, signal is lost, which suggests Co is oxidised from the beginning. The peak position moves slightly towards higher shifts, which indicates that initially the loss of Fermi-contact shift outweighs the loss of chemical shift. In the next part of the charge $x = 0.91$ to $x = 0.6$ more Co and Ni are oxidised, and more Co signal is lost. In this part of the charge the loss of Fermi contact shift is dominated by the effects of the changing chemical shift of the residual Co^{3+} ions that remain in the structure. The period of charge from $x = 0.60$ to $x = 0.36$ sees very little change in the intensity of the Co spectrum perhaps indicating that the change in the Co oxidation state has ceased temporarily. At the same time the shift does not change either indicating a balance between the changes due to the Fermi-contact and chemical shifts. From the $x = 0.6$ to $x = 0.91$ more Co signal is lost and the change in the peak position indicates the change of the chemical shift outweighs the change in the Fermi-contact shift contribution.

This non-monotonic behaviour is somewhat challenging to interpret however there is no reason to assume the evolution of Fermi-contact and chemical shift contributions are monotonic either. Whilst, the majority of *L*-edge XANES edge shift occurs before $x = 0.24$, EXAFS data show that the Co–O bond length changes continually with large change between $x = 1$ and $x = 0.42$, a period between 0.42 and 0.17 where the change is fairly small and finally a period between 0.17 and 0.06 where the change is faster again.[65] This is in reasonable agreement with the observed change in the Co shift (Figure S16). Another possible origin of discrepancy between the XANES data and the ^{59}Co NMR is that in the ^{59}Co NMR, signal is only observed for Co^{3+} whereas XANES measures the signals regardless of its formal oxidation states. The formation of Co^{4+} is likely to drive a shortening of the Co–O bond lengths due to its higher charge. By contrast, Co^{3+} is more likely to have its bond length and hybridisation changes forced upon it by the changes in the lattice, as a result of the loss of Li and the changes in oxidation of other ions. This may create a lag between the changes observed in the XANES and the NMR. As discussed earlier, the majority of the Fermi-contact shift on Co comes via δ_{SL} bond pathways which depend on the orientation of the Ni^{3+} long JT axes. The JT distortions in NCA are dynamic, however as discussed previously, the more thermodynamically favourable arrangements will make up more of the average ordering which is probed on the NMR timescale. The loss of the Fermi-contact shift may not be monotonic, for example the balance of Ni^{3+}/Ni^{4+} may reach a critical point during charge, at which the pseudo-ordering of the JT axes is be lost and the average shift experienced by Co^{3+} changes dramatically. A sudden change in Fermi-contact shift, accompanied by a relatively small change in the chemical shift, is one possible explanation for the relatively static Co peak position between $x = 0.6$ and $x = 0.36$. The sharp change at the end of charge is accompanied by reduction in the Co–O bond length (as observed in EXAFS) however there is also the collapse of the c-lattice which leads to an even more compressed *TM*–O layer. This may introduce strains around Co^{3+} which remains in the lattice (35% of the NMR signal) which compress its octahedra and help drive the sharp change in peak position from $x = 0.36$ to $x = 0.01$. More measurements of both the ^{59}Co NMR and the Co XANES are required if more mechanistic detail is to be uncovered.

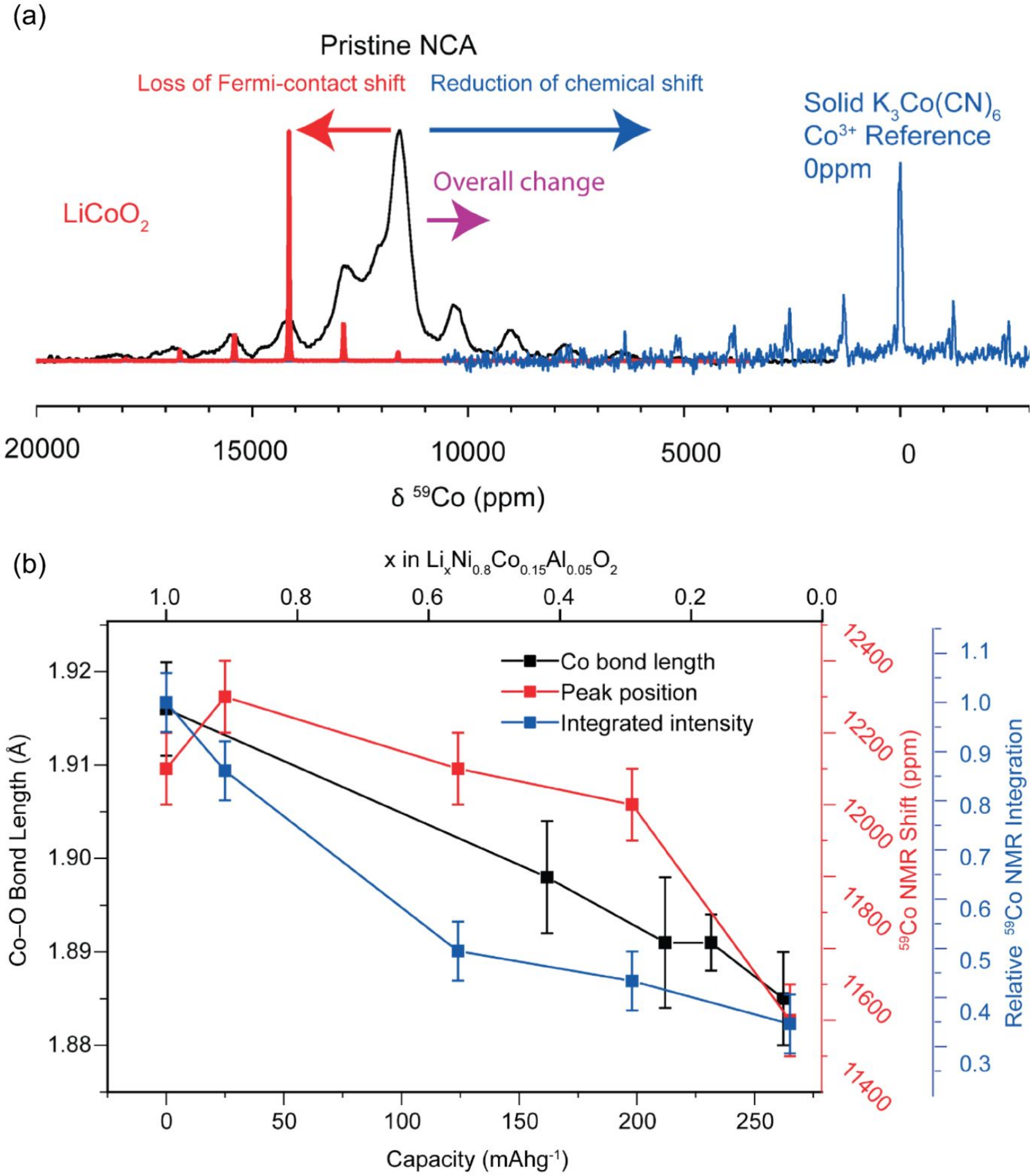

Figure S16: (a) Illustration of the shift ranges for $LiCoO_2$ (red), pristine NCA (black) and solid $K_3Co(CN)_6$ (blue). The loss of NCA's Fermi-contact shift is expected to move it towards the $LiCoO_2$ shift region, whilst the reduction in the chemical shift due to increasing Δ is expected to move it towards the $K_3Co(CN)_6$ region. The overall change is a slight shift to lower ppm (purple). **(b)** comparison of the evolution of Co–O bond length, ^{59}Co NMR shift and ^{59}Co integrated intensity versus the SOC of TODA-NCA. Co–O bond lengths derived from EXAFS and taken from ref. 59.

10. Comment on EPR of $Li_{1.2}Ti_{0.4}Mn_{0.4}O_2$

To assist their assertion of the formation of $(O_2)^{n-}$ species in $Li_{1.2}Ti_{0.4}Mn_{0.4}O_2$, Geng *et al.* carried out EPR measurements.[66] Briefly, EPR provides complementary information about the local environment of unpaired electron spins in a material; by examining and fitting the spectra to obtain the *g*-values of a material, the local changes in electronic structure may be readily monitored. The authors saw, at the end of charge, a new resonance centred at g = 2.010(6), which they assigned to $(O_2)^{n-}$, but without additional calculations or experiments. This assignment, however, is less plausible based on the *g*-shifts observed and those reported in other superoxide species;[14,67] furthermore the ~~and~~ the resonance appears to be isotropic, rather than axial. The *g*-value and *g*-anisotropy is therefore noticeably different to that of a superoxide. The Mn^{4+} (S = 3/2) ions in this material could also give rise to an EPR signal. Of note, an isolated superoxide $(O_2)^{n-}$ ion (where n = 1) gives rise to an EPR signal, while the NMR signal, if observable, will have an extremely large hyperfine interaction. An isolated peroxo species (n = 2) has *no* EPR signal.

11. Curie-Weiss Shift Conversions for Li-rich NMC ^{17}O NMR

As stated in the main text, the Fermi contact shift, δ_{FC}, depends on the bulk magnetic susceptibility of the sample (eq. 2). Consequently, δ_{FC} may be re-written to include the Curie-Weiss law:

$$\delta_{\mathrm{FC}} = \frac{\alpha}{(T-\theta)}, \qquad \text{(S-5)}$$

where T is the experimental temperature, θ is the Weiss constant and α is a constant equal to $\frac{2SA_{\mathrm{iso}}C}{N_{\mathrm{A}}\mu_0\mu_{\mathrm{B}}\mu_{\mathrm{N}}g_{\mathrm{e}}g_{\mathrm{N}}}$ (and C is the Curie constant).

In the work by House *et al.*, a sharp feature at approximately 3000 ppm is seen.[20] This experiment was carried out at a temperature of approximately 310 K (due to frictional heating from spinning at 34 kHz) field of 9.45 T, meaning that the 0 ppm ^{17}O Larmor frequency is 54.3 MHz and therefore the observed resonance occurs at 54.4629 MHz.

According to the paper referenced by House *et al.*, at 77K, $^{17}O_2$ resonates at a field of 8220 G (corresponding to 0.822 T).[68] Therefore, the Larmor frequency of $^{17}O_2$ at 77 K is 4.74425 MHz. As the static field applied was 0.8 T (a Larmor frequency of 4.61728 MHz), the shift of $^{17}O_2$ under these conditions is therefore 27500 ppm. Using the Weiss constant supplied by the reference paper, θ = −71.3 K, we obtain the shift of $^{17}O_2$ at 310 K as 10700 ppm, instead of 3000 ppm.

12. Results obtained from *ex situ* and *in situ* ^{17}O NMR of Li_2MnO_3

In the work by Li *et al*. on Li_2MnO_3, both *ex situ* and *in situ* ^{17}O NMR was carried out.[69] In the *ex situ* pjMATPASS experiments, as in pristine Li_2MnO_3, five unique resonances were identified; the assignment of these was given earlier. Initially on charging, Li *et al.* observed slight a decrease in the intensity of all resonances, with the resonances corresponding to O in longer Mn–O bonds decreased more O bound to Mn *via* a short bond, consistent with the oxidation of small amounts of residual (JT-distorted) Mn^{3+} to (undistorted) Mn^{4+}. Further charging produced a small resonance near 1700 ppm [Figure S17(a)] which was not assigned but may correspond to degradation products, formed either from proton insertion and/or densification *via* O_2 loss;[70–74] this resonance also appears in the spectrum obtained by Li *et al.* after one full cycle. On discharging, the intensities increase but do not return to the same intensity observed for pristine Li_2MnO_3 [Figure S17(b)]. In particular, the intensity of ^{17}O resonances in stacking faulted domains decreased more than those in 'ideal' (*C*2/*m*) domains. Li *et al*. attribute this to greater delocalisation of electron density across Mn and O, *via* π redox, a mechanism in which hybridised Mn–O π-like orbitals participate in redox and stabilise the charged state (see SI and ref. [24] for a more detailed description). They suggest that these stacking faults promote π redox, as the faulted domains are more capable of accepting the structural distortions induced by π redox than the *C*2/*m* domains.

The *in situ* ^{17}O NMR spectra bear out the *ex situ* observations: a decrease in the ^{17}O intensity on charging—ascribed to the formation of paramagnetic O species which the authors indicate would not be visible in NMR due to rapid nuclear relaxation times—and an increase on discharge as O becomes less paramagnetic which the authors suggest is due to the reduction of Mn–O π units [Figure S17(c), (d) and (e)]. A loss of intensity may be ascribed to the formation of paramagnetic species, but, as Li *et al.* point out, determining exactly what the nature of these species is (Mn–O π-like units, trapped O_2, holes on O) from ^{17}O NMR is not possible without the assistance of *ab initio* calculations and additional supporting data.

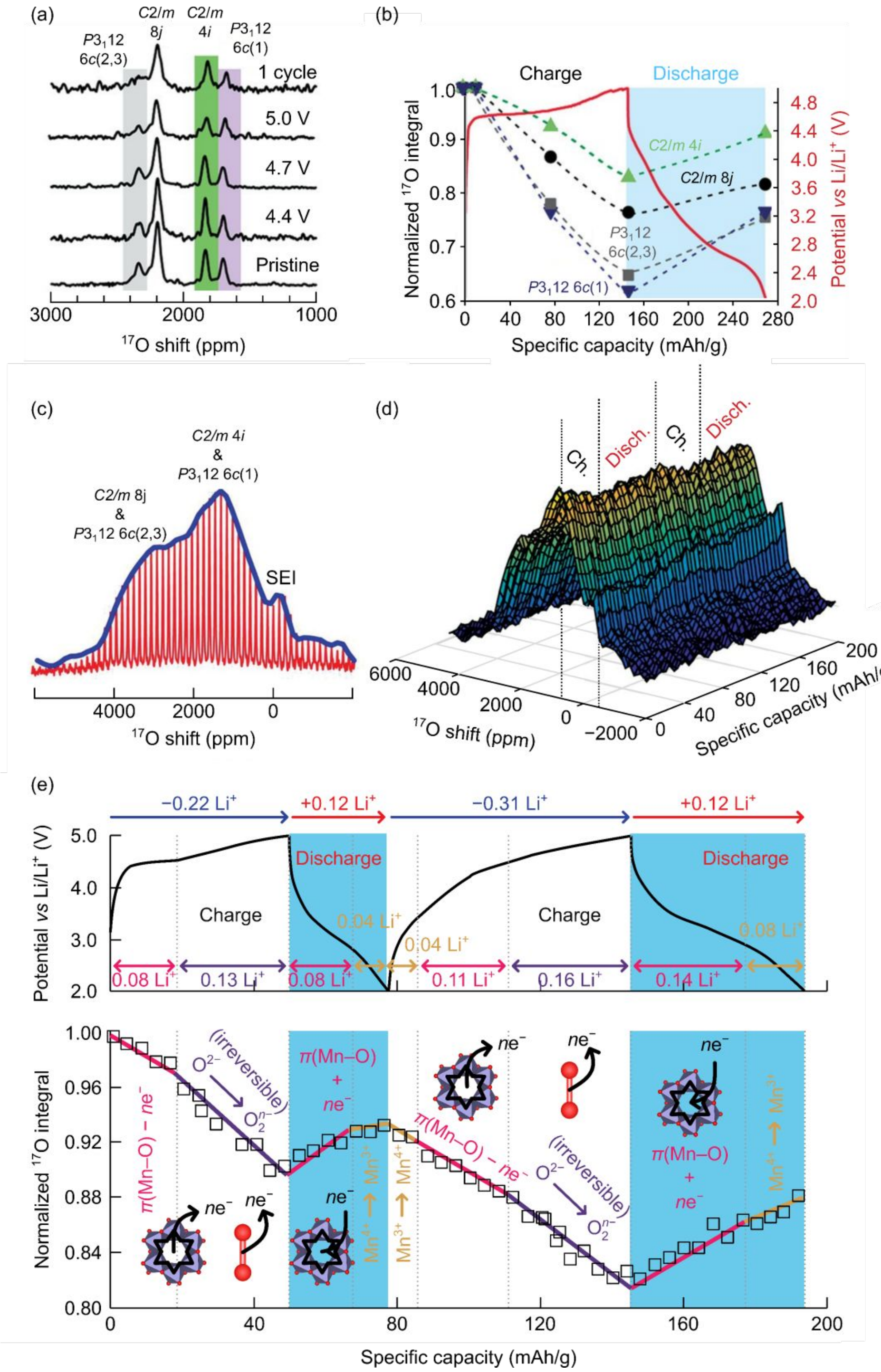

Figure S17: ^{17}O NMR of Li_2MnO_3 when charged and discharged. In **(a)** the ex situ pjMATPASS spectra recorded at 19.6 T under MAS rate 18 kHz; **(b)** shows the voltage profile for an Li metal/Li_2MnO_3 half cell with the mass and scan normalised ^{17}O integrals overlaid. In **(c)**, a single slice of the qCPMG spectra acquired from the in situ run (an Li metal/Li_2MnO_3 bag half-cell), recorded at 19.6 T; assignments are above each resonance are shown, alongside the intensity envelope used in the full in situ run, **(d)**. **(e)** shows the voltage profile obtained from the in situ run (top panel) with the normalised ^{17}O integral of the full qCPMG signal (bottom panel). Adapted with permission from ref. 69. Copyright 2022 Wiley-VCH GmbH.

References

(1) Keeler, J. *Understanding NMR Spectroscopy*, 2nd ed.; John Wiley & Sons, Ltd, 2010.

(2) Pell, A. J.; Pintacuda, G.; Grey, C. P. Paramagnetic NMR in Solution and the Solid State. *Prog. Nucl. Magn. Reson. Spectrosc.* **2019**, *111* (May), 1–271. https://doi.org/10.1016/j.pnmrs.2018.05.001.

(3) Wasylishen,R.; Ashbrook, S.; Wimperis, S. Vega, A. J., Quadrupolar Nuclei in Solids. In *NMR of Quadrupolar Nuclei in Solid Materials*; Wiley, 2012; pp 17–44.

(4) Duer, M. J. *Introduction to Solid-State NMR Spectroscopy*, 1st ed.; Blackwell Publishing Ltd, 2004.

(5) Kentgens, A. P. M. A Practical Guide to Solid-State NMR of Half-Integer Quadrupolar Nuclei with Some Applications to Disordered Systems. *Geoderma* **1997**, *80* (3–4), 271–306. https://doi.org/10.1016/S0016-7061(97)00056-6.

(6) Freude, D.; Haase, J.; Klinowski, J.; Carpenter, T. A.; Ronikier, G. NMR Line Shifts Caused by the Second-Order Quadrupolar Interaction. *Chem. Phys. Lett.* **1985**, *119* (4), 365–367.

(7) Ashbrook, S. E.; Smith, M. E. Solid State 17O NMR—an Introduction to the Background Principles and Applications to Inorganic Materials. *Chem. Soc. Rev.* **2006**, *35* (8), 718–735. https://doi.org/10.1039/B514051J.

(8) Abbad, A.; Bentounes, H. A.; Benstaali, W.; Bentata, S.; Bouadjemi, B. Influence of Hubbard Coefficient on the Structural, Electronic, Magnetic and Optical Properties of Mn/Fe Codoped ZnS. *Chalcogenide Lett.* **2015**, *12* (6), 301–312.

(9) Kim, J.; Middlemiss, D. S.; Chernova, N. A.; Zhu, B. Y. X.; Masquelier, C.; Grey, C. P. Linking Local Environments and Hyperfine Shifts: A Combined Experimental and Theoretical 31 P and 7 Li Solid-State NMR Study of Paramagnetic Fe(III) Phosphates. *J. Am. Chem. Soc.* **2010**, *132* (47), 16825–16840. https://doi.org/10.1021/ja102678r.

(10) Pell, A. J.; Clément, R. J.; Grey, C. P.; Emsley, L.; Pintacuda, G. Frequency-Stepped Acquisition in Nuclear Magnetic Resonance Spectroscopy under Magic Angle Spinning. *J. Chem. Phys.* **2013**, *138* (11), 114201. https://doi.org/10.1063/1.4795001.

(11) Geng, F.; Shen, M.; Hu, B.; Liu, Y.; Zeng, L.; Hu, B. Monitoring the Evolution of Local Oxygen Environments during LiCoO2 Charging: Via Ex Situ 17O NMR. *Chem. Commun.* **2019**, *55* (52), 7550–7553. https://doi.org/10.1039/c9cc03304a.

(12) Bastow, T. J.; Stuart, S. N. 17O NMR in Simple Oxides. *Chem. Phys.* **1990**, *143* (3), 459–467. https://doi.org/10.1016/0301-0104(90)87025-7.

(13) McCalla, E.; Abakumov, A. M.; Saubanère, M.; Foix, D.; Berg, E. J.; Rousse, G.; Doublet, M.-L.; Gonbeau, D.; Novák, P.; Van Tendeloo, G.; Dominko, R.; Tarascon, J.-M. Visualization of O-O Peroxo-like Dimers in High-Capacity Layered Oxides for Li-Ion Batteries. *Science* **2015**, *350* (6267), 1516–1521. https://doi.org/10.1126/science.aac8260.

(14) Sathiya, M.; Rousse, G.; Ramesha, K.; Laisa, C. P.; Vezin, H.; Sougrati, M. T.; Doublet, M.-L.; Foix, D.; Gonbeau, D.; Walker, W.; Prakash, A. S.; Ben Hassine, M.; Dupont, L.; Tarascon, J.-M. Reversible Anionic Redox Chemistry in High-Capacity Layered-Oxide Electrodes. *Nat. Mater.* **2013**, *12* (9), 827–835. https://doi.org/10.1038/nmat3699.

(15) Rozier, P.; Sathiya, M.; Paulraj, A.-R.; Foix, D.; Desaunay, T.; Taberna, P.-L.; Simon, P.; Tarascon, J.-M. Anionic Redox Chemistry in Na-Rich Na2Ru1−ySnyO3 Positive Electrode Material for Na-Ion Batteries. *Electrochem. Commun.* **2015**, *53*, 29–32. https://doi.org/10.1016/J.ELECOM.2015.02.001.

(16) Lebens-Higgins, Z. W.; Chung, H.; Zuba, M. J.; Rana, J.; Li, Y.; Faenza, N. V.; Pereira, N.; McCloskey, B. D.; Rodolakis, F.; Yang, W.; Whittingham, M. S.; Amatucci, G. G.; Meng, Y. S.; Lee, T. L.; Piper, L. F. J. How Bulk Sensitive Is Hard X-Ray Photoelectron Spectroscopy: Accounting for the Cathode-Electrolyte Interface When Addressing Oxygen Redox. *J. Phys. Chem. Lett.* **2020**, *11* (6), 2106–2112. https://doi.org/10.1021/acs.jpclett.0c00229.

(17) Lebens-Higgins, Z. W.; Vinckeviciute, J.; Wu, J.; Faenza, N. V.; Li, Y.; Sallis, S.; Pereira, N.; Meng, Y. S.; Amatucci, G. G.; Der Ven, A. V.; Yang, W.; Piper, L. F. J. Distinction

between Intrinsic and X-Ray-Induced Oxidized Oxygen States in Li-Rich 3d Layered Oxides and LiAlO2. *J. Phys. Chem. C* **2019**, *123* (21), 13201–13207. https://doi.org/10.1021/acs.jpcc.9b01298.
(18) Yang, W.; Devereaux, T. P. Anionic and Cationic Redox and Interfaces in Batteries: Advances from Soft X-Ray Absorption Spectroscopy to Resonant Inelastic Scattering. *J. Power Sources* **2018**, *389*, 188–197. https://doi.org/10.1016/j.jpowsour.2018.04.018.
(19) House, R. A.; Maitra, U.; Pérez-osorio, M. A.; Lozano, J. G.; Jin, L.; Somerville, J. W.; Duda, L. C.; Nag, A.; Walters, A.; Zhou, K.; Roberts, M. R.; Bruce, P. G. Superstructure Control of First-Cycle Voltage Hysteresis in O-Redox Cathodes. *Nature* **2019**, *577*, 502–508. https://doi.org/10.1038/s41586-019-1854-3.
(20) House, R. A.; Rees, G. J.; Pérez-Osorio, M. A.; Marie, J.-J.; Boivin, E.; Robertson, A. W.; Nag, A.; Garcia-Fernandez, M.; Zhou, K.-J.; Bruce, P. G. First-Cycle Voltage Hysteresis in Li-Rich 3d Cathodes Associated with Molecular O2 Trapped in the Bulk. *Nat. Energy* **2020**, *5*, 777–785. https://doi.org/10.1038/s41560-020-00697-2.
(21) Maitra, U.; House, R. A.; Somerville, J. W.; Tapia-Ruiz, N.; Lozano, J. G.; Guerrini, N.; Hao, R.; Luo, K.; Jin, L.; Pérez-Osorio, M. A.; Massel, F.; Pickup, D. M.; Ramos, S.; Lu, X.; McNally, D. E.; Chadwick, A. V.; Giustino, F.; Schmitt, T.; Duda, L. C.; Roberts, M. R.; Bruce, P. G. Oxygen Redox Chemistry without Excess Alkali-Metal Ions in Na2/3[Mg0.28Mn0.72]O2. *Nat. Chem.* **2018**, *10* (3), 288–295. https://doi.org/10.1038/nchem.2923.
(22) Saubanère, M.; McCalla, E.; Tarascon, J.-M.; Doublet, M.-L. The Intriguing Question of Anionic Redox in High-Energy Density Cathodes for Li-Ion Batteries. *Energy Environ. Sci.* **2016**, *9* (3), 984–991. https://doi.org/10.1039/C5EE03048J.
(23) Radin, M. D.; Vinckeviciute, J.; Seshadri, R.; Van der Ven, A. Manganese Oxidation as the Origin of the Anomalous Capacity of Mn-Containing Li-Excess Cathode Materials. *Nat. Energy* **2019**, *4*, 639–646. https://doi.org/10.1038/s41560-019-0439-6.
(24) Kitchaev, D. A.; Vinckeviciute, J.; Van Der Ven, A. Delocalized Metal−Oxygen π-Redox Is the Origin of Anomalous Nonhysteretic Capacity in Li-Ion and Na-Ion Cathode Materials. *J Am Chem Soc* **2021**, *143* (4), 1908–1916. https://doi.org/10.1021/jacs.0c10704.
(25) Grimaud, A.; Hong, W. T.; Shao-Horn, Y.; Tarascon, J.-M. Anionic Redox Processes for Electrochemical Devices. *Nat. Mater.* **2016**, *15* (2), 121–126. https://doi.org/10.1038/nmat4551.
(26) McCalla, E.; Sougrati, M. T.; Rousse, G.; Berg, E. J.; Abakumov, A.; Recham, N.; Ramesha, K.; Sathiya, M.; Dominko, R.; Van Tendeloo, G.; Novák, P.; Tarascon, J.-M. Understanding the Roles of Anionic Redox and Oxygen Release during Electrochemical Cycling of Lithium-Rich Layered Li 4 FeSbO 6. *J. Am. Chem. Soc.* **2015**, *137* (14), 4804–4814. https://doi.org/10.1021/jacs.5b01424.
(27) House, R. A.; Maitra, U.; Jin, L.; Lozano, J. G.; Somerville, J. W.; Rees, N. H.; Naylor, A. J.; Duda, L. C.; Massel, F.; Chadwick, A. V.; Ramos, S.; Pickup, D. M.; McNally, D. E.; Lu, X.; Schmitt, T.; Roberts, M. R.; Bruce, P. G. What Triggers Oxygen Loss in Oxygen Redox Cathode Materials? *Chem. Mater.* **2019**, *31* (9), 3293–3300. https://doi.org/10.1021/acs.chemmater.9b00227.
(28) Seo, D.-H.; Lee, J.; Urban, A.; Malik, R.; Kang, S.; Ceder, G. The Structural and Chemical Origin of the Oxygen Redox Activity in Layered and Cation-Disordered Li-Excess Cathode Materials. *Nat. Chem.* **2016**, *8* (7), 692–697. https://doi.org/10.1038/nchem.2524.
(29) Luo, K.; Roberts, M. R.; Hao, R.; Guerrini, N.; Pickup, D. M.; Liu, Y.-S.; Edström, K.; Guo, J.; Chadwick, A. V.; Duda, L. C.; Bruce, P. G. Charge-Compensation in 3d-Transition-Metal-Oxide Intercalation Cathodes through the Generation of Localized Electron Holes on Oxygen. *Nat. Chem.* **2016**, *8*. https://doi.org/10.1038/NCHEM.2471.
(30) Luo, K.; Roberts, M. R.; Guerrini, N.; Tapia-Ruiz, N.; Hao, R.; Massel, F.; Pickup, D. M.; Ramos, S.; Liu, Y.-S.; Guo, J.; Chadwick, A. V.; Duda, L. C.; Bruce, P. G. Anion Redox Chemistry in the Cobalt Free 3d Transition Metal Oxide Intercalation Electrode Li[Li 0.2

Ni 0.2 Mn 0.6]O 2. *J. Am. Chem. Soc.* **2016**, *138* (35), 11211–11218. https://doi.org/10.1021/jacs.6b05111.
(31) Hong, J.; Gent, W. E.; Xiao, P.; Lim, K.; Seo, D.-H.; Wu, J.; Csernica, P. M.; Takacs, C. J.; Nordlund, D.; Sun, C.-J.; Stone, K. H.; Passarello, D.; Yang, W.; Prendergast, D.; Ceder, G.; Toney, M. F.; Chueh, W. C. Metal–Oxygen Decoordination Stabilizes Anion Redox in Li-Rich Oxides. *Nat. Mater.* **2019**, *18* (3), 256–265. https://doi.org/10.1038/s41563-018-0276-1.
(32) Gent, W. E.; Lim, K.; Liang, Y.; Li, Q.; Barnes, T.; Ahn, S.-J.; Stone, K. H.; McIntire, M.; Hong, J.; Song, J. H.; Li, Y.; Mehta, A.; Ermon, S.; Tyliszczak, T.; Kilcoyne, D.; Vine, D.; Park, J.-H.; Doo, S.-K.; Toney, M. F.; Yang, W.; Prendergast, D.; Chueh, W. C. Coupling between Oxygen Redox and Cation Migration Explains Unusual Electrochemistry in Lithium-Rich Layered Oxides. *Nat. Commun.* **2017**, *8* (1), 2091–2091. https://doi.org/10.1038/s41467-017-02041-x.
(33) Gent, W. E.; Abate, I. I.; Yang, W.; Nazar, L. F.; Chueh, W. C. Design Rules for High-Valent Redox in Intercalation Electrodes. *Joule* **2020**, *4* (7), 1369–1397. https://doi.org/10.1016/j.joule.2020.05.004.
(34) Xie, Y.; Saubanère, M.; Doublet, M.-L. Requirements for Reversible Extra-Capacity in Li-Rich Layered Oxides for Li-Ion Batteries. *Energy Environ. Sci.* **2017**, *10* (1), 266–274. https://doi.org/10.1039/C6EE02328B.
(35) Mortemard de Boisse, B.; Liu, G.; Ma, J.; Nishimura, S.; Chung, S.-C.; Kiuchi, H.; Harada, Y.; Kikkawa, J.; Kobayashi, Y.; Okubo, M.; Yamada, A. Intermediate Honeycomb Ordering to Trigger Oxygen Redox Chemistry in Layered Battery Electrode. *Nat. Commun.* **2016**, *7*, 11397–11397. https://doi.org/10.1038/ncomms11397.
(36) Assat, G.; Iadecola, A.; Delacourt, C.; Dedryvère, R.; Tarascon, J.-M. Decoupling Cationic–Anionic Redox Processes in a Model Li-Rich Cathode via Operando X-Ray Absorption Spectroscopy. *Chem. Mater.* **2017**, *29* (22), 9714–9724. https://doi.org/10.1021/acs.chemmater.7b03434.
(37) Mortemard de Boisse, B.; Nishimura, S.; Watanabe, E.; Lander, L.; Tsuchimoto, A.; Kikkawa, J.; Kobayashi, E.; Asakura, D.; Okubo, M.; Yamada, A. Highly Reversible Oxygen-Redox Chemistry at 4.1 V in Na4/7−x[□1/7Mn6/7]O2 (□: Mn Vacancy). *Adv. Energy Mater.* **2018**, *8* (20), 1800409–1800409.
(38) Li, Y.; Wang, X.; Gao, Y.; Zhang, Q.; Tan, G.; Kong, Q.; Bak, S.; Lu, G.; Yang, X.-Q.; Gu, L.; Lu, J.; Amine, K.; Wang, Z.; Chen, L. Native Vacancy Enhanced Oxygen Redox Reversibility and Structural Robustness. *Adv. Energy Mater.* **2019**, *9* (4), 1803087. https://doi.org/10.1002/aenm.201803087.
(39) Pearce, P. E.; Perez, A. J.; Rousse, G.; Saubanère, M.; Batuk, D.; Foix, D.; McCalla, E.; Abakumov, A. M.; Van Tendeloo, G.; Doublet, M. L.; Tarascon, J. M. Evidence for Anionic Redox Activity in a Tridimensional-Ordered Li-Rich Positive Electrode β-Li 2 IrO 3. *Nat. Mater.* **2017**, *16* (5), 580–586. https://doi.org/10.1038/nmat4864.
(40) Adamczyk, E.; Pralong, V. Na 2 Mn 3 O 7 : A Suitable Electrode Material for Na-Ion Batteries? *Chem. Mater.* **2017**, *29* (11), 4645–4648. https://doi.org/10.1021/acs.chemmater.7b01390.
(41) Larsen, F. H.; Jakobsen, H. J.; Ellis, P. D.; Nielsen, N. C. QCPMG-MAS NMR of Half-Integer Quadrupolar Nuclei. *J. Magn. Reson.* **1998**, *131* (1), 144–147. https://doi.org/10.1006/JMRE.1997.1341.
(42) O'Dell, L. A.; Schurko, R. W. QCPMG Using Adiabatic Pulses for Faster Acquisition of Ultra-Wideline NMR Spectra. *Chem. Phys. Lett.* **2008**, *464* (1–3), 97–102. https://doi.org/10.1016/J.CPLETT.2008.08.095.
(43) Märker, K.; Xu, C.; Grey, C. P. Operando NMR of NMC811/Graphite Lithium-Ion Batteries: Structure, Dynamics, and Lithium Metal Deposition. *J. Am. Chem. Soc.* **2020**, *142* (41), 17447–17456. https://doi.org/10.1021/JACS.0C06727/SUPPL_FILE/JA0C06727_SI_001.PDF.
(44) Faenza, N. V.; Bruce, L.; Lebens-Higgins, Z. W.; Plitz, I.; Pereira, N.; Piper, L. F. J.; Amatucci, G. G. Editors' Choice-Growth of Ambient Induced Surface Impurity Species

on Layered Positive Electrode Materials and Impact on Electrochemical Performance. *J. Electrochem. Soc.* **2017**, *164* (14), A3727–A3741. https://doi.org/10.1149/2.0921714jes.
(45) Reeves, P. J.; Seymour, I. D.; Griffith, K. J.; Grey, C. P. Characterizing the Structure and Phase Transition of Li 2 RuO 3 Using Variable-Temperature 17 O and 7 Li NMR Spectroscopy. *Chem. Mater.* **2019**, *31* (8), 2814–2821. https://doi.org/10.1021/acs.chemmater.8b05178.
(46) Seymour, I. D.; Middlemiss, D. S.; Halat, D. M.; Trease, N. M.; Pell, A. J.; Grey, C. P. Characterizing Oxygen Local Environments in Paramagnetic Battery Materials via 17O NMR and DFT Calculations. *J. Am. Chem. Soc.* **2016**, *138* (30), 9405–9408. https://doi.org/10.1021/jacs.6b05747.
(47) Liu, H.; Liu, H.; Seymour, I. D.; Chernova, N.; Wiaderek, K. M.; Trease, N. M.; Hy, S.; Chen, Y.; An, K.; Zhang, M.; Borkiewicz, O. J.; Lapidus, S. H.; Qiu, B.; Xia, Y.; Liu, Z.; Chupas, P. J.; Chapman, K. W.; Whittingham, M. S.; Grey, C. P.; Meng, Y. S. Identifying the Chemical and Structural Irreversibility in LiNi 0.8 Co 0.15 Al 0.05 O 2 – a Model Compound for Classical Layered Intercalation. *J. Mater. Chem. A* **2018**, *6* (9), 4189–4198. https://doi.org/10.1039/C7TA10829J.
(48) Tong, Y. Y. Nuclear Spin-Echo Fourier-Transform Mapping Spectroscopy for Broad NMR Lines in Solids. *J. Magn. Reson. A* **1996**, *119* (1), 22–28. https://doi.org/10.1006/JMRA.1996.0047.
(49) Dogan, F.; Vaughey, J. T.; Iddir, H.; Key, B. Direct Observation of Lattice Aluminum Environments in Li Ion Cathodes LiNi1-y-ZCoyAlzO2 and Al-Doped LiNixMnyCozO2 via 27Al MAS NMR Spectroscopy. *ACS Appl. Mater. Interfaces* **2016**, *8* (26), 16708–16717. https://doi.org/10.1021/ACSAMI.6B04516/SUPPL_FILE/AM6B04516_SI_001.PDF.
(50) Massiot, D.; Fayon, F.; Capron, M.; King, I.; Le Calvé, S.; Alonso, B.; Durand, J.-O.; Bujoli, B.; Gan, Z.; Hoatson, G. Modelling One- and Two-Dimensional Solid-State NMR Spectra. *Magn. Reson. Chem.* **2002**, *40* (1), 70–76. https://doi.org/10.1002/mrc.984.
(51) Grenier, A.; Reeves, P. J.; Liu, H.; Seymour, I. D.; Märker, K.; Wiaderek, K. M.; Chupas, P. J.; Grey, C. P.; Chapman, K. W. Intrinsic Kinetic Limitations in Substituted Lithium Layered Transition-Metal Oxide Electrodes. *J. Am. Chem. Soc.* **2020**, *142* (15), 7001–7011. https://doi.org/10.1021/jacs.9b13551.
(52) MacKenzie, K.; Smith, M. E. *Multinuclear Solid-State Nuclear Magnetic Resonance of Inorganic Materials*; 2002.
(53) Trease, N. M.; Seymour, I. D.; Radin, M. D.; Liu, H.; Liu, H.; Hy, S.; Chernova, N.; Parikh, P.; Devaraj, A.; Wiaderek, K. M.; Chupas, P. J.; Chapman, K. W.; Whittingham, M. S.; Meng, Y. S.; Van Der Van, A.; Grey, C. P. Identifying the Distribution of Al 3+ in LiNi 0.8 Co 0.15 Al 0.05 O 2. *Chem. Mater.* **2016**, *28* (22), 8170–8180. https://doi.org/10.1021/acs.chemmater.6b02797.
(54) Capitaine, F.; Gravereau, P.; Delmas, C. A New Variety of LiMnO2 with a Layered Structure. *Solid State Ion.* **1996**, *89* (3–4), 197–202. https://doi.org/10.1016/0167-2738(96)00369-4.
(55) Bruce, P. G.; Armstrong, A. R.; Gitzendanner, R. L. New Intercalation Compounds for Lithium Batteries: Layered LiMnO 2. *J. Mater. Chem.* **1999**, *9* (1), 193–198. https://doi.org/10.1039/A803938K.
(56) Vernay, F.; Penc, K.; Fazekas, P.; Mila, F. Orbital Degeneracy as a Source of Frustration in LiNiO2. *Phys. Rev. B - Condens. Matter Mater. Phys.* **2004**, *70* (1), 014428. https://doi.org/10.1103/PHYSREVB.70.014428/FIGURES/17/MEDIUM.
(57) Faenza, N. V.; Pereira, N.; Halat, D. M.; Vinckeviciute, J.; Bruce, L.; Radin, M. D.; Mukherjee, P.; Badway, F.; Halajko, A.; Cosandey, F.; Grey, C. P.; Van Der Ven, A.; Amatucci, G. G. Phase Evolution and Degradation Modes of R3 m LixNi1- y- ZCoyAlzO2 Electrodes Cycled Near Complete Delithiation. *Chem. Mater.* **2018**, *30* (21), 7545–7574. https://doi.org/10.1021/ACS.CHEMMATER.8B02720/SUPPL_FILE/CM8B02720_SI_001.PDF.
(58) Dupree, R.; Lewis, M. H.; Smith, M. E. Structural Characterization of Ceramic Phases with High-Resolution 27Al NMR. *J. Appl. Crystallogr.* **1988**, *21* (2), 109–116. https://doi.org/10.1107/S0021889887010069.

(59) Yamasaki, A. Cobalt-59 Nuclear Magnetic Resonance Spectroscopy in Coordination Chemistry. *J. Coord. Chem.* **1991**, *24* (3), 211–260. https://doi.org/10.1080/00958979109407886.
(60) Emsley, J. W.; Feeney, J.; Sutcliffe, L. H. *High Resolution Nuclear Magnetic Resonance Spectroscopy. Volume 2*; 1966; Vol. 2.
(61) Yoon, W. S.; Paik, Y.; Yang, X. Q.; Balasubramanian, M.; McBreen, J.; Grey, C. P. Investigation of the Local Structure of the LiNi0.5Mn0.5O2 Cathode Material during Electrochemical Cycling by X-Ray Absorption and NMR Spectroscopy. *Electrochem. Solid-State Lett.* **2002**, *5* (11), A263. https://doi.org/10.1149/1.1513001/XML.
(62) Oh, J. M.; Hwang, S. H.; Choy, J. H. The Effect of Synthetic Conditions on Tailoring the Size of Hydrotalcite Particles. *Solid State Ion.* **2002**, *151* (1–4), 285–291. https://doi.org/10.1016/S0167-2738(02)00725-7.
(63) Nakai, I.; Takahashi, K.; Shiraishi, Y.; Nakagome, T.; Nishikawa, F. Study of the Jahn–Teller Distortion in LiNiO2, a Cathode Material in a Rechargeable Lithium Battery, Byin SituX-Ray Absorption Fine Structure Analysis. *J. Solid State Chem.* **1998**, *140* (1), 145–148. https://doi.org/10.1006/JSSC.1998.7943.
(64) Yoon, W. S.; Kim, K. B.; Kim, M. G.; Lee, M. K.; Shin, H. J.; Lee, J. M.; Lee, J. S.; Yo, C. H. Oxygen Contribution on Li-Ion Intercalation−Deintercalation in LiCoO2 Investigated by O K-Edge and Co L-Edge X-Ray Absorption Spectroscopy. *J. Phys. Chem. B* **2002**, *106* (10), 2526–2532. https://doi.org/10.1021/JP013735E.
(65) Lebens-Higgins, Z. W.; Faenza, N. V.; Radin, M. D.; Liu, H.; Sallis, S.; Rana, J.; Vinckeviciute, J.; Reeves, P. J.; Zuba, M. J.; Badway, F.; Pereira, N.; Chapman, K. W.; Lee, T.-L.; Wu, T.; Grey, C. P.; Melot, B. C.; Van Der Ven, A.; Amatucci, G. G.; Yang, W.; Piper, L. F. J. Revisiting the Charge Compensation Mechanisms in LiNi0.8Co0.2−yAlyO2 Systems. *Mater. Horiz.* **2019**, *6* (10), 2112–2123. https://doi.org/10.1039/C9MH00765B.
(66) Geng, F.; Hu, B.; Li, C.; Zhao, C.; Lafon, O.; Trébosc, J.; Amoureux, J. P.; Shen, M.; Hu, B. Anionic Redox Reactions and Structural Degradation in a Cation-Disordered Rock-Salt Li1.2Ti0.4Mn0.4O2cathode Material Revealed by Solid-State NMR and EPR. *J. Mater. Chem. A* **2020**, *8* (32), 16515–16526. https://doi.org/10.1039/d0ta03358h.
(67) Fukuzumi, S.; Ohkubo, K. Quantitative Evaluation of Lewis Acidity of Metal Ions Derived from the g Values of ESR Spectra of Superoxide: Metal Ion Complexes in Relation to the Promoting Effects in Electron Transfer Reactions. *Chem Eur J* **2000**, *6* (24), 4532–4535.
(68) Dundon, J. M. 17O NMR in Liquid O2. *J. Chem. Phys.* **1982**, *76* (5), 2171–2173. https://doi.org/10.1063/1.443233.
(69) Li, X.; Li, X.; Monluc, L.; Chen, B.; Tang, M.; Chien, P.-H.; Feng, X.; Hung, I.; Gan, Z.; Urban, A.; Hu, Y.-Y.; Li, X.; Monluc, L.; Chen, B.; Tang, M.; Chien, P.-H.; Feng, X.; Hu, Y.-Y.; Urban, A.; Hung, I.; Gan, Z. Stacking-Fault Enhanced Oxygen Redox in Li2MnO3. *Adv. Energy Mater.* **2022**, *12* (18), 2200427. https://doi.org/10.1002/AENM.202200427.
(70) Wang, R.; He, X.; He, L.; Wang, F.; Xiao, R.; Gu, L.; Li, H.; Chen, L. Atomic Structure of Li2MnO3 after Partial Delithiation and Re-Lithiation. *Adv. Energy Mater.* **2013**, *3* (10), 1358–1367. https://doi.org/10.1002/AENM.201200842.
(71) Shimoda, K.; Oishi, M.; Matsunaga, T.; Murakami, M.; Yamanaka, K.; Arai, H.; Ukyo, Y.; Uchimoto, Y.; Ohta, T.; Matsubara, E.; Ogumi, Z. Direct Observation of Layered-to-Spinel Phase Transformation in Li2MnO3 and the Spinel Structure Stabilised after the Activation Process. *J. Mater. Chem. A* **2017**, *5* (14), 6695–6707. https://doi.org/10.1039/C6TA11151C.
(72) Dogan, F.; Croy, J. R.; Balasubramanian, M.; Slater, M. D.; Iddir, H.; Johnson, C. S.; Vaughey, J. T.; Key, B. Solid State NMR Studies of Li 2 MnO 3 and Li-Rich Cathode Materials: Proton Insertion, Local Structure, and Voltage Fade. *J. Electrochem. Soc.* **2015**, *162* (1), A235–A243. https://doi.org/10.1149/2.1041501jes.
(73) Rana, J.; Papp, J. K.; Lebens-Higgins, Z.; Zuba, M.; Kaufman, L. A.; Goel, A.; Schmuch, R.; Winter, M.; Whittingham, M. S.; Yang, W.; McCloskey, B. D.; Piper, L. F. J. Quantifying the Capacity Contributions during Activation of Li2MnO3. *ACS Energy Lett.* **2020**, *5* (2), 634–641. https://doi.org/10.1021/acsenergylett.9b02799.

(74) Yan, P.; Xiao, L.; Zheng, J.; Zhou, Y.; He, Y.; Zu, X.; Mao, S. X.; Xiao, J.; Gao, F.; Zhang, J. G.; Wang, C. M. Probing the Degradation Mechanism of Li2MnO3 Cathode for Li-Ion Batteries. *Chem. Mater.* **2015**, *27* (3), 975–982. https://doi.org/10.1021/cm504257m.
